# Defining and Estimating Outcomes Directly Averted by a Vaccination Program when Rollout Occurs Over Time

**Published:** 2025-10-08

**Authors:** Katherine M Jia, Christopher B Boyer, Alyssa Bilinski, Marc Lipsitch

**Affiliations:** 1 Center for Communicable Disease Dynamics, Department of Epidemiology, Harvard T.H. Chan School of Public Health, Boston, Massachusetts, USA; 2 Department of Quantitative Health Sciences, Cleveland Clinic Research, Cleveland, Ohio, USA; 3 Department of Medicine, Cleveland Clinic Lerner College of Medicine of Case Western Reserve University, Cleveland, Ohio, USA; 4 Department of Health Services, Policy, and Practice, Brown University School of Public Health, Providence, Rhode Island, USA; 5 Department of Biostatistics, Brown University School of Public Health, Providence, Rhode Island, USA; 6 Department of Immunology and Infectious Diseases, Harvard T.H. Chan School of Public Health, Boston, Massachusetts, USA

**Keywords:** vaccine-averted outcomes, cumulative incidence difference, direct impact, overall impact

## Abstract

During the COVID-19 pandemic, estimating the total deaths averted by vaccination has been of great public health interest. Instead of estimating total deaths averted by vaccination among both vaccinated and unvaccinated individuals, some studies empirically estimated only “directly averted” deaths among vaccinated individuals, typically suggesting that vaccines prevented more deaths overall than directly due to the indirect effect. Here, we define the causal estimand to quantify outcomes “directly averted” by vaccination—i.e., the impact of vaccination for vaccinated individuals, holding vaccination coverage fixed—for vaccination at multiple time points, and show that this estimand is a lower bound on the total outcomes averted when the indirect effect is non-negative. We develop an unbiased estimator for the causal estimand in a one-stage randomized controlled trial (RCT) and explore the bias of a popular “hazard difference” estimator frequently used in empirical studies. We show that even in an RCT, the hazard difference estimator is biased if vaccination has a non-null effect, as it fails to incorporate the greater depletion of susceptibles among the unvaccinated individuals. In simulations, the overestimation is small for averted deaths when infection-fatality rate is low, as for many important pathogens. However, the overestimation can be large for averted infections given a high basic reproduction number. Additionally, we define and compare estimand and estimators for avertible outcomes (i.e., outcomes that could have been averted by vaccination, but were not due to failure to vaccinate). Future studies can explore the identifiability of the causal estimand in observational settings.

## 1 Introduction

During the COVID-19 pandemic, determining the total number of infections (or deaths) averted by vaccination has been of great public health interest.^1–10^ Researchers are interested in how many infections (or deaths) have been averted overall by COVID-19 vaccine rollout programs, compared to the counterfactual of no vaccination for anyone. However, in the presence of indirect effects, the key challenge is that we may not observe a comparable population that is unvaccinated throughout. Rather than estimating the total number of outcomes averted among both vaccinated and unvaccinated individuals, some empirical studies^4–8^ have instead estimated outcomes “directly averted” among vaccinated individuals by conditioning on the actual vaccination coverage in the rest of the population. The estimand and estimation procedures used in selected examples of studies are summarized in eAppendix 1. Typically, studies that estimated outcomes directly averted among vaccinated individuals computed daily or weekly hazards of death among all vaccinated and not-yet-vaccinated individuals, calculated the difference in hazards, multiplied this difference with the number of vaccinated survivors, and summed the results across time. We refer to this method as the “hazard difference estimator.” Empirical analyses of this type commonly assumed that such directly averted outcomes are a lower bound on the total averted outcomes due to the indirect effect in reducing transmission.^4–6^

This study is motivated by two research gaps from the numerous empirical analyses^4–8,11,12^ that estimated directly averted (or avertible) outcomes among vaccinated (or unvaccinated) individuals under a vaccine rollout. First, the causal estimand for directly averted outcomes has not been precisely defined as a mathematical quantity for vaccination at multiple time points under interference. Second, the popular hazard difference estimator used by these analyses has not been evaluated. Therefore, we first propose the casual estimand and its unbiased estimator based on a one-stage randomized controlled trial (RCT). We use the causal estimand to formalize the lower bound assumption and identify the condition under which a vaccine rollout program has averted more outcomes overall than directly among the vaccinated individuals. Last, we evaluate the bias of the hazard difference estimator relative to the causal estimand. This paper is an important extension to our previous study^13^ which develops estimands for quantifying averted outcomes for vaccination at a single time point, as vaccination almost always occurs over time in reality.

Section 2 of this paper describes the setup and notation. Section 3 defines the causal estimand for quantifying directly averted outcomes among vaccinated individuals, as well as its unbiased estimator and the hazard difference estimator. Section 4 examines the bias of the hazard difference estimator both analytically and through simulations.

## 2 Setup and notation

Hudgens and Halloran^14^ defined four *effect* estimands (namely the direct, indirect, total, and overall effects) for vaccination at a single time point in a two-stage randomized trial, which reduces to a one-stage RCT when the study population consists of a single group. In reality, two-stage RCTs are rarely conducted or justified; for our purpose of estimating directly averted outcomes, we define our estimand and estimators based on a one-stage RCT, with notation closely aligned to that of Hudgens and Halloran.^14^

Consider a one-stage RCT that consists of N individuals indexed by j=1,…,N with a large N.^14^ Consider q+2 evenly spaced measurement intervals for q∈ℕ. Let l∈{0,…,q+1} denote each interval with baseline measurements taken in interval 0, and q+1 representing the end of follow-up.^15^ Let $X_{j}$ denote the assigned vaccination time, where $x_{j}\in \{0,\ldots ,q+1\}$ are possible realizations of $X_{j}$.^16^ Vaccination occurs *at the beginning* of each interval, whereas $x_{j}=q+1$ denotes unvaccinated throughout. Let $X=\left(X_{1},\ldots ,X_{N}\right)$ denote the vaccination times individuals were assigned. Let x denote a possible realization of X. Let X(N) denote the set of all possible ${\left(q+1\right)}^{N}$ vaccination time allocations for the group, for which x∈X(N). Throughout we assume perfect compliance (i.e., assignment to a particular vaccination time is equivalent to receipt of vaccination at that time if the person is still alive), no loss-to-follow-up, and no measurement error. In an ideal one-stage RCT, these assumptions are expected to hold.

Here, interference is assumed—that is, the potential outcome for any individual depend on vaccination assignments of every other individual in the group.^14^ Let $Y_{q+1,j}(x)\in \{0,1\}$ be a *cumulative* indicator of experiencing the outcome (e.g., death) *before the beginning* of interval q+1 for individual j had the group followed the vaccination schedule x∈X(N). By convention, $Y_{0,j}\equiv 0$.^17^

Let $\text{ρ}=\left\{q+1,d;\rho_{0},\ldots ,\rho_{q}\right\}$ denote parameterizations that govern the distribution of X, where q+1 is the number of potential vaccination times, d is the number of days of each interval, $\rho_{x}$ is the proportion of individuals assigned to x∈{0,…,q+1} such that $\sum_{x=0}^{q+1}\rho_{x}=1$. We assume ρ is a *mixed individual assignment strategy*,^14,18^ as defined in eAppendix 2. In words, ρ randomly assigns $\rho_{0}\times 100\%$ of the individuals to receive vaccination at baseline, $\rho_{1}\times 100\%$ to receive vaccination at the beginning of interval 1, and so on, with $\rho_{q+1}\times 100\%$ to remain unvaccinated throughout.^18^ Let ϕ={q+1,d;0} denote no vaccination. To quantify vaccine-averted outcomes, our goal is to assess the impact of some vaccination strategy ρ compared to ϕ.

At baseline, individuals are randomly assigned to X conditional on a mixed individual assignment strategy (i.e., fixed proportions of individuals [e.g., 20%, 30%, 50%] were assigned to receive vaccination at specific times [e.g., Day 0, Day 60, Day 120], respectively; see definition in eAppendix 2). Note that random assignments X occurs only at baseline, even though individuals receive vaccination at different times. This study design is referred to as a “one-stage” RCT because randomization takes place only once, in contrast to the “two-stage” RCT described by Hudgens and Halloran,^14^ in which groups are first randomized to different strategies and then individuals within each group are randomized to vaccination conditional on their group’s assigned strategy. Let ${\bar{Y}}_{q+1}(x;\text{ρ})$ denote the group average potential outcome as defined in eAppendix 2, which is equivalent to population average potential outcome^14^ because there is only one group. Let $\text{Δ}{\bar{Y}}_{l+1}(x;\text{ρ})={\bar{Y}}_{l+1}(x;\text{ρ})-{\bar{Y}}_{l}(x;\text{ρ})$ for l∈{0,…,q} be the difference in $\bar{Y}$ between intervals l and l+1.^19^

## 3 Causal estimand and estimators for quantifying outcomes directly averted by vaccination

### 3.1 Causal estimand

In our prior work with vaccination at a single time point,^13^ we defined the “direct impact” estimand to quantify outcomes directly averted among vaccinated individuals as the number of vaccinated individuals multiplied by the direct effect (DE)^14^ for vaccination at the baseline time point. In notation, the number of outcomes directly averted by $\text{ρ}=\left\{1,d;\rho_{0}\right\}$ (i.e, vaccination of $\rho_{0}$ at the beginning of a single interval, with interval duration d), compared to no vaccination ϕ={1,d;0}, is:

(1) $$\begin{array}{l} \delta_{1}^{D}(\phi ,\text{ρ})=N\rho_{0}\left({\bar{Y}}_{1}(1;\text{ρ})-{\bar{Y}}_{1}(0;\text{ρ})\right)\\ \;=N\rho_{0}DE_{1}((1,0);\text{ρ}). \end{array}$$

When vaccination occurs at multiple time points, there could be multiple versions of direct effects. A direct effect can be a contrast between non-vaccination and some vaccination time $x^{\prime }\in \{0,\ldots ,q\}$, conditional on ρ (e.g., comparing individuals unvaccinated versus vaccinated at interval 0, or unvaccinated versus vaccinated at interval 1). In notation, the direct effect comparing probability of having developed the outcome by the beginning of interval q+1 for an individual unvaccinated throughout versus assigned to $x^{\prime }$ when the group follows strategy $\text{ρ}=\left\{q+\left.1,d;\rho_{0},\ldots ,\rho_{q}\right\}\right.$ is:

(2) $$DE_{q+1}\left(\left(q+1,x^{\prime }\right);\text{ρ}\right)={\bar{Y}}_{q+1}(q+1;\text{ρ})-{\bar{Y}}_{q+1}\left(x^{\prime };\text{ρ}\right).$$

Now, extending the estimand in equation (1) for vaccination at two time points (i.e., $x^{\prime }=0$ or $x^{\prime }=1$, as compared to unvaccinated x=2), we consider the direct effect of $x^{\prime }\in \{0,1\}$ compared to unvaccinated throughout, weighted by the number of individuals assigned with $x^{\prime }$, and summed across $x^{\prime }\in \{0,1\}$. In notation, the number of outcomes directly averted by $\text{ρ}=\left\{2,d;\rho_{0},\rho_{1}\right\}$, compared to no vaccination ϕ={2,d;0}, is:

#### Definition 1 (Causal estimand for directly averted outcomes for vaccination at two time points)


(3) $$\begin{array}{l} \delta_{2}^{D}(\phi ,\text{ρ})=N\cdot \left\{\rho_{0}\cdot \left[{\bar{Y}}_{2}(2;\text{ρ})-{\bar{Y}}_{2}(0;\text{ρ})\right]+\rho_{1}\cdot \left[{\bar{Y}}_{2}(2;\text{ρ})-{\bar{Y}}_{2}(1;\text{ρ})\right]\right\}\\ \;=N\cdot \left\{\rho_{0}\cdot DE_{2}((2,0);\text{ρ})+\rho_{1}\cdot DE_{2}((2,1);\text{ρ})\right\}. \end{array}$$

eAppendix 3 extends $\delta_{2}^{D}(\phi ,\text{ρ})$ to an arbitrary number of vaccination times. eAppendix 4 shows that $\delta_{q+1}^{D}(\phi ,\text{ρ})$ is the lower bound on outcomes averted among both vaccinated and unvaccinated individuals when the indirect effect is non-negative. However, the indirect effect can be negative when transmission or fatality parameters vary over time, as we showed earlier for vaccination at a single time point.^13^ Therefore, in the more general case of vaccination at multiple time points, the indirect effect is not guaranteed to be non-negative under many realistic scenarios.

### 3.2 Unbiased estimator

Define ${\hat{Y}}_{2}(x;\text{ρ})=\frac{\sum_{j=1}^{N}Y_{2,j}(X)I\left[X_{j}=x\right]}{\sum_{j=1}^{N}I\left[X_{j}=x\right]}$ for x∈{0,1,2}. That is, ${\hat{Y}}_{2}(x;\text{ρ})$ is the cumulative incidence by the beginning of interval 2 for individuals assigned to x under strategy ρ. Then the estimator for $\delta_{2}^{D}(\phi ,\text{ρ})$ in equation (3) is:

(4) $${\hat{\delta }}_{2}^{D}(\phi ,\text{ρ})=N\cdot \left\{\rho_{0}\cdot \left[{\hat{Y}}_{2}(2;\text{ρ})-{\hat{Y}}_{2}(0;\text{ρ})\right]+\rho_{1}\cdot \left[{\hat{Y}}_{2}(2;\text{ρ})-{\hat{Y}}_{2}(1;\text{ρ})\right]\right\}.$$

eAppendix 5 proves that ${\hat{\delta_{q+1}}}^{D}(\phi ,\text{ρ})$ is an unbiased estimator for $\delta_{q+1}^{D}(\phi ,\text{ρ})$ for q∈ℕ in a one-stage RCT under mixed assignment strategy ρ. Note that $\rho_{x}$ for x∈{0,1,…,q+1} is known and fixed under a mixed assignment strategy ρ in an RCT, although it must be estimated in an observational setting.

### 3.3 Hazard difference estimator

As summarized in the literature review (eAppendix 1), recent empirical studies^4–8^ often used what we refer to as the *hazard difference* estimator, which relies on the number at risk and the number of new cases among individuals vaccinated (and not-yet-vaccinated) by a given time to quantify outcomes directly averted among vaccinated individuals.

Let $\Delta {\hat{Y}}_{l+1}(x;\text{ρ})={\hat{Y}}_{l+1}(x;\text{ρ})-{\hat{Y}}_{l}(x;\text{ρ})$ for l∈{0,…,q}. Equation (5) defines the hazard difference estimator for two vaccination times (see eAppendix 6 for extension to an arbitrary number of vaccination times).

#### Definition 2 (Hazard difference estimator for directly averted outcomes for vaccination at two time points)


(5) $${\hat{\delta }}_{2}^{D*}(\phi ,\text{ρ})={\hat{N}}_{0}^{v}(\text{ρ})\left({\hat{h}}_{1}^{u}(\text{ρ})-{\hat{h}}_{1}^{v}(\text{ρ})\right)+{\hat{N}}_{1}^{v}(\text{ρ})\left({\hat{h}}_{2}^{u}(\text{ρ})-{\hat{h}}_{2}^{v}(\text{ρ})\right)$$

where ${\hat{h}}_{1}^{v}(\text{ρ})=\text{Δ}{\hat{Y}}_{1}(0;\text{ρ})$ is the incidence among vaccinated individuals by the beginning of interval 1, ${\hat{h}}_{1}^{u}(\text{ρ})=\frac{\rho_{1}\text{Δ}{\hat{Y}}_{1}(1;\text{ρ})+\rho_{2}\text{Δ}{\hat{Y}}_{1}(2;\text{ρ})}{\rho_{1}+\rho_{2}}$ is the incidence among not-yet-vaccinated individuals by the beginning of interval 1 (Note this quantity is the combination of two distinct groups—individuals assigned to x=1 and x=2), ${\hat{N}}_{0}^{v}(\text{ρ})=N\rho_{0}$ is the number survived by the beginning of interval 0 among individuals assigned to x=0, ${\hat{N}}_{1}^{v}(\text{ρ})=N\left[\rho_{0}\left(1-{\hat{Y}}_{1}(0;\text{ρ})\right)+\rho_{1}(1-\right.\left.\left.{\hat{Y}}_{1}(1;\text{ρ})\right)\right]$ is the combined number survived by the beginning of interval 1 among individuals assigned to x=0 and x=1, ${\hat{h}}_{2}^{v}(\text{ρ})=\frac{\rho_{0}\text{Δ}{\hat{Y}}_{2}(0;\text{ρ})+\rho_{1}\text{Δ}{\hat{Y}}_{2}(1;\text{ρ})}{\rho_{0}\left(1-{\hat{Y}}_{1}(0;\text{ρ})\right)+\rho_{1}\left(1-{\hat{Y}}_{1}(1;\text{ρ})\right)}$ is the hazard among vaccinated individuals by the beginning of interval 2, ${\hat{h}}_{2}^{u}(\text{ρ})=\frac{\text{Δ}{\hat{Y}}_{2}(2;\text{ρ})}{1-{\hat{Y}}_{1}(2;\text{ρ})}$ is the hazard among not-yet-vaccinated individuals by the beginning of interval 2.

**FIGURE 1 |:**
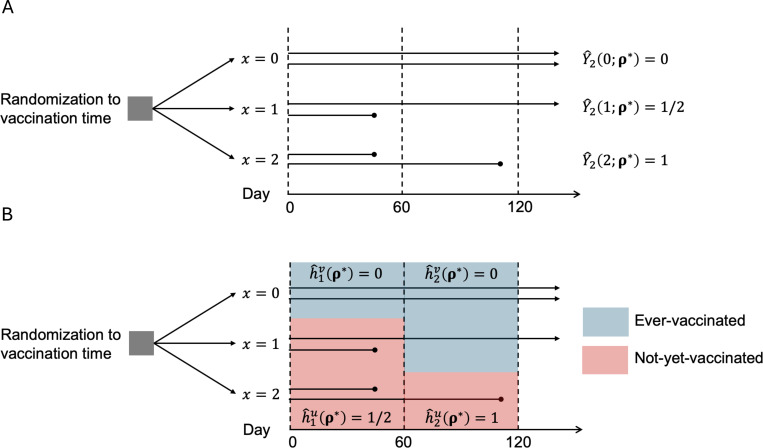
Schematic representation of an ideal randomized controlled trial with 6 individuals (horizontal lines) under the strategy ${\text{ρ}}^{*}=\left\{2,60;\frac{1}{3},\frac{1}{3}\right\}$. One-third of individuals are randomly assigned to vaccination at baseline $\left(x=0\right)$, one third to vaccination at the beginning of interval 1 $\left(x=1\right)$, and the remaining to no vaccination throughout $\left(x=2\right)$. The dashed vertical lines represent the beginning of each interval. (A) The unbiased estimator considers the cumulative incidence in each arm. Note ${\hat{Y}}_{l}\left(x;{\text{ρ}}^{*}\right)$ is the cumulative incidence by the beginning of interval l among individuals assigned to vaccination time x under strategy ${\text{ρ}}^{*}$. (B) Hazard difference estimator considers the hazards and survival among vaccinated and not-yet-vaccinated individuals. Note ${\hat{h}}_{l+1}^{v}(\cdot )$ (or ${\hat{h}}_{l+1}^{u}(\cdot )$) is the hazard for vaccinated (or not-yet-vaccinated) individuals from interval l to l+1.

To give intuition for the unbiased estimator and the hazard difference estimator, consider the following example (Figure 1). Suppose we observe an ideal trial randomizing 6 individuals into three arms according to the strategy ${\text{ρ}}^{*}=\left\{2,60;\frac{1}{3},\frac{1}{3}\right\}$. One-third of the individuals are randomly assigned to vaccination at baseline $\left(x=0\right)$, one-third to vaccination at the beginning of interval 1 $\left(x=1\right)$, and the remaining to no vaccination throughout $\left(x=2\right)$. As illustrated in Figure 1A, the unbiased estimator gives ${\hat{\delta }}_{2}^{D}\left(\phi ,{\text{ρ}}^{*}\right)=6\cdot \left\{\rho_{0}\cdot \left[{\hat{Y}}_{2}\left(2;{\text{ρ}}^{*}\right)-{\hat{Y}}_{2}\left(0;{\text{ρ}}^{*}\right)\right]+\rho_{1}\cdot \left[{\hat{Y}}_{2}\left(2;{\text{ρ}}^{*}\right)-\right.\right.\left.\left.{\hat{Y}}_{2}\left(1;{\text{ρ}}^{*}\right)\right]\right\}=6\cdot \left[\frac{1}{3}\cdot (1-0)+\frac{1}{3}\cdot \left(1-\frac{1}{2}\right)\right]=3$. As illustrated in Figure 1B, the hazard difference estimator gives ${\hat{\delta }}_{2}^{D*}\left(\phi ,{\text{ρ}}^{*}\right)={\hat{N}}_{0}^{v}\left({\text{ρ}}^{*}\right)\left({\hat{h}}_{1}^{u}\left({\text{ρ}}^{*}\right)-{\hat{h}}_{1}^{v}\left({\text{ρ}}^{*}\right)\right)+{\hat{N}}_{1}^{v}\left({\text{ρ}}^{*}\right)\left({\hat{h}}_{2}^{u}\left({\text{ρ}}^{*}\right)-\right.\left.{\hat{h}}_{2}^{v}\left({\text{ρ}}^{*}\right)\right)=2\cdot \left(\frac{1}{2}-0\right)+3\cdot (1-0)=4$.

## 4 Bias of the hazard difference estimator for the causal estimand

Now, we use analytical and simulation approaches to examine the bias of ${\hat{\delta_{2}}}^{D*}(\phi ,\text{ρ})$ relative to the causal estimand.

### 4.1 Analytic comparison

First, re-write the causal estimand $\delta_{2}^{D}(\phi ,\text{ρ})$ as follows (See derivation in eAppendix 3):

(6) $$\delta_{2}^{D}(\phi ,\text{ρ})\;=N\cdot \left\{\rho_{0}\cdot \left(\frac{\rho_{1}\text{Δ}{\bar{Y}}_{1}(1;\text{ρ})+\rho_{2}\text{Δ}{\bar{Y}}_{1}(2;\text{ρ})}{\rho_{1}+\rho_{2}}-\text{Δ}{\bar{Y}}_{1}(0;\text{ρ})\right)\right.\;\left.\;+\left[\left(\rho_{0}+\rho_{1}\right)\cdot \text{Δ}{\bar{Y}}_{2}(2;\text{ρ})-\left(\rho_{0}\text{Δ}{\bar{Y}}_{2}(0;\text{ρ})+\rho_{1}\text{Δ}{\bar{Y}}_{2}(1;\text{ρ})\right)\right]\right\}$$

Then, expand $E\left[{\hat{\delta_{2}}}^{D*}(\phi ,\text{ρ})\right]$ as follows (See derivation in equation [S13] eAppendix 6):

(7) $$\begin{array}{l} E\left[{\hat{\delta }}_{2}^{D*}(\phi ,\text{ρ})\right]=E\left[{\hat{N}}_{0}^{v}(\text{ρ})\left({\hat{h}}_{1}^{u}(\text{ρ})-{\hat{h}}_{1}^{v}(\text{ρ})\right)+{\hat{N}}_{1}^{v}(\text{ρ})\left({\hat{h}}_{2}^{u}(\text{ρ})-{\hat{h}}_{2}^{v}(\text{ρ})\right)\right]\\ =N\left\{\rho_{0}\cdot \left(\frac{\rho_{1}\text{Δ}{\bar{Y}}_{1}(1;\text{ρ})+\rho_{2}\text{Δ}{\bar{Y}}_{1}(2;\text{ρ})}{\rho_{1}+\rho_{2}}-\text{Δ}{\bar{Y}}_{1}(0;\text{ρ})\right)\right.\\ \;+E\left[\left(\rho_{0}+\rho_{1}\right)\cdot \frac{\rho_{0}\left(1-{\hat{Y}}_{1}(0;\text{ρ})\right)+\rho_{1}\left(1-{\hat{Y}}_{1}(1;\text{ρ})\right)}{\left(\rho_{0}+\rho_{1}\right)\left(1-{\hat{Y}}_{1}(2;\text{ρ})\right)}\cdot \text{Δ}{\hat{Y}}_{2}(2;\text{ρ})\right]\\ \left.\;-\left(\rho_{0}\text{Δ}{\bar{Y}}_{2}(0;\text{ρ})+\rho_{1}\text{Δ}{\bar{Y}}_{2}(1;\text{ρ})\right)\right\}. \end{array}$$

If $\frac{\rho_{0}\left(1-{\hat{Y}}_{1}(0;\text{ρ})\right)+\rho_{1}\left(1-{\hat{Y}}_{1}(1;\text{ρ})\right)}{\left(\rho_{0}+\rho_{1}\right)\left(1-{\hat{Y}}_{1}(2;\text{ρ})\right)}=1$, then equation (7) equals (6) (See proof in eAppendix 6). That is, ${\hat{\delta_{2}}}^{D*}(\phi ,\text{ρ})$ is an unbiased estimator under the null (i.e., when the survival is the same between those assigned to no vaccination and those assigned with x=0 or 1). However, if $\frac{\rho_{0}\left(1-{\hat{Y}}_{1}(0;\text{ρ})\right)+\rho_{1}\left(1-{\hat{Y}}_{1}(1;\text{ρ})\right)}{\left(\rho_{0}+\rho_{1}\right)\left(1-{\hat{Y}}_{1}(2;\text{ρ})\right)}\neq 1$, then ${\hat{\delta_{2}}}^{D*}(\phi ,\text{ρ})$ is biased relative to the causal estimand, implying that it is biased if vaccination has a non-null effect.

eAppendix 7 provides an alternative unbiased estimator for quantifying directly averted outcomes with a similar (but not identical) expression and have same data requirement as ${\hat{\delta_{2}}}^{D*}(\phi ,\text{ρ})$. The alternative unbiased estimator allows estimation of directly averted deaths using data aggregated by vaccination status.

### 4.2 Simulation comparison

#### 4.2.1 Scenarios

We simulate an epidemic with strategy ${\text{ρ}}^{\prime }=\{2,60;0.2,0.3\}$ under different infection-fatality rate (IFR), vaccine efficacy against infection (VE_inf_), or vaccine efficacy against death given infection (VE_death_). We examine the bias of the hazard difference estimator ${\hat{\delta_{2}}}^{D*}\left(\phi ,{\text{ρ}}^{\prime }\right)$ relative to the causal estimand $\delta_{2}^{D}\left(\phi ,{\text{ρ}}^{\prime }\right)$, where ϕ={2,60;0}, and identify the conditions under which the bias would be substantial (Table 1).

In the main text, we explore scenarios with varying IFR, VE_inf_, and VE_death_, including several extreme scenarios for illustrative purposes. For sensitivity analyses, we explore scenarios with varying number of effective contacts (β), as well as more realistic parameter values for β, IFR, VE_inf_, and VE_death_, specifically corresponding to seasonal flu, measles, and COVID-19 (wild-type strain).

#### 4.2.2 Model

Consider a hypothetical RCT with strategy ${\text{ρ}}^{\prime }=\{2,60;0.2,0.3\}$, in which interval 0 spans Days 0–59, interval 1 spans Days 60–119, and interval 2 is the post-follow-up period on or after Day 120, such that 20% of individuals can be assigned to vaccination at Day 0, 30% to vaccination at Day 60, or the rest remain unvaccinated throughout. In the susceptible-infected-recovered-death (SIRD) model, individuals are stratified by time of vaccination. Within each stratum, we specify a continuous-time SIRD model. The subscript 0 represents those assigned to receive vaccination in the beginning of Day 0, 1 for those assigned to receive vaccination in the beginning of Day 60, and 2 for the never vaccinated. For those receiving vaccination in the beginning of Day 60, vaccine efficacies against infection $\left(\theta_{1}\right)$ and death given infection $\left(\kappa_{1}\right)$ are time-varying variables that come in effect on and after Day 60 (Table S4). The SIRD model is defined in term of continuous time t as follows:

(8) $$\left.\begin{array}{l} \frac{dS_{2}(t)}{dt}=-\lambda (t)\cdot S_{2}(t)\\ \frac{dS_{1}(t)}{dt}=-\theta_{1}(t)\cdot \lambda (t)\cdot S_{1}(t)\\ \frac{dS_{0}(t)}{dt}=-\theta \cdot \lambda (t)\cdot S_{0}(t)\\ \frac{dI_{2}(t)}{dt}=\lambda (t)\cdot S_{2}(t)-\gamma \cdot I_{2}(t)\\ \frac{dI_{1}(t)}{dt}=\theta_{1}(t)\cdot \lambda (t)\cdot S_{1}(t)-\gamma \cdot I_{1}(t)\\ \frac{dI_{0}(t)}{dt}=\theta \cdot \lambda (t)\cdot S_{0}(t)-\gamma \cdot I_{0}(t)\\ \frac{dR_{2}(t)}{dt}=(1-\mu )\cdot \gamma \cdot I_{2}(t)\\ \frac{dR_{1}(t)}{dt}=\left(1-\kappa_{1}(t)\cdot \mu \right)\cdot \gamma \cdot I_{1}(t)\\ \frac{dR_{0}(t)}{dt}=(1-\kappa \cdot \mu )\cdot \gamma \cdot I_{0}(t)\\ \frac{dD_{2}(t)}{dt}=\mu \cdot \gamma \cdot I_{2}(t)\\ \frac{dD_{1}(t)}{dt}=\kappa_{1}(t)\cdot \mu \cdot \gamma \cdot I_{1}(t)\\ \frac{dD_{0}(t)}{dt}=\kappa \cdot \mu \cdot \gamma \cdot I_{0}(t) \end{array}\right\}$$

where γ = recovery rate, $\lambda (t)=\beta \cdot \frac{I_{0}(t)+I_{1}(t)+I_{2}(t)}{N(t)}$ (the hazard rate of infection), β = the number of effective contacts made by a typical infectious individual per unit time, μ = probability of death due to infection, N(t) = sum of all individuals alive at t. eAppendix 8 shows the parameters and initial values used in simulations. For simulation, the model was discretized to day time-steps. Code is available at https://github.com/katjia/vax_rollout_impact.

#### 4.2.3. Simulations

**FIGURE 2 |:**
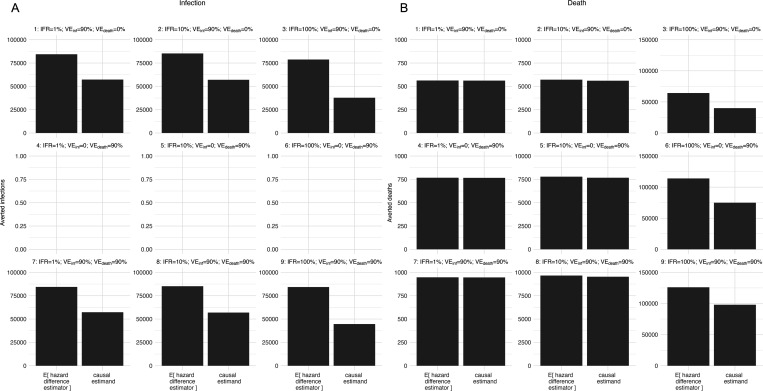
Infections (A) and deaths (B) directly averted by strategy ${\text{ρ}}^{\prime }=\{2,60;0.2,0.3\}$ under different scenarios varied by infection-fatality rate (IFR), vaccine efficacy against infection (VE_inf_), and vaccine efficacy against death given infection (VE_death_).

Figure 2 compares the expected value of hazard difference estimator (i.e., $E\left[{\hat{\delta_{2}}}^{D*}\left(\phi ,{\text{ρ}}^{\prime }\right)\right]$) with the causal estimand (i.e., $\delta_{2}^{D}\left(\phi ,{\text{ρ}}^{\prime }\right)$) under Scenarios outlined in Table 1; eAppendix 9 shows the bias of the hazard difference estimator relative to the causal estimand on the absolute and relative scales.

No infections were averted when VE_inf_ = 0% (Figure 2A; Scenarios 4 to 6). In Scenarios where VE_inf_ = 90%, the hazard difference estimator substantially overestimates the averted infections (Figure 2A; Scenarios 1 to 3, 7 to 9). This is because given the high reproduction number $\left(R_{0}\approx 3.57\right)$ and a high VE_inf_, susceptibles were preferentially depleted among the unvaccinated individuals quickly, such that by interval 1, individuals with x=2 had lower survival from infection than the average survival among individuals with x=0 and x=1 (Figure S3)—that is, $\frac{\rho_{0}^{\prime }\left(1-{\hat{Y}}_{1}\left(0;{\text{ρ}}^{\prime }\right)\right)+\rho_{1}^{\prime }\left(1-{\hat{Y}}_{1}\left(1;{\text{ρ}}^{\prime }\right)\right)}{\left(\rho_{0}^{\prime }+\rho_{1}^{\prime }\right)\left(1-{\hat{Y}}_{1}\left(2;{\text{ρ}}^{\prime }\right)\right)}>1$ in equation (7). eAppendix 11 varies the Scenarios by the number of effective contacts $\left(\beta \right)$ and show that the overestimation is more pronounced the higher β is.

The hazard difference estimator substantially overestimates the averted deaths when IFR=100%, while the overestimation is trivial when IFR≤10% (Figure 2B). This is due to the similar survival from death between vaccinated and unvaccinated individuals when IFR is low—that is, $\frac{\rho_{0}^{\prime }\left(1-{\hat{Y}}_{1}\left(0;{\text{ρ}}^{\prime }\right)\right)+\rho_{1}^{\prime }\left(1-{\hat{Y}}_{1}\left(1;{\text{ρ}}^{\prime }\right)\right)}{\left(\rho_{0}^{\prime }+\rho_{1}^{\prime }\right)\left(1-{\hat{Y}}_{1}\left(2;{\text{ρ}}^{\prime }\right)\right)}\approx 1$.

eAppendix 6 repeats the same analyses for vaccination at additional time points and over a longer period, which shows that averted infections are even more severely overestimated compared with vaccination at two time points. Consistent with the main-text finding, the hazard difference estimator sightly overestimates averted deaths when IFR≤10%. In eAppendix 12, we consider more realistic parameter values for seasonal flu, measles, and COVID-19 (wild-type strain). As with the main analyses, ${\hat{\delta_{2}}}^{D*}\left(\phi ,{\text{ρ}}^{\prime }\right)$ substantially overestimates averted infections for measles due to high basic reproduction number $\left(R_{0}=18\right)$ and high VE_inf_. It also slightly overestimates averted infections for seasonal flu and COVID-19 (wild-type), given a low $R_{0}$ (i.e., ≤2.2) and VE_inf_ > 0. The overestimation of averted deaths is trivial due to the low IFR (≤ 3% for all pathogens) (Figure S5).

## 6 Discussion

Recent empirical studies have estimated COVID-19 outcomes directly averted by vaccine rollout programs among vaccinated individuals. Here, we define a causal estimand for quantifying directly averted outcomes for vaccination at multiple time points and develop an unbiased estimator. We also examine a popular estimator used by recent empirical studies—the hazard difference estimator (as we call it)—and showed that it is biased relative to the causal estimand when vaccination has a non-null effect, as it fails to incorporate the preferential depletion of susceptibles among the unvaccinated individuals. The simulations performed here, albeit limited, suggest that the bias is substantial for averted infections, as susceptibles were preferentially depleted among the unvaccinated group quickly (due to high effective contacts and high vaccine efficacy against infection). On the other hand, the bias for averted deaths is small when IFR is ≤10% (since survival is similar between vaccinated and unvaccinated individuals), as is the case for many important infections.

Empirical studies frequently used the hazard difference estimator. As a measure, the hazard among (un)vaccinated individuals is restricted to those who have not experienced the outcome between baseline and the start of interval k for k≥1. Consequently, the interval-specific hazard is subject to differential depletion of susceptibles among the unvaccinated group over time (assuming vaccines have protective effects).^20^ However, the hazard difference estimator multiplies the number of vaccinated survivors by the hazard difference between vaccinated and unvaccinated individuals—implicitly assuming that the unvaccinated individuals have the same counterfactual survival as the vaccinated individuals, thereby failing to account for such differential depletion of susceptibles among the unvaccinated group. As our simulations show, the hazard difference estimator overestimates the number of infections averted when susceptibles are preferentially and quickly depleted among unvaccinated individuals given a high reproduction number $\left(R_{0}\approx 3.57\right)$ and a high VE_inf_. The bias is less pronounced for averted deaths when IFR is low, as survival between vaccinated and unvaccinated individuals is similar. Therefore, for COVID-19 studies using the hazard difference estimator, the averted infection estimate is likely more biased than the averted death estimate.

Researchers can use data by aggregated by vaccination status to estimate averted outcomes by following the procedures outlined in eAppendices 7 and 13. Most publicly available data from vaccine registries are aggregated by vaccination status due to privacy or other considerations. In these situations, we recommend clearly specifying the intervals to which survival and hazard pertain. An example dataset is shown in Table S11, which organizes the hazard and the number of survivors within the same row, specifying the hazard to be one interval ahead of the number of survivors. This arrangement facilitates applying formulas for the alternative unbiased estimator or hazard difference estimator. The table also includes an explanatory footnote clarifying the intervals corresponding to the hazard and the number of survivors.

In the main text, we focus on vaccine-averted outcomes. Some other empirical studies estimated vaccine-avertible deaths—deaths that could have been averted by vaccination, but were not because of a failure to vaccinate. They used the hazard difference estimator for outcomes directly *avertible* by vaccination by multiplying the hazard difference with the number of unvaccinated survivors and summing across weeks.^11,12^ eAppendix 14 defines the estimand, the unbiased estimator, and the hazard difference estimator for outcomes directly avertible, and shows simulation results under the same Scenarios as outlined in Table 1. Compared to the causal estimand, the hazard difference estimator could recover a similar value for avertible deaths when IFR is modest (≤10%) or when vaccines are highly effective at preventing death given infection (VE_death_ = 90%) (Figures S6 & S7). Note that when estimating directly avertible outcomes (or when comparing to any other counterfactual vaccination strategy than no vaccination), researchers need data disaggregated by vaccination time to use the unbiased estimator.

One major limitation is that the causal estimand and estimators proposed here are developed in the context of an ideal RCT assuming no confounding, no selection bias, perfect compliance, and no other sources of biases. In eAppendix 13, we discussed identifying the causal estimand for averted outcomes using observational data aggregated by vaccination status in the absence of confounding. In most observational settings, however, there may be strong confounding by vaccine uptake (i.e., individuals who choose to be vaccinated are also more likely to avoid infection, or elderly individuals are more likely to be vaccinated and also more likely to die from infection). In addition, individuals may reduce protective behaviors after vaccination (i.e., risk compensation),^23^ and those who have been infected are also less likely to receive vaccination.^22^ Confounding is also a concern in existing studies that estimate averted deaths from observational data, as they adjust for it by stratifying on only simplistic covariates (Table S1). Although observational studies violate the assumptions of an ideal RCT, addressing all these violations is beyond the scope of this paper. Future research could use observational data to emulate a target trial similar to the one described here for estimating vaccine-averted outcomes.

In conclusion, motivated by recent empirical studies estimating outcomes directly averted by vaccine rollout programs, we define a causal estimand for directly averted outcomes under interference, which is a lower bound on the total outcomes averted in the entire population when indirect effect is non-negative. We also develop an unbiased estimator in the context of a one-stage RCT and examine the bias of a popular estimator (the hazard difference estimator). The hazard difference estimator is biased relative to the causal estimand when vaccination has a non-null effect because it does not incorporate differential depletion of susceptibles among unvaccinated individuals. Our simulations, albeit limited, show that the hazard difference estimator could substantially overestimate the averted infections when the basic reproduction number and vaccine efficacy against infection are high, while the overestimation is small for averted deaths under modest IFR, which is the case for many important infections.

## Supplementary Material

Supplement 1

## Figures and Tables

**TABLE 1 |:** Scenarios for simulations, varied by infection-fatality rate, vaccine efficacy against infection (VE_inf_) and vaccine efficacy against death given infection (VE_death_)

Scenario	Infection-fatality rate	Vaccine efficacy
Scenario 1	1%	VE_inf_ = 90%; VE_death_ = 0%
Scenario 2	10%	VE_inf_ = 90%; VE_death_ = 0%
Scenario 3	100%	VE_inf_ = 90%; VE_death_ = 0%
Scenario 4	1%	VE_inf_ = 0%; VE_death_ = 90%
Scenario 5	10%	VE_inf_ = 0%; VE_death_ = 90%
Scenario 6	100%	VE_inf_ = 0%; VE_death_ = 90%
Scenario 7	1%	VE_inf_ = 90%; VE_death_ = 90%
Scenario 8	10%	VE_inf_ = 90%; VE_death_ = 90%
Scenario 9	100%	VE_inf_ = 90%; VE_death_ = 90%
