# Supplementary material for: Defining and Estimating Outcomes Directly Averted by a Vaccination Program when Rollout Occurs Over Time
Source: ArXiv. 2025 Oct 8:arXiv:2509.05508v3. Preprint. [Version 3] (PMC12632696)
Supplement: Supplement 1 [file NIHPP2509.05508v3-supplement-1.pdf]

eAppendix of "Defining and Estimating Outcomes Directly Averted by a Vaccination Program when Rollout Occurs Over Time"

Table of Contents

eAppendix 1. Literature review of studies estimating the number of COVID-19 outcomes directly averted among vaccinated individuals or the total number of outcomes averted among both vaccinated and unvaccinated individuals 3

eAppendix 2. Defining individual, group, and population average potential outcomes......... 7

eAppendix 3. Causal estimand for outcomes directly averted by vaccination 9

eAppendix 4 Conditions under which directly averted outcomes is a lower bound on overall averted outcomes 12

eAppendix 5. Unbiased estimator for the causal estimand in a one-stage randomized controlled trial 17

eAppendix 6. Extending the hazard difference estimator for vaccination at more than two time points 20

eAppendix 7. An alternative estimator for quantifying directly averted outcomes using RCT data aggregated by vaccination status 27

eAppendix 8. Parameters for simulations in the main text 29

eAppendix 9. Bias of the hazard difference estimator relative to the causal estimand on the absolute and relative scales 31

eAppendix 10. Disease dynamics under each scenario 32

eAppendix 11. Scenarios for simulations by varying the number of effective contacts 33

eAppendix 12. Simulations with more realistic parameter values 36

eAppendix 13. Using observational data aggregated by vaccination status for estimation . 40

eAppendix 14. Outcomes directly avertible by vaccination 43

References 55

eAppendix 1. Literature review of studies estimating the number of COVID-19 outcomes directly averted among vaccinated individuals or the total number of outcomes averted among both vaccinated and unvaccinated individuals

Recent studies estimated COVID-19 outcomes averted by vaccine rollout programs either by targeting the outcomes directly averted among vaccinated individuals[1–5] or by targeting the total outcomes averted among both vaccinated and unvaccinated individuals.[6–10] To better understand the estimation methodologies, we reviewed selected literature listed in **Table S1**.

TABLE S1 | Summary of selected studies that estimated COVID-19 outcomes averted by vaccine rollout programs

Year	Authors	Country	Title	COVID-19 Outcomes	Estimand [a]	Estimation methods
2021	Haas et al [1]	Israel	Infections, hospitalizations, and deaths averted via a nationwide vaccination campaign using the Pfizer–BioNTech BNT162b2 mRNA COVID-19 vaccine in Israel: a retrospective surveillance study	Infections, hospitalizations, severe or critical hospitalizations, deaths	Direct impact	1. For each age group and each day, calculate the number of vaccinated individuals at risk of developing the outcome by multiplying the number at risk with the proportion vaccinated. Also calculate the number of unvaccinated individuals by subtracting the numbers of vaccinated individuals from the total population at risk.<br>2. Calculate the hazard rate difference between unvaccinated and vaccinated individuals.<br>3. Estimate the daily averted outcomes by multiplying the number of vaccinated individuals at risk with the hazard rate difference.<br>4. Sum the daily averted outcomes across days within each age group, and then sum across age groups.
2024	Brault et al [2]	Chile	Direct impact of COVID-19 vaccination in Chile: averted cases, hospitalizations, ICU admissions, and deaths	Cases, hospitalizations, ICU admissions, deaths	Direct impact	1. For each age group and each week, calculate the number of vaccinated individuals at risk by multiplying the number of individuals at risk with the proportion vaccinated.<br>2. Calculate the incidence rate difference[b] between unvaccinated and vaccinated.<br>3. Estimate the weekly averted outcomes by multiplying the number of vaccinated individuals at risk with the incidence rate difference.<br>4. Sum the weekly averted outcomes across weeks and age groups.

2023	Santos et al [3]	Brazil	Estimated COVID-19 severe cases and deaths averted in the first year of the vaccination campaign in Brazil: a retrospective observational study	Severe cases and deaths	Direct impact	Same as Haas et al.
2022	Kayano et al [4]	Japan	Number of averted COVID-19 cases and deaths attributable to reduced risk in vaccinated individuals in Japan	Cases and deaths	Direct impact	Similar to Brault et al, except that calculations in Steps 1 to 3 are stratified by age group, day, and sex.
2023	Kayano et al [5]	Japan (Tokyo)	Assessing the COVID-19 vaccination program during the Omicron variant (B.1.1.529) epidemic in early 2022, Tokyo	Infections	Direct impact, overall impact[c]	**Direct impact**: Similar to Brault et al<br>**Overall impact**:<br>1. Develop a transmission model based on a renewal process and calibrate the model to the cumulative incidence<br>2. Simulate the counterfactual trajectory under no vaccination by eliminating the vaccination from the fitted transmission model<br>3. Estimate the averted outcomes by taking the difference between observed outcomes and that under the counterfactual scenario of no vaccination
2021	Vilches et al [6]	USA	Estimating COVID-19 infections, hospitalizations, and deaths following the US vaccination campaigns during the pandemic	Infections, hospitalizations, and deaths	Overall impact	1. Calibrate an agent-based model to incidence data<br>2. Simulate the trajectory under scenarios with and without vaccination<br>3. Estimate the averted outcomes by taking the difference in outcomes between scenarios with and without vaccination
2022	Schneider et al [7]	USA	Impact of U.S. COVID-19 Vaccination Efforts: An Update on Averted Deaths, Hospitalizations, and Health	Infections, hospitalizations, and deaths	Overall impact	Similar to Vilches et al.

			Care Costs Through March 2022			
2022	Gavish et al [8]	Israel	Population-level implications of the Israeli booster campaign to curtail COVID-19 resurgence	Cases, severe cases, and deaths	Overall impact[d]	Similar to Vilches et al., except that they considered scenarios with or without booster to investigate the outcomes averted by booster.
2021	Shoukat et al [10]	USA (New York City)	Lives saved and hospitalizations averted by COVID-19	Cases, hospitalizations, and deaths	Overall impact	1. Calibrate an agent-based model to incidence data<br>2. Simulate the trajectory under counterfactual scenario without vaccination<br>3. Estimate the averted outcomes by taking the difference in outcomes between observed outcomes and that under the counterfactual scenario of no vaccination
2022	Watson et al [9]	185 countries	Global impact of the first year of COVID-19 vaccination: a mathematical modelling study	Deaths[e]	Overall impact[f]	Similar to Vilches et al., except that they used an age-structured compartmental model

[a] Direct impact refers to the number of outcomes directly averted among vaccinated individuals; overall impact refers to the total number of outcomes averted among both vaccinated and unvaccinated individuals.

[b] Incidence rate among vaccinated individuals was implicitly conditional on not having developed the outcome until time of vaccination and thus was a hazard measure.

[c] The overall impact corresponds to what the authors referred to as the "total effect" in their paper, described as "the total effect at the population level, consisting of the vaccine-induced protection that is conferred directly and indirectly."

[d] The authors also estimated the indirect effect of boosters.

[e] The authors also estimated all-cause mortality averted.

[f] The authors also estimated the number of outcomes averted via indirect effect.

eAppendix 2. Defining individual, group, and population average potential outcomes

1 Mixed individual assignment strategy

Let $\boldsymbol{\rho} = \{q+1, d; \rho_0, \ldots, \rho_q\}$, where $\sum_{x=0}^{q+1} \rho_x = 1$, be a parameterization that governs the distribution of vaccination times $\mathbf{X}$. Let $K_x \equiv N \cdot \rho_x \equiv \sum_j I(X_j = x)$ for $x \in \{0, \ldots, q+1\}$ and $j \in \{1, \ldots, N\}$. Define $\boldsymbol{\rho}$ to be a *mixed individual assignment strategy* if K_x is fixed under $\boldsymbol{\rho}$, with $0 < K_x < N$ for all $x \in \{0, \ldots, q+1\}$ and each of the $\frac{N!}{\prod_{x=0}^{q+1}(K_x!)}$ possible individual assignments receiving equal probability.[11,12]

2 Type A parameterization with categorical treatment variable

Hudgens and Halloran[11] considered a mixed assignment strategy[12] for binary treatment variable (i.e., vaccination or non-vaccination) in defining the individual average potential outcome; their approach was referred to as Type A parameterization by VanderWeele and Tchetgen Tchetgen.[13] Here, we define the individual average potential outcomes using Type A parameterization for categorical treatment variable.

Assume $\boldsymbol{\rho}$ is a mixed assignment strategy. Let $\pi(\mathbf{X} = \mathbf{x}; \boldsymbol{\rho})$ denote the probability that the group is assigned with $\mathbf{X}$ given parameter $\boldsymbol{\rho}$. The vaccination times $\mathbf{X}$ are randomly assigned conditional on $\{K_0, \ldots, K_{q+1}\}$ with probability mass function:

$$\pi(\mathbf{X} = \mathbf{x}; \boldsymbol{\rho}) = \frac{\prod_{x=0}^{q+1} I\left(\sum_{j=1}^{N} I(X_j = x) = K_x\right)}{\frac{N!}{\prod_{x=0}^{q+1}(K_x!)}}$$

where $\frac{N!}{\prod_{x=0}^{q+1}(K_x!)}$ is the number of ways to assign exactly $K_0, \ldots, K_{q+1}$ individuals to $x = 0, \ldots, q+1$, respectively.

3 Individual and group average potential outcomes

By the beginning of interval $q + 1$, the *individual average potential outcome*[11] for individual j with time of vaccination x in the group under strategy $\boldsymbol{\rho}$ is:

$$\bar{Y}_{q+1,j}(x; \boldsymbol{\rho}) \equiv \sum_{\boldsymbol{\omega} \in \mathcal{X}(N-1)} Y_{q+1,j}\left(\mathbf{x}_{-j} = \boldsymbol{\omega}, x_j = x\right) \Pr_{\boldsymbol{\rho}}\left(\mathbf{X}_{-j} = \boldsymbol{\omega} | X_j = x\right)$$

where $\Pr_{\boldsymbol{\rho}}\left(\mathbf{X}_{-j} = \boldsymbol{\omega} | X_j = x\right) = \frac{\pi\left(\mathbf{X}_{-j}=\boldsymbol{\omega}, X_j = x; \boldsymbol{\rho}\right)}{\sum_{\boldsymbol{\omega}' \in \mathcal{X}(N-1)} \pi\left(\mathbf{X}_{-j}=\boldsymbol{\omega}', X_j = x; \boldsymbol{\rho}\right)}$. See Definition 2 in VanderWeele and Tchetgen Tchetgen for the analog with a binary treatment variable.[13] As they noted,[13] if $\bar{Y}_{q+1,j}$ is defined under Type A parameterization, the proportion of other individuals assigned to, for example, $x = 0$, varies depending on whether individual j is assigned to $x = 0$. In other words, the proportion ρ_0 for others is not held fixed in $\bar{Y}_{q+1,j}(0; \boldsymbol{\rho})$ compared to $\bar{Y}_{q+1,j}(k; \boldsymbol{\rho})$ for some $k > 0$, so that the so-called direct effect (which can be written as $DE_{q+1}((k, 0); \boldsymbol{\rho}) = \bar{Y}_{q+1}(k; \boldsymbol{\rho}) - \bar{Y}_{q+1}(0; \boldsymbol{\rho}) = \frac{\sum_{j=1}^{N}\left(\bar{Y}_{q+1,j}(k;\boldsymbol{\rho}) - \bar{Y}_{q+1,j}(0;\boldsymbol{\rho})\right)}{N}$)[11] does not merit the label "direct effect." However, this issue is negligible under a large group size N.

The group average potential outcome is:

$$\bar{Y}_{q+1}(x; \boldsymbol{\rho}) = \frac{\sum_{j=1}^{N} \bar{Y}_{q+1,j}\left(x_j = x; \boldsymbol{\rho}\right)}{N}.$$

eAppendix 3. Causal estimand for outcomes directly averted by vaccination

1 Extending the causal estimand to an arbitrary number of vaccination times

For $q \in \mathbb{N}$, the causal estimand for outcomes directly averted by $\boldsymbol{\rho} = \{q+1, d; \rho_0, \ldots, \rho_q\}$ where $\sum_{x=0}^{q+1} \rho_x = 1$, compared to no vaccination $\boldsymbol{\phi} = \{q+1, d; \mathbf{0}\}$ is:

Definition S1 (Causal estimand for directly averted outcomes)

$$\delta_{q+1}^{D}(\boldsymbol{\phi}, \boldsymbol{\rho}) = N \cdot \sum_{k=0}^{q} \rho_k \cdot \left[\bar{Y}_{q+1}(q+1; \boldsymbol{\rho}) - \bar{Y}_{q+1}(k; \boldsymbol{\rho})\right] \quad \text{(S1)}$$

$$= N \cdot \sum_{k=0}^{q} \rho_k \cdot DE_{q+1}\big((q+1, k); \boldsymbol{\rho}\big)$$

$\delta_{q+1}^{D}(\boldsymbol{\phi}, \boldsymbol{\rho})$ in equation (S1) can be re-written as:

$$\delta_{q+1}^{D}(\boldsymbol{\phi}, \boldsymbol{\rho})$$

$$= N \cdot \sum_{k=0}^{q} \rho_k \cdot \left[\sum_{l=1}^{q+1} \Delta\bar{Y}_l(q+1; \boldsymbol{\rho}) - \sum_{l=1}^{q+1} \Delta\bar{Y}_l(k; \boldsymbol{\rho})\right]$$

$$= N \cdot \left[\rho_0 \cdot \left(\sum_{l=1}^{q+1} \Delta\bar{Y}_l(q+1; \boldsymbol{\rho}) - \sum_{l=1}^{q+1} \Delta\bar{Y}_l(0; \boldsymbol{\rho})\right) + \rho_1 \cdot \left(\sum_{l=1}^{q+1} \Delta\bar{Y}_l(q+1; \boldsymbol{\rho}) - \sum_{l=1}^{q+1} \Delta\bar{Y}_l(1; \boldsymbol{\rho})\right) + \rho_2 \right.$$
$$\cdot \left(\sum_{l=1}^{q+1} \Delta\bar{Y}_l(q+1; \boldsymbol{\rho}) - \sum_{l=1}^{q+1} \Delta\bar{Y}_l(2; \boldsymbol{\rho})\right) + \cdots + \rho_h \cdot \left(\sum_{l=1}^{q+1} \Delta\bar{Y}_l(q+1; \boldsymbol{\rho}) - \sum_{l=1}^{q+1} \Delta\bar{Y}_l(h; \boldsymbol{\rho})\right) + \cdots + \rho_q$$
$$\left. \cdot \left(\sum_{l=1}^{q+1} \Delta\bar{Y}_l(q+1; \boldsymbol{\rho}) - \sum_{l=1}^{q+1} \Delta\bar{Y}_l(q; \boldsymbol{\rho})\right)\right]$$

$$= N \cdot \left[\rho_0 \cdot \left(\sum_{l=1}^{q+1} \Delta\bar{Y}_l(q+1; \boldsymbol{\rho}) - \sum_{l=1}^{q+1} \Delta\bar{Y}_l(0; \boldsymbol{\rho})\right) + \rho_1 \cdot \left(\sum_{l=2}^{q+1} \Delta\bar{Y}_l(q+1; \boldsymbol{\rho}) - \sum_{l=2}^{q+1} \Delta\bar{Y}_l(1; \boldsymbol{\rho})\right) + \rho_2 \right.$$
$$\cdot \left(\sum_{l=3}^{q+1} \Delta\bar{Y}_l(q+1; \boldsymbol{\rho}) - \sum_{l=3}^{q+1} \Delta\bar{Y}_l(2; \boldsymbol{\rho})\right) + \cdots + \rho_{h-1} \cdot \left(\sum_{l=h}^{q+1} \Delta\bar{Y}_l(q+1; \boldsymbol{\rho}) - \sum_{l=h}^{q+1} \Delta\bar{Y}_l(h-1; \boldsymbol{\rho})\right) + \cdots + \rho_q$$
$$\left. \cdot \left(\Delta\bar{Y}_{q+1}(q+1; \boldsymbol{\rho}) - \Delta\bar{Y}_{q+1}(q; \boldsymbol{\rho})\right)\right]$$

$$= N \cdot \Bigg[\rho_0 \cdot \left(\Delta\bar{Y}_1(q+1;\boldsymbol{\rho}) - \Delta\bar{Y}_1(0;\boldsymbol{\rho})\right) + (\rho_0+\rho_1) \cdot \left(\Delta\bar{Y}_2(q+1;\boldsymbol{\rho}) - \frac{\rho_0\Delta\bar{Y}_2(0;\boldsymbol{\rho}) + \rho_1\Delta\bar{Y}_2(1;\boldsymbol{\rho})}{\rho_0+\rho_1}\right) + (\rho_0+\rho_1+\rho_2)$$

$$\cdot \left(\Delta\bar{Y}_3(q+1;\boldsymbol{\rho}) - \frac{\rho_0\Delta\bar{Y}_3(0;\boldsymbol{\rho}) + \rho_1\Delta\bar{Y}_3(1;\boldsymbol{\rho}) + \rho_2\Delta\bar{Y}_3(2;\boldsymbol{\rho})}{\rho_0+\rho_1+\rho_2}\right) + \cdots + \left(\sum_{k=0}^{h-1}\rho_k\right)$$

$$\cdot \left(\Delta\bar{Y}_h(q+1;\boldsymbol{\rho}) - \frac{\sum_{k=0}^{h-1}\rho_k\Delta\bar{Y}_h(k;\boldsymbol{\rho})}{\sum_{k=0}^{h-1}\rho_k}\right) + \cdots + \left(\sum_{k=0}^{q}\rho_k\right) \cdot \left(\Delta\bar{Y}_{q+1}(q+1;\boldsymbol{\rho}) - \frac{\sum_{k=0}^{q}\rho_k\Delta\bar{Y}_{q+1}(k;\boldsymbol{\rho})}{\sum_{k=0}^{q}\rho_k}\right)\Bigg]$$

$$= N \cdot \sum_{l=1}^{q+1}\left[\left(\sum_{k=0}^{l-1}\rho_k\right) \cdot \left(\Delta\bar{Y}_l(q+1;\boldsymbol{\rho}) - \frac{\sum_{k=0}^{l-1}\rho_k\Delta\bar{Y}_l(k;\boldsymbol{\rho})}{\sum_{k=0}^{l-1}\rho_k}\right)\right]. \qquad \text{(S2)}$$

We assume individuals with $x = l < q+1$ have had the same historical probability of developing the outcome as those with $x = q+1$, up until l. Therefore, from the third to the forth line, $\Delta\bar{Y}_1(q+1;\boldsymbol{\rho})$ cancels out $\Delta\bar{Y}_1(1;\boldsymbol{\rho})$, since $x = 1$ is unvaccinated before the beginning of interval 1; similarly, $\sum_{l=1}^{2}\Delta\bar{Y}_l(q+1;\boldsymbol{\rho})$ cancels out $\sum_{l=1}^{2}\Delta\bar{Y}_l(2;\boldsymbol{\rho})$, since $x = 2$ is unvaccinated before the beginning of interval 2, and so on for other vaccination groups. In words, equation (S2) is the proportion ever-vaccinated (i.e., $\sum_{k=0}^{l-1}\rho_k$) multiplied with the difference between the period incidence $\Delta\bar{Y}_l(\cdot)$ among individuals assigned to no vaccination and the average period incidence among individuals ever-vaccinated, summed across time intervals $l \in (1, \ldots, q+1)$.

Furthermore, as we assume individuals with $x = l < q+1$ have had the same historical probability of developing outcome as those with $x = q+1$, up until l, we have $\Delta\bar{Y}_l(q+1;\boldsymbol{\rho}) = \frac{\sum_{k=l}^{q+1}\rho_k\Delta\bar{Y}_l(k;\boldsymbol{\rho})}{\sum_{k=l}^{q+1}\rho_k}$, such that equation (S2) can be written as:

$$\delta_{q+1}^{D}(\boldsymbol{\phi},\boldsymbol{\rho}) = N \cdot \sum_{l=1}^{q+1}\left[\left(\sum_{k=0}^{l-1}\rho_k\right) \cdot \left(\frac{\sum_{k=l}^{q+1}\rho_k\Delta\bar{Y}_l(k;\boldsymbol{\rho})}{\sum_{k=l}^{q+1}\rho_k} - \frac{\sum_{k=0}^{l-1}\rho_k\Delta\bar{Y}_l(k;\boldsymbol{\rho})}{\sum_{k=0}^{l-1}\rho_k}\right)\right] \qquad \text{(S3)}$$

$$= N \cdot \left[\sum_{l=1}^{q+1}\left(\sum_{k=0}^{l-1}\rho_k\right) \cdot \frac{\sum_{k=l}^{q+1}\rho_k\Delta\bar{Y}_l(k;\boldsymbol{\rho})}{\sum_{k=l}^{q+1}\rho_k} - \sum_{l=1}^{q+1}\sum_{k=0}^{l-1}\rho_k\Delta\bar{Y}_l(k;\boldsymbol{\rho})\right] \qquad \text{(S4)}$$

Writing $\Delta\bar{Y}_l(q+1;\boldsymbol{\rho}) = \frac{\sum_{k=l}^{q+1}\rho_k\Delta\bar{Y}_l(k;\boldsymbol{\rho})}{\sum_{k=l}^{q+1}\rho_k}$ also has the advantage of preserving data because they are equivalent in an ideal trial while $\frac{\sum_{k=l}^{q+1}\rho_k\Delta\bar{Y}_l(k;\boldsymbol{\rho})}{\sum_{k=l}^{q+1}\rho_k}$ can be estimated with a larger sample.

2 An example: vaccination at two time points

To be explicit, consider vaccination at two time points. Recall equation (3) that defines outcomes directly averted by $\boldsymbol{\rho} = \{2, d; \rho_0, \rho_1\}$, compared to no vaccination $\boldsymbol{\phi} = \{2, d; \mathbf{0}\}$:

$$\delta_2^D(\boldsymbol{\phi}, \boldsymbol{\rho})$$

$$= N \cdot [\rho_0 \cdot \left(\bar{Y}_2(2;\boldsymbol{\rho}) - \bar{Y}_2(0;\boldsymbol{\rho})\right) + \rho_1 \cdot \left(\bar{Y}_2(2;\boldsymbol{\rho}) - \bar{Y}_2(1;\boldsymbol{\rho})\right)$$

$$= N \cdot \left\{\rho_0 \cdot \left[\left(\Delta\bar{Y}_1(2;\boldsymbol{\rho}) + \Delta\bar{Y}_2(2;\boldsymbol{\rho})\right) - \left(\Delta\bar{Y}_1(0;\boldsymbol{\rho}) + \Delta\bar{Y}_2(0;\boldsymbol{\rho})\right)\right] + \rho_1 \cdot \left[\left(\Delta\bar{Y}_1(2;\boldsymbol{\rho}) + \Delta\bar{Y}_2(2;\boldsymbol{\rho})\right) - \left(\Delta\bar{Y}_1(1;\boldsymbol{\rho}) + \Delta\bar{Y}_2(1;\boldsymbol{\rho})\right)\right]\right\}$$

$$= N \cdot \left\{\rho_0 \cdot \left[\left(\Delta\bar{Y}_1(2;\boldsymbol{\rho}) + \Delta\bar{Y}_2(2;\boldsymbol{\rho})\right) - \left(\Delta\bar{Y}_1(0;\boldsymbol{\rho}) + \Delta\bar{Y}_2(0;\boldsymbol{\rho})\right)\right] + \rho_1 \cdot \left(\Delta\bar{Y}_2(2;\boldsymbol{\rho}) - \Delta\bar{Y}_2(1;\boldsymbol{\rho})\right)\right\}$$

$$= N \cdot \left\{\rho_0 \cdot \left(\Delta\bar{Y}_1(2;\boldsymbol{\rho}) - \Delta\bar{Y}_1(0;\boldsymbol{\rho})\right) + (\rho_0 + \rho_1)\left[\Delta\bar{Y}_2(2;\boldsymbol{\rho}) - \left(\frac{\rho_0\Delta\bar{Y}_2(0;\boldsymbol{\rho}) + \rho_1\Delta\bar{Y}_2(1;\boldsymbol{\rho})}{\rho_0 + \rho_1}\right)\right]\right\}$$

Assume $\Delta\bar{Y}_1(2;\boldsymbol{\rho}) = \frac{\rho_1\Delta\bar{Y}_1(1;\boldsymbol{\rho}) + \rho_2\Delta\bar{Y}_1(2;\boldsymbol{\rho})}{\rho_1 + \rho_2} \longrightarrow$

$$= N \cdot \left\{\rho_0 \cdot \left(\frac{\rho_1\Delta\bar{Y}_1(1;\boldsymbol{\rho}) + \rho_2\Delta\bar{Y}_1(2;\boldsymbol{\rho})}{\rho_1 + \rho_2} - \Delta\bar{Y}_1(0;\boldsymbol{\rho})\right) + \left[(\rho_0 + \rho_1) \cdot \Delta\bar{Y}_2(2;\boldsymbol{\rho}) - \left(\rho_0\Delta\bar{Y}_2(0;\boldsymbol{\rho}) + \rho_1\Delta\bar{Y}_2(1;\boldsymbol{\rho})\right)\right]\right\}$$

$$= N \cdot \sum_{l=1}^{2}\left[\left(\sum_{k=0}^{l-1}\rho_k\right) \cdot \left(\frac{\sum_{k=l}^{2}\rho_k\Delta\bar{Y}_l(k;\boldsymbol{\rho})}{\sum_{k=l}^{2}\rho_k} - \frac{\sum_{k=0}^{l-1}\rho_k\Delta\bar{Y}_l(k;\boldsymbol{\rho})}{\sum_{k=0}^{l-1}\rho_k}\right)\right]$$

$$= N \cdot \left[\sum_{l=1}^{2}\left(\sum_{k=0}^{l-1}\rho_k\right) \cdot \left(\frac{\sum_{k=l}^{2}\rho_k\Delta\bar{Y}_l(k;\boldsymbol{\rho})}{\sum_{k=l}^{2}\rho_k}\right) - \sum_{l=1}^{2}\sum_{k=0}^{l-1}\rho_k\Delta\bar{Y}_l(k;\boldsymbol{\rho})\right]$$

eAppendix 4 Conditions under which directly averted outcomes is a lower bound on overall averted outcomes

To identify conditions under which the number of directly averted outcomes among vaccinated individuals is a lower bound on the total number of outcomes averted, we first define the "overall impact" estimand to quantify outcomes averted in both vaccinated and unvaccinated individuals. Previously, we defined the overall impact estimand for vaccination at a single time point,[14] for which we now extend to multiple time points. First, we need to define the indirect, total, and overall *effects* for vaccination at multiple time points.

1 Indirect, total, and overall effects for vaccination at multiple time points

When vaccination occurs at multiple time points, there could be separate versions of indirect and total effects under each possible value $x \in \{0, \ldots, q+1\}$.

1.1 Indirect effect

In general, indirect effect can be a contrast between two strategies $\boldsymbol{\rho}$ and $\widetilde{\boldsymbol{\rho}}$, conditional on x. In notation, the indirect effect comparing probability of having developed the outcome by beginning of interval $q+1$ for an individual assigned to vaccination time x when the group follows strategy $\boldsymbol{\rho}$ versus $\widetilde{\boldsymbol{\rho}}$ is:

$$IE_{q+1}\big(x; (\boldsymbol{\rho}, \widetilde{\boldsymbol{\rho}})\big) = \bar{Y}_{q+1}(x; \boldsymbol{\rho}) - \bar{Y}_{q+1}(x; \widetilde{\boldsymbol{\rho}}). \quad \text{(S5)}$$

1.2 Total effect

Total effect can be any contrast between any combinations of (x, $\boldsymbol{\rho}$) and (x', $\widetilde{\boldsymbol{\rho}}$). In notation, the total effect comparing probability of having developed the outcome by beginning of $q+1$ for an individual assigned to vaccination time x when the group follows strategy $\boldsymbol{\rho}$ versus an individual with x' when the group follows strategy $\widetilde{\boldsymbol{\rho}}$ is:

$$TE_{q+1}\big((x, x'); (\boldsymbol{\rho}, \widetilde{\boldsymbol{\rho}})\big) = \bar{Y}_{q+1}(x; \boldsymbol{\rho}) - \bar{Y}_{q+1}(x'; \widetilde{\boldsymbol{\rho}}). \quad \text{(S6)}$$

By definition, total effect is the sum of direct and indirect effects:

$TE_{q+1}\big((x,x');(\boldsymbol{\rho},\widetilde{\boldsymbol{\rho}})\big) = \bar{Y}_{q+1}(x;\boldsymbol{\rho}) - \bar{Y}_{q+1}(x';\widetilde{\boldsymbol{\rho}})$

$= \bar{Y}_{q+1}(x;\boldsymbol{\rho}) - \bar{Y}_{q+1}(x';\boldsymbol{\rho}) + \bar{Y}_{q+1}(x';\boldsymbol{\rho}) - \bar{Y}_{q+1}(x';\widetilde{\boldsymbol{\rho}})$

$= DE_{q+1}((x,x');\boldsymbol{\rho}) + IE_{q+1}\big(x';(\boldsymbol{\rho},\widetilde{\boldsymbol{\rho}})\big).$ (S7)

Hudgens and Halloran[11] also partitioned the total effect into the sum of direct and indirect effects for vaccination at a single time point.

1.3 Overall effect

The overall effect comparing probability of having developed the outcome by beginning of $q+1$ for a typical individual in the group following strategy $\boldsymbol{\rho}$ versus $\widetilde{\boldsymbol{\rho}}$ is:

$$OE_{q+1}(\boldsymbol{\rho},\widetilde{\boldsymbol{\rho}}) = \bar{Y}_{q+1}(\boldsymbol{\rho}) - \bar{Y}_{q+1}(\widetilde{\boldsymbol{\rho}}). \quad \text{(S8)}$$

2 Partitioning overall effect when comparing no vaccination to some vaccination

Consider the comparison between no vaccination and a vaccination strategy $\boldsymbol{\rho}$, as our goal is to quantify the overall impact of the strategy. Previously, Sobel[12] and Hudgens and Halloran[11] noted that when comparing no vaccination to vaccination at baseline, overall effect is the weighted sum of total and indirect effects. Here, we extend the partitioning of overall effect to vaccination at multiple time points (Theorem S1).

Theorem S1 (Overall effect partitioning for no vaccination vs. $\boldsymbol{\rho}$*)* Let $\boldsymbol{\phi} = \{q+1, d; \mathbf{0}\}$ for $q \in \mathbb{N}$ and $d \in \mathbb{Z}^+$. Then

$$OE_{q+1}(\boldsymbol{\phi},\boldsymbol{\rho}) = \left[\sum_{k=0}^{q} \rho_k \cdot TE_{q+1}\big((q+1,k);(\boldsymbol{\phi},\boldsymbol{\rho})\big)\right] + \rho_{q+1} \cdot IE_{q+1}\big(q+1;(\boldsymbol{\phi},\boldsymbol{\rho})\big).$$

Proof. For $q \in \mathbb{N}$, we have

$OE_{q+1}(\boldsymbol{\phi},\boldsymbol{\rho}) = \bar{Y}_{q+1}(\boldsymbol{\phi}) - \bar{Y}_{q+1}(\boldsymbol{\rho})$

All is unvaccinated under $\boldsymbol{\phi} \longrightarrow$

$$= \bar{Y}_{q+1}(q+1; \boldsymbol{\phi}) - \bar{Y}_{q+1}(\boldsymbol{\rho})$$

By definition, $\sum_{k=0}^{q+1} \rho_k = 1 \longrightarrow$

$$= \left[\sum_{k=0}^{q} \rho_k \cdot \bar{Y}_{q+1}(q+1; \boldsymbol{\phi}) + \rho_{q+1} \cdot \bar{Y}_{q+1}(q+1; \boldsymbol{\phi})\right]$$

$$- \left[\sum_{k=0}^{q} \rho_k \cdot \bar{Y}_{q+1}(k; \boldsymbol{\rho}) + \rho_{q+1} \cdot \bar{Y}_{q+1}(q+1; \boldsymbol{\rho})\right]$$

$$= \sum_{k=0}^{q} \rho_k \cdot \left(\bar{Y}_{q+1}(q+1; \boldsymbol{\phi}) - \bar{Y}_{q+1}(k; \boldsymbol{\rho})\right) + \rho_{q+1} \cdot \left(\bar{Y}_{q+1}(q+1; \boldsymbol{\phi}) - \bar{Y}_{q+1}(q+1; \boldsymbol{\rho})\right)$$

$$= \sum_{k=0}^{q} \rho_k \cdot TE_{q+1}\big((q+1, k); (\boldsymbol{\phi}, \boldsymbol{\rho})\big) + \rho_{q+1} \cdot IE_{q+1}\big(q+1; (\boldsymbol{\phi}, \boldsymbol{\rho})\big)$$

Consider $q + 1 = 2$. We have $OE_2(\boldsymbol{\phi}, \boldsymbol{\rho}) = \rho_0 TE_2\big((2,0); (\boldsymbol{\phi}, \boldsymbol{\rho})\big) + \rho_1 TE_2\big((2,1); (\boldsymbol{\phi}, \boldsymbol{\rho})\big) + \rho_2 IE_2\big(2; (\boldsymbol{\phi}, \boldsymbol{\rho})\big)$. The partitioning is graphically illustrated in **Figure S1**.

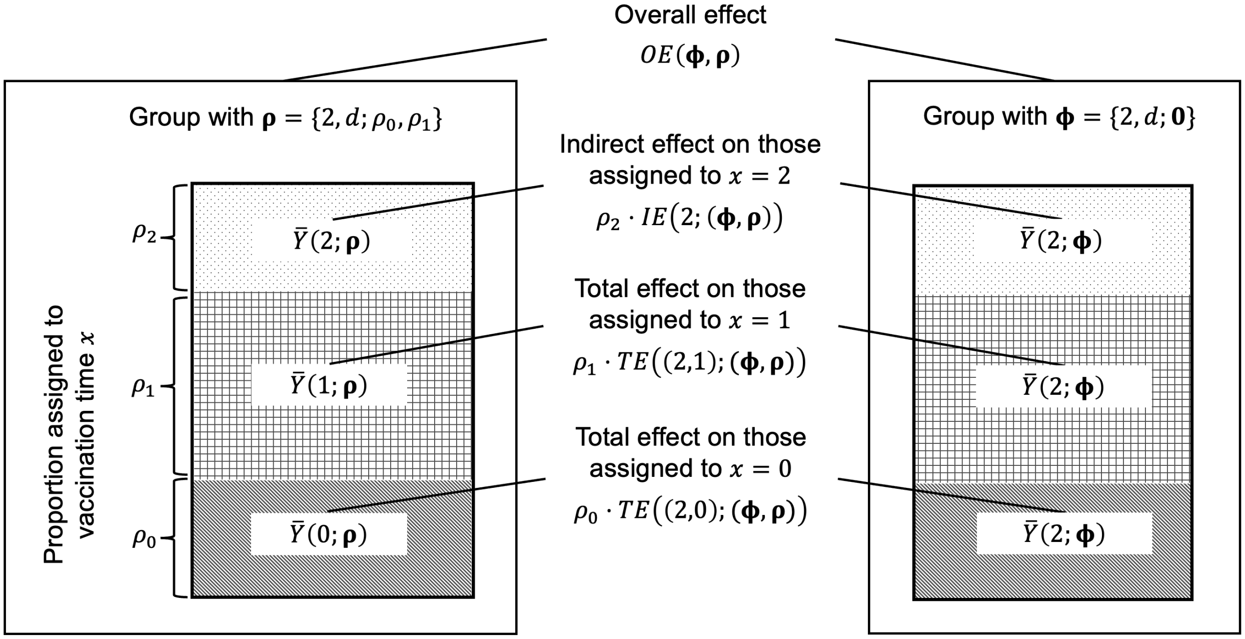

FIGURE S1 | Graphical illustration on partitioning overall effect. The two rectangles represent a pair of counterfactuals wherein the group follow strategy $\boldsymbol{\rho} = \{2, d; \rho_0, \rho_1\}$ or $\boldsymbol{\phi} = \{2, d; \mathbf{0}\}$. Individuals fall into three categories based on their assigned vaccination time: 1) The dotted region represents those who are assigned to $x = 2$ and for whom $IE(2; \boldsymbol{\phi}, \boldsymbol{\rho})$ is the difference between the counterfactuals; 2) the gridded region represents those who are assigned to $x = 1$ and for whom $TE\big((2,1); (\boldsymbol{\phi}, \boldsymbol{\rho})\big)$ is the difference between the counterfactuals; and 3) the stripped region represents those who are assigned to $x = 0$ and for whom $TE\big((2,0); (\boldsymbol{\phi}, \boldsymbol{\rho})\big)$ is the difference between the counterfactuals. Theorem S1 shows that $OE(\boldsymbol{\phi}, \boldsymbol{\rho})$ is a weighted average of three effects: 1) $IE\big(2; (\boldsymbol{\phi}, \boldsymbol{\rho})\big)$, 2) $TE\big((2,1); (\boldsymbol{\phi}, \boldsymbol{\rho})\big)$, and 3) $TE\big((2,0); (\boldsymbol{\phi}, \boldsymbol{\rho})\big)$, each weighted by the proportion of individuals for whom the effect is in operation respectively: 1) ρ_2 for those assigned to $x = 2$, 2) ρ_1 for those assigned to $x = 1$, and 3) ρ_0 for those assigned to $x = 0$. The time interval notation suppressed to reduce clutter.

3 Defining and partitioning overall impact for vaccination at multiple time points

3.1 Overall impact

Now, we use the above definition of overall effect to define the overall impact estimand and apply Theorem S1 to partition overall impact into components of direct and indirect effects.

The total number of outcomes averted among both vaccinated and unvaccinated individuals by strategy $\boldsymbol{\rho} = \{q + 1, d; \rho_0, \dots, \rho_q\}$, compared to no vaccination $\boldsymbol{\phi} = \{q + 1, d; \mathbf{0}\}$, is:

Definition S2 (Overall impact estimand for quantifying the total number of outcomes averted)

$$\delta^{O}_{q+1}(\boldsymbol{\phi}, \boldsymbol{\rho}) = N \cdot OE_{q+1}(\boldsymbol{\phi}, \boldsymbol{\rho}).$$

$\delta^{O}_{q+1}(\boldsymbol{\phi}, \boldsymbol{\rho})$ addresses the causal question: how many outcomes have been averted among both vaccinated and unvaccinated individuals under strategy $\boldsymbol{\rho}$ compared to no vaccination $\boldsymbol{\phi}$? The mathematical modeling studies[5–10] listed in **Table S1** have targeted $\delta^{O}_{q+1}(\boldsymbol{\phi}, \boldsymbol{\rho})$ to estimate total

outcomes averted by simulating the epidemic trajectory under no vaccination ($\boldsymbol{\phi}$) and comparing it with the trajectory under a particular strategy $\boldsymbol{\rho}$ (or with the observed outcomes).

3.2 Overall impact partitioning

By Theorem S1 and the definition from equation (S7) $TE_{q+1}\big((q+1,k);(\boldsymbol{\phi},\boldsymbol{\rho})\big) = IE_{q+1}\big(q+1;(\boldsymbol{\phi},\boldsymbol{\rho})\big) + DE_{q+1}\big((q+1,k);\boldsymbol{\rho}\big)$ for $k \in \{0,\dots,q\}$, we decompose $\delta^{O}_{q+1}(\boldsymbol{\phi},\boldsymbol{\rho})$:

$$\delta^{O}_{q+1}(\boldsymbol{\phi},\boldsymbol{\rho}) = N \cdot OE_{q+1}(\boldsymbol{\phi},\boldsymbol{\rho})$$

$$= N\left[\sum_{k=0}^{q} \rho_k \cdot TE_{q+1}\big((q+1,k);(\boldsymbol{\phi},\boldsymbol{\rho})\big) + \rho_{q+1} \cdot IE_{q+1}\big(q+1;(\boldsymbol{\phi},\boldsymbol{\rho})\big)\right]$$

$$= N\left[\sum_{k=0}^{q} \rho_k \cdot \Big(DE_{q+1}\big((q+1,k);\boldsymbol{\rho}\big) + IE_{q+1}\big(q+1;(\boldsymbol{\phi},\boldsymbol{\rho})\big)\Big) + \rho_{q+1} \cdot IE_{q+1}\big(q+1;(\boldsymbol{\phi},\boldsymbol{\rho})\big)\right]$$

$$= N\left[\left(\sum_{k=0}^{q} \rho_k \cdot DE_{q+1}\big((q+1,k);\boldsymbol{\rho}\big)\right) + IE_{q+1}\big(q+1;(\boldsymbol{\phi},\boldsymbol{\rho})\big)\right] \qquad \text{(S9)}$$

Substituting the definition $\delta^{D}_{q+1}(\boldsymbol{\phi},\boldsymbol{\rho}) = N \cdot \sum_{k=0}^{q} \rho_k \cdot DE_{q+1}\big((q+1,k);\boldsymbol{\rho}\big)$ into the last line of equation (S9), we have:

$$\delta^{O}_{q+1}(\boldsymbol{\phi},\boldsymbol{\rho}) = \delta^{D}_{q+1}(\boldsymbol{\phi},\boldsymbol{\rho}) + N \cdot IE_{q+1}\big(q+1;(\boldsymbol{\phi},\boldsymbol{\rho})\big)$$

Therefore, $\delta^{O}_{q+1}(\boldsymbol{\phi},\boldsymbol{\rho}) \geq \delta^{D}_{q+1}(\boldsymbol{\phi},\boldsymbol{\rho})$ if and only if $IE_{q+1}\big(q+1;(\boldsymbol{\phi},\boldsymbol{\rho})\big) \geq 0$.

eAppendix 5. Unbiased estimator for the causal estimand in a one-stage randomized controlled trial

Recall equation (S1): $\delta^D_{q+1}(\boldsymbol{\phi}, \boldsymbol{\rho}) = N \cdot \sum_{k=0}^{q} \rho_k \cdot \left[\bar{Y}_{q+1}(q+1; \boldsymbol{\rho}) - \bar{Y}_{q+1}(k; \boldsymbol{\rho})\right]$. To identify $\delta^D_{q+1}(\boldsymbol{\phi}, \boldsymbol{\rho})$, consider the following assumptions.

Assumption 1. $\boldsymbol{\rho}$ is a mixed assignment strategy.

Recall the definition of mixed assignment strategy in **eAppendix 2**: $\boldsymbol{\rho}$ is a *mixed individual assignment strategy* if K_x is fixed under $\boldsymbol{\rho}$, with $0 < K_x < N$ for all $x \in \{0, \dots, q+1\}$ and each of the $\frac{N!}{\prod_{x=0}^{q+1}(K_x!)}$ possible individual assignments receiving equal probability.[11,12]

Assumption 2 (consistency).[26]

$$\text{If } \mathbf{X} = \mathbf{x}, \text{ then } Y_{q+1,j}(\mathbf{x}) = Y_{q+1,j}$$

for all j.

To identify $\delta^D_{q+1}(\boldsymbol{\phi}, \boldsymbol{\rho})$, we can make use of the following theorem.

Theorem S2 (Unbiased estimator for the causal estimand in a one-stage RCT). Let $\boldsymbol{\rho} = \{q+1, d; \rho_0, \dots, \rho_q\}$, where $\sum_{x=0}^{q+1} \rho_x = 1$, and $\boldsymbol{\phi} = \{q+1, d; \mathbf{0}\}$ for $q \in \mathbb{N}$ and $d \in \mathbb{Z}^+$. Let

$$\widehat{\delta_{q+1}}^D(\boldsymbol{\phi}, \boldsymbol{\rho}) = N \cdot \sum_{k=0}^{q} \rho_k \cdot \left[\hat{Y}_{q+1}(q+1; \boldsymbol{\rho}) - \hat{Y}_{q+1}(k; \boldsymbol{\rho})\right] \qquad \text{(S10)}$$

where $\hat{Y}_{q+1}(x; \boldsymbol{\rho}) = \frac{\sum_{j=1}^{N} Y_{q+1,j}(\mathbf{X}) I[X_j = x]}{\sum_{j=1}^{N} I[X_j = x]}$ for $x \in \{0, \dots, q+1\}$. That is, $\hat{Y}_{q+1}(x; \boldsymbol{\rho})$ is the average of observed outcomes for individuals assigned with x under strategy $\boldsymbol{\rho}$. Under Assumpstions 1 and 2,

$$E\left[\widehat{\delta_{q+1}}^D(\boldsymbol{\phi}, \boldsymbol{\rho})\right] = \delta^D_{q+1}(\boldsymbol{\phi}, \boldsymbol{\rho}).$$

Proof: First, we expand LHS:

$$E\left[\widehat{\delta_{q+1}}^{D}(\boldsymbol{\phi}, \boldsymbol{\rho})\right] = E\left[N \cdot \sum_{k=0}^{q} \rho_k \cdot \left[\hat{Y}_{q+1}(q+1; \boldsymbol{\rho}) - \hat{Y}_{q+1}(k; \boldsymbol{\rho})\right]\right]$$

Linearity of expectation ⟶

$$= N \cdot \sum_{k=0}^{q} \left[\rho_k \cdot \left(E\left[\hat{Y}_{q+1}(q+1; \boldsymbol{\rho})\right] - E\left[\hat{Y}_{q+1}(k; \boldsymbol{\rho})\right]\right)\right] \qquad \text{(S11).}$$

We largely repeat the proof in A.1 of Hudgens and Halloran[11] to evaluate $E\left[\hat{Y}_{q+1}(x; \boldsymbol{\rho})\right]$ for $x \in \{0, \dots, q+1\}$. Without loss of generality, let $x = 0$.

$$E\left[\hat{Y}_{q+1}(0; \boldsymbol{\rho})\right] = E\left[\frac{\sum_{j=1}^{N} Y_{q+1,j}(\mathbf{X}) I\left[X_j = 0\right]}{\sum_{j=1}^{N} I\left[X_j = 0\right]}\right]$$

Under Assumption 1, $K_0 \equiv N \cdot \rho_0$ is fixed by design, and under Assumption 2 (Consistency), we have:

$$E\left[\hat{Y}_{q+1}(0; \boldsymbol{\rho})\right] = \frac{1}{K_0} \sum_{j=1}^{N} \sum_{\mathbf{s} \in \mathcal{X}(N)} \Pr_{\boldsymbol{\rho}}(\mathbf{X} = \mathbf{s}) Y_{q+1,j}(\mathbf{s}) I\left[x_j = 0\right].$$

Any $\mathbf{s}$ such that $x_j \neq 0$ does not contribute to the summation, so that we can write:

$$E\left[\hat{Y}_{q+1}(0; \boldsymbol{\rho})\right]$$

$$= \frac{1}{K_0} \sum_{j=1}^{N} \sum_{\boldsymbol{\omega} \in \mathcal{X}(N-1)} \Pr_{\boldsymbol{\rho}}\left(\mathbf{X}_{-j} = \boldsymbol{\omega}, X_j = 0\right) Y_{q+1,j}\left(\mathbf{x}_{-j} = \boldsymbol{\omega}, x_j = 0\right)$$

$$= \frac{1}{K_0} \sum_{j=1}^{N} \sum_{\boldsymbol{\omega} \in \mathcal{X}(N-1)} \Pr_{\boldsymbol{\rho}}\left(\mathbf{X}_{-j} = \boldsymbol{\omega} | X_j = 0\right) \Pr_{\boldsymbol{\rho}}\left(X_j = 0\right) Y_{q+1,j}\left(\mathbf{x}_{-j} = \boldsymbol{\omega}, x_j = 0\right).$$

Under Assumption 1, $\Pr_{\boldsymbol{\rho}}\left(X_j = 0\right) = \rho_0 = K_0/N$, implying

$$E\left[\hat{Y}_{q+1}(0; \boldsymbol{\rho})\right] = \frac{1}{N} \sum_{j=1}^{N} \sum_{\boldsymbol{\omega} \in \mathcal{X}(N-1)} \Pr_{\boldsymbol{\rho}}\left(\mathbf{X}_{-j} = \boldsymbol{\omega} | X_j = 0\right) Y_{q+1,j}\left(\mathbf{x}_{-j} = \boldsymbol{\omega}, x_j = 0\right)$$

Recall $\bar{Y}_{q+1,j}(0; \boldsymbol{\rho}) \equiv \sum_{\boldsymbol{\omega} \in \mathcal{X}(N-1)} \Pr_{\boldsymbol{\rho}}\left(\mathbf{X}_{-j} = \boldsymbol{\omega} | X_j = x\right) Y_{q+1,j}\left(\mathbf{x}_{-j} = \boldsymbol{\omega}, x_j = 0\right) \longrightarrow$

$= \frac{1}{N}\sum_{j=1}^{N} \bar{Y}_{q+1,j}(0; \boldsymbol{\rho})$

Recall $\bar{Y}_{q+1}(0; \boldsymbol{\rho}) = \dfrac{\sum_{j=1}^{N} \bar{Y}_{q+1,j}(0; \boldsymbol{\rho})}{N} \longrightarrow$

$= \bar{Y}_{q+1}(0; \boldsymbol{\rho})$.

Recall equation (S11):

$$E\left[\widehat{\delta_{q+1}}^{D}(\boldsymbol{\phi}, \boldsymbol{\rho})\right] = N \cdot \sum_{k=0}^{q} \left[\rho_k \cdot \left(E\left[\hat{Y}_{q+1}(q+1; \boldsymbol{\rho})\right] - E\left[\hat{Y}_{q+1}(k; \boldsymbol{\rho})\right]\right)\right]$$

Substituting from above $\longrightarrow$

$$= N \cdot \sum_{k=0}^{q} \rho_k \left[\bar{Y}_{q+1}(q+1; \boldsymbol{\rho}) - \bar{Y}_{q+1}(k; \boldsymbol{\rho})\right]$$

Recall equation (S1) $\longrightarrow$

$= \delta_{q+1}^{D}(\boldsymbol{\phi}, \boldsymbol{\rho})$

eAppendix 6. Extending the hazard difference estimator for vaccination at more than two time points

1 Hazard difference estimator for quantifying directly averted outcomes

The hazard difference estimator for strategy $\boldsymbol{\rho} = \{q+1, d; 0, \ldots, q\}$ for $q \in \mathbb{N}$ can be defined as follows:

$$\widehat{\delta_{q+1}}^{D*}(\boldsymbol{\phi}, \boldsymbol{\rho}) = \sum_{l=1}^{q+1} \left[\widehat{N}_{l-1}^{v}(\boldsymbol{\rho}) \cdot \left(\widehat{h}_l^u(\boldsymbol{\rho}) - \widehat{h}_l^v(\boldsymbol{\rho}) \right) \right]$$

where $\widehat{N}_{l-1}^{v}(\boldsymbol{\rho}) = N \sum_{k=0}^{l-1} \rho_k \left(1 - \widehat{Y}_{l-1}(k; \boldsymbol{\rho})\right)$, $\widehat{h}_l^u(\boldsymbol{\rho}) = \frac{\sum_{k=l}^{q+1} \rho_k \Delta \widehat{Y}_l(k;\boldsymbol{\rho})}{\sum_{k=l}^{q+1} \rho_k (1 - \widehat{Y}_{l-1}(k;\boldsymbol{\rho}))}$, and $\widehat{h}_l^v(\boldsymbol{\rho}) = \frac{\sum_{k=0}^{l-1} \rho_k \Delta \widehat{Y}_l(k;\boldsymbol{\rho})}{\sum_{k=0}^{l-1} \rho_k (1 - \widehat{Y}_{l-1}(k;\boldsymbol{\rho}))}$.

2 Bias of hazard difference estimator relative to the causal estimand

2.1 Analytical comparison

2.1.1 Bias of hazard difference estimator for an arbitrary number of vaccination times

To examine the bias of $\widehat{\delta_{q+1}}^{D*}(\boldsymbol{\phi}, \boldsymbol{\rho})$ relative to $\delta_{q+1}^{D}(\boldsymbol{\phi}, \boldsymbol{\rho})$, we first expand $E[\widehat{\delta_{q+1}}^{D*}(\boldsymbol{\phi}, \boldsymbol{\rho})]$ as follows:

$$E\left[\widehat{\delta_{q+1}}^{D*}(\boldsymbol{\phi}, \boldsymbol{\rho})\right] = E\left[\sum_{l=1}^{q+1} \left[\widehat{N}_{l-1}^{v}(\boldsymbol{\rho}) \cdot \left(\widehat{h}_l^u(\boldsymbol{\rho}) - \widehat{h}_l^v(\boldsymbol{\rho})\right)\right]\right]$$

$$= E\left[\sum_{l=1}^{q+1}\left[N \cdot \sum_{k=0}^{l-1} \rho_k \left(1 - \widehat{Y}_{l-1}(k;\boldsymbol{\rho})\right) \cdot \left(\frac{\sum_{k=l}^{q+1} \rho_k \Delta \widehat{Y}_l(k;\boldsymbol{\rho})}{\sum_{k=l}^{q+1} \rho_k (1-\widehat{Y}_{l-1}(k;\boldsymbol{\rho}))} - \frac{\sum_{k=0}^{l-1} \rho_k \Delta \widehat{Y}_l(k;\boldsymbol{\rho})}{\sum_{k=0}^{l-1} \rho_k (1-\widehat{Y}_{l-1}(k;\boldsymbol{\rho}))}\right)\right]\right]$$

$$= N\left\{E\left[\sum_{l=1}^{q+1}\left[\left[\sum_{k=0}^{l-1} \rho_k \left(1 - \widehat{Y}_{l-1}(k;\boldsymbol{\rho})\right)\right] \cdot \frac{\sum_{k=l}^{q+1} \rho_k \Delta \widehat{Y}_l(k;\boldsymbol{\rho})}{\sum_{k=l}^{q+1} \rho_k \left(1 - \widehat{Y}_{l-1}(k;\boldsymbol{\rho})\right)} - \sum_{k=0}^{l-1} \rho_k \Delta \widehat{Y}_l(k;\boldsymbol{\rho})\right]\right]\right\}$$

Linearity of expectation ⟶

$$= N\left\{\sum_{l=1}^{q+1} E\left[\left[\sum_{k=0}^{l-1} \rho_k \left(1 - \widehat{Y}_{l-1}(k;\boldsymbol{\rho})\right)\right] \cdot \frac{\sum_{k=l}^{q+1} \rho_k \Delta \widehat{Y}_l(k;\boldsymbol{\rho})}{\sum_{k=l}^{q+1} \rho_k \left(1 - \widehat{Y}_{l-1}(k;\boldsymbol{\rho})\right)}\right] - \sum_{l=1}^{q+1} \sum_{k=0}^{l-1} E[\rho_k \Delta \widehat{Y}_l(k;\boldsymbol{\rho})]\right\}$$

By a proof parallel with that of Theorem S2, we have:

$$E\left[\widehat{\delta_{q+1}}^{D*}(\boldsymbol{\phi},\boldsymbol{\rho})\right]=N\left\{\sum_{l=1}^{q+1}E\left[\left[\sum_{k=0}^{l-1}\rho_k\left(1-\hat{Y}_{l-1}(k;\boldsymbol{\rho})\right)\right]\cdot\frac{\sum_{k=l}^{q+1}\rho_k\Delta\hat{Y}_l(k;\boldsymbol{\rho})}{\sum_{k=l}^{q+1}\rho_k\left(1-\hat{Y}_{l-1}(k;\boldsymbol{\rho})\right)}\right]-\sum_{l=1}^{q+1}\sum_{k=0}^{l-1}\rho_k\Delta\bar{Y}_l(k;\boldsymbol{\rho})\right\}$$

Rearranging terms ⟶

$$=N\left\{\sum_{l=1}^{q+1}E\left[\left(\sum_{k=0}^{l-1}\rho_k\right)\cdot\frac{\frac{\sum_{k=0}^{l-1}\rho_k\left(1-\hat{Y}_{l-1}(k;\boldsymbol{\rho})\right)}{\sum_{k=0}^{l-1}\rho_k}}{\frac{\sum_{k=l}^{q+1}\rho_k\left(1-\hat{Y}_{l-1}(k;\boldsymbol{\rho})\right)}{\sum_{k=l}^{q+1}\rho_k}}\cdot\frac{\sum_{k=l}^{q+1}\rho_k\Delta\hat{Y}_l(k;\boldsymbol{\rho})}{\sum_{k=l}^{q+1}\rho_k}\right]-\sum_{l=1}^{q+1}\sum_{k=0}^{l-1}\rho_k\Delta\bar{Y}_l(k;\boldsymbol{\rho})\right\}\qquad\text{(S12)}$$

If $\frac{\frac{\sum_{k=0}^{l-1}\rho_k\left(1-\hat{Y}_{l-1}(k;\boldsymbol{\rho})\right)}{\sum_{k=0}^{l-1}\rho_k}}{\frac{\sum_{k=l}^{q+1}\rho_k\left(1-\hat{Y}_{l-1}(k;\boldsymbol{\rho})\right)}{\sum_{k=l}^{q+1}\rho_k}}=1$ (i.e., vaccination has no effect), then we can write equation (S12) as:

$$E\left[\widehat{\delta_{q+1}}^{D*}(\boldsymbol{\phi},\boldsymbol{\rho})\right]=N\left\{\sum_{l=1}^{q+1}E\left[\left(\sum_{k=0}^{l-1}\rho_k\right)\cdot\frac{\sum_{k=l}^{q+1}\rho_k\Delta\hat{Y}_l(k;\boldsymbol{\rho})}{\sum_{k=l}^{q+1}\rho_k}\right]-\sum_{l=1}^{q+1}\sum_{k=0}^{l-1}\rho_k\Delta\bar{Y}_l(k;\boldsymbol{\rho})\right\}$$

By a proof parallel with that of Theorem S2, we have:

$$E\left[\widehat{\delta_{q+1}}^{D*}(\boldsymbol{\phi},\boldsymbol{\rho})\right]=N\left\{\sum_{l=1}^{q+1}\left(\sum_{k=0}^{l-1}\rho_k\right)\cdot\frac{\sum_{k=l}^{q+1}\rho_k\Delta\bar{Y}_l(k;\boldsymbol{\rho})}{\sum_{k=l}^{q+1}\rho_k}-\sum_{l=1}^{q+1}\sum_{k=0}^{l-1}\rho_k\Delta\bar{Y}_l(k;\boldsymbol{\rho})\right\}$$

Recall equation[S4] ⟶

$$=\delta_{q+1}^{D}(\boldsymbol{\phi},\boldsymbol{\rho}).$$

However, if $\frac{\frac{\sum_{k=0}^{l-1}\rho_k\left(1-\hat{Y}_{l-1}(k;\boldsymbol{\rho})\right)}{\sum_{k=0}^{l-1}\rho_k}}{\frac{\sum_{k=l}^{q+1}\rho_k\left(1-\hat{Y}_{l-1}(k;\boldsymbol{\rho})\right)}{\sum_{k=l}^{q+1}\rho_k}}\neq 1$, then $E\left[\widehat{\delta_{q+1}}^{D*}(\boldsymbol{\phi},\boldsymbol{\rho})\right]\neq\delta_{q+1}^{D}(\boldsymbol{\phi},\boldsymbol{\rho})$. That is, $\widehat{\delta_{q+1}}^{D*}(\boldsymbol{\phi},\boldsymbol{\rho})$ is a biased estimator if the survival among vaccinated individuals is different from that among unvaccinated individuals. In an ideal RCT, the differential survival is due to a non-null effect of vaccination.

2.1.2 An example: vaccination at two time points

From equation (S12), we have:

$$E\left[\widehat{\delta_2}^{D*}(\boldsymbol{\phi},\boldsymbol{\rho})\right]=E\left[\hat{N}_0^{v}(\boldsymbol{\rho})\left(\hat{h}_1^{u}(\boldsymbol{\rho})-\hat{h}_1^{v}(\boldsymbol{\rho})\right)+\hat{N}_1^{v}(\boldsymbol{\rho})\left(\hat{h}_2^{u}(\boldsymbol{\rho})-\hat{h}_2^{v}(\boldsymbol{\rho})\right)\right]$$

$$= N\left\{\sum_{l=1}^{2} E\left[\left(\sum_{k=0}^{l-1}\rho_k\right)\cdot\frac{\frac{\sum_{k=0}^{l-1}\rho_k\left(1-\hat{Y}_{l-1}(k;\boldsymbol{\rho})\right)}{\sum_{k=0}^{l-1}\rho_k}}{\frac{\sum_{k=l}^{2}\rho_k\left(1-\hat{Y}_{l-1}(k;\boldsymbol{\rho})\right)}{\sum_{k=l}^{2}\rho_k}}\cdot\frac{\sum_{k=l}^{2}\rho_k\Delta\hat{Y}_l(k;\boldsymbol{\rho})}{\sum_{k=l}^{2}\rho_k}\right]-\sum_{l=1}^{2}\sum_{k=0}^{l-1}\rho_k\Delta\bar{Y}_l(k;\boldsymbol{\rho})\right\}$$

$$= N\left\{E\left[\rho_0\cdot\frac{\frac{\rho_0\left(1-\hat{Y}_0(0;\boldsymbol{\rho})\right)}{\rho_0}}{\frac{\rho_1\left(1-\hat{Y}_0(1;\boldsymbol{\rho})\right)+\rho_2\left(1-\hat{Y}_0(2;\boldsymbol{\rho})\right)}{\rho_1+\rho_2}}\cdot\frac{\rho_1\Delta\hat{Y}_1(1;\boldsymbol{\rho})+\rho_2\Delta\hat{Y}_1(2;\boldsymbol{\rho})}{\rho_1+\rho_2}\right]\right.$$

$$+E\left[(\rho_0+\rho_1)\cdot\frac{\frac{\rho_0\left(1-\hat{Y}_1(0;\boldsymbol{\rho})\right)+\rho_1\left(1-\hat{Y}_1(1;\boldsymbol{\rho})\right)}{\rho_0+\rho_1}}{\frac{\rho_2\left(1-\hat{Y}_0(2;\boldsymbol{\rho})\right)}{\rho_2}}\cdot\frac{\rho_2\Delta\hat{Y}_2(2;\boldsymbol{\rho})}{\rho_2}\right]$$

$$\left.-\left[\rho_0\Delta\bar{Y}_1(0;\boldsymbol{\rho})+\left(\rho_0\Delta\bar{Y}_2(0;\boldsymbol{\rho})+\rho_1\Delta\bar{Y}_2(1;\boldsymbol{\rho})\right)\right]\right\}$$

since $\hat{Y}_0 \equiv 0 \longrightarrow$

$$= N\left\{E\left[\rho_0\cdot\frac{\rho_1\Delta\hat{Y}_1(1;\boldsymbol{\rho})+\rho_2\Delta\hat{Y}_1(2;\boldsymbol{\rho})}{\rho_1+\rho_2}\right]+E\left[(\rho_0+\rho_1)\cdot\frac{\rho_0\left(1-\hat{Y}_1(0;\boldsymbol{\rho})\right)+\rho_1\left(1-\hat{Y}_1(1;\boldsymbol{\rho})\right)}{(\rho_0+\rho_1)\left(1-\hat{Y}_1(2;\boldsymbol{\rho})\right)}\cdot\Delta\hat{Y}_2(2;\boldsymbol{\rho})\right]\right.$$

$$\left.-\left[\rho_0\Delta\bar{Y}_1(0;\boldsymbol{\rho})+\left(\rho_0\Delta\bar{Y}_2(0;\boldsymbol{\rho})+\rho_1\Delta\bar{Y}_2(1;\boldsymbol{\rho})\right)\right]\right\}$$

By a proof parallel with that of Theorem S2, we have:

$$= N\left\{\rho_0\cdot\frac{\rho_1\Delta\bar{Y}_1(1;\boldsymbol{\rho})+\rho_2\Delta\bar{Y}_1(2;\boldsymbol{\rho})}{\rho_1+\rho_2}+E\left[(\rho_0+\rho_1)\cdot\frac{\rho_0\left(1-\hat{Y}_1(0;\boldsymbol{\rho})\right)+\rho_1\left(1-\hat{Y}_1(1;\boldsymbol{\rho})\right)}{(\rho_0+\rho_1)\left(1-\hat{Y}_1(2;\boldsymbol{\rho})\right)}\cdot\Delta\hat{Y}_2(2;\boldsymbol{\rho})\right]\right.$$

$$\left.-\left[\rho_0\Delta\bar{Y}_1(0;\boldsymbol{\rho})+\left(\rho_0\Delta\bar{Y}_2(0;\boldsymbol{\rho})+\rho_1\Delta\bar{Y}_2(1;\boldsymbol{\rho})\right)\right]\right\}$$

rearranging terms $\rightarrow$

$$= N\left\{\rho_0\cdot\left(\frac{\rho_1\Delta\bar{Y}_1(1;\boldsymbol{\rho})+\rho_2\Delta\bar{Y}_1(2;\boldsymbol{\rho})}{\rho_1+\rho_2}-\Delta\bar{Y}_1(0;\boldsymbol{\rho})\right)+E\left[(\rho_0+\rho_1)\cdot\frac{\rho_0\left(1-\hat{Y}_1(0;\boldsymbol{\rho})\right)+\rho_1\left(1-\hat{Y}_1(1;\boldsymbol{\rho})\right)}{(\rho_0+\rho_1)\left(1-\hat{Y}_1(2;\boldsymbol{\rho})\right)}\cdot\Delta\hat{Y}_2(2;\boldsymbol{\rho})\right]\right.$$

$$\left.-\left(\rho_0\Delta\bar{Y}_2(0;\boldsymbol{\rho})+\rho_1\Delta\bar{Y}_2(1;\boldsymbol{\rho})\right)\right\} \qquad \text{(S13)}$$

From the preceding section, we conclude that $E\left[\widehat{\delta_2}^{D*}(\boldsymbol{\phi},\boldsymbol{\rho})\right] \neq \delta_2^D(\boldsymbol{\phi},\boldsymbol{\rho})$ if $\frac{\rho_0(1-\hat{Y}_1(0;\boldsymbol{\rho}))+\rho_1(1-\hat{Y}_1(1;\boldsymbol{\rho}))}{(\rho_0+\rho_1)(1-\hat{Y}_1(2;\boldsymbol{\rho}))} \neq 1$ (i.e., vaccination has a non-null effect).

2.2 Simulations

Here we consider strategy $\boldsymbol{\rho}'' = \{99{,}7; \mathbf{0.01}\}$, where 1% of the individuals are randomized to vaccination at $x \in \{0,1,\ldots,98\}$, or to no vaccination throughout (i.e., $x = 99$). To illustrate the bias of hazard difference estimator relative to the causal estimand, we simulate the epidemic under the same nine scenarios as described in **Table 1**. Compartments are stratified by $x \in \{0,1,\ldots,99\}$, such that within each stratum x, we specify a continuous-time SIRD model in term of continuous time t as follows:

$$\left.\begin{aligned}
\frac{dS_x(t)}{dt} &= -\theta_x(t)\cdot\lambda(t)\cdot S_x(t)\\
\frac{dI_x(t)}{dt} &= \theta_x(t)\cdot\lambda(t)\cdot S_x(t) - \gamma\cdot I_x(t)\\
\frac{dR_x(t)}{dt} &= (1-\kappa_x(t)\cdot\mu)\cdot\gamma\cdot I_x(t)\\
\frac{dD_x(t)}{dt} &= \kappa_x(t)\cdot\mu\cdot\gamma\cdot I_x(t)
\end{aligned}\right\} \text{(S14)}$$

where $\lambda(t) = \beta\cdot\frac{\sum_{x=0}^{q+1} I_x(t)}{N(t)}$ and $N(t) =$ the sum of all individuals alive at t. For simulation, the model was discretized to day time-steps from Day 0 (i.e., the beginning of interval 0) to Day 693 (i.e., the beginning of interval 99). All model parameters are the same as **Table S4**, except that we use time-varying parameter $\theta_x(t)$ to parameterize 1-VE$_{\text{inf}}$ / 100%:

$$\theta_x(t) = \begin{cases} 1 & \text{if } t < \text{beginning of interval } x \\ \theta & \text{if } t \geq \text{beginning of interval } x \end{cases}$$

for vaccination interval x and time t, where $\theta_x(\cdot)$ can be 10% or 0%, depending on the scenario as outlined in **Table 1**. Similarly, we use time-varying parameter $\kappa_x(t)$ to parameterize 1-VE$_{\text{death}}$ / 100%:

$$\kappa_x(t) = \begin{cases} 1 & \text{if } t < \text{beginning of interval } x \\ \kappa & \text{if } t \geq \text{beginning of interval } x \end{cases}$$

for vaccination interval x and time t, where $\kappa_x(\cdot)$ can be 10% or 0%, depending on the scenario as outlined in **Table 1**.

Initial values for the simulation are described in **Table S2**.

TABLE S2 | Initial values of the simulations under strategy $\boldsymbol{\rho}'' = \{99,7; \mathbf{0.01}\}$.

Variable	Initial condition(s)	Definition
$\boldsymbol{N}(\mathbf{0})$	300,000	Number alive at baseline
$\boldsymbol{S_x}(\mathbf{0})$	$(300{,}000 - 300) * 0.01$	Number of susceptible individuals assigned to receive vaccination at the beginning of interval x
$\boldsymbol{I_x}(\mathbf{0})$	$300 * 0.01$	Number of infectious individuals assigned to receive vaccination at the beginning of interval x
$\boldsymbol{R_x}(\mathbf{0})$	0	Number of recovered individuals assigned to receive vaccination at the beginning of interval x
$\boldsymbol{D_x}(\mathbf{0})$	0	Number of individuals assigned to receive vaccination at the beginning of interval x who died due to infection

Figure S2 compares the expected value of hazard difference estimator and the causal estimand for directly averted infections and deaths under strategy $\boldsymbol{\rho}'' = \{99,7; \mathbf{0.01}\}$ before the beginning of Day 693, while **Table S3** shows the absolute and percentage biases. Consistent with the derivation above, the hazard difference estimator overestimates averted outcomes when $VE_{inf} > 0$, due to the lower survival of not-yet-vaccinated individuals compared to ever-vaccinated individuals (i.e., $\frac{\frac{\sum_{k=0}^{l-1} \rho_k''\left(1-\hat{Y}_{l-1}(k;\boldsymbol{\rho}'')\right)}{\sum_{k=0}^{l-1} \rho_k''}}{\frac{\sum_{k=l}^{q+1} \rho_k''\left(1-\hat{Y}_{l-1}(k;\boldsymbol{\rho}'')\right)}{\sum_{k=l}^{q+1} \rho_k''}} > 1$ in equation [S12]).

TABLE S3 | Absolute and percentage bias of the hazard difference estimator relative to the causal estimand (reference) for averted outcomes under strategy $\boldsymbol{\rho}''$ under scenarios varied by infection-fatality rate (IFR), vaccine efficacy against infection (VE_{inf}) and vaccine efficacy against death given infection (VE_{death}), as outlined in **Table 1**.

	**Scenario 1**	**Scenario 2**	**Scenario 3**	**Scenario 4**	**Scenario 5**	**Scenario 6**	**Scenario 7**	**Scenario 8**	**Scenario 9**
	IFR: 1%, VE_{inf} = 90%, VE_{death} = 0%	IFR: 10%, VE_{inf} = 90%, VE_{death}= 0%	IFR: 100%, VE_{inf} = 90%, VE_{death} = 0%	IFR: 1%, VE_{inf} = 0%, VE_{death} = 90%	IFR: 10%, VE_{inf} = 0%, VE_{death}= 90%	IFR: 100%, VE_{inf} = 0%, VE_{death} = 90%	IFR: 1%, VE_{inf} = 90%, VE_{death} = 90%	IFR: 10%, VE_{inf} = 90%, VE_{death} = 90%	IFR: 100%, VE_{inf} = 90%, VE_{death} = 90%
**Averted infections** [a]	25930.18 (191.06%)	27362.86 (204.35%)	77260.67 (1883.18%)	0.00	0.00	0.00	25926.36 (191.03%)	27319.99 (203.97%)	62417.26 (931.8%)
**Averted deaths** [a]	0.57 (0.42%)	60.81 (4.54%)	71795.51 (1749.97%)	0.76 (0.35%)	81.7 (3.74%)	164255.82 (763.85%)	0.83 (0.35%)	89 (3.77%)	173931.79 (769.03%)

[a] Percentage differences were calculated using values rounded to nine decimal places, to avoid uninterpretable, extremely small non-zero values (absolute value $< 10^{-9}$). All values in the table are rounded to two decimal places.

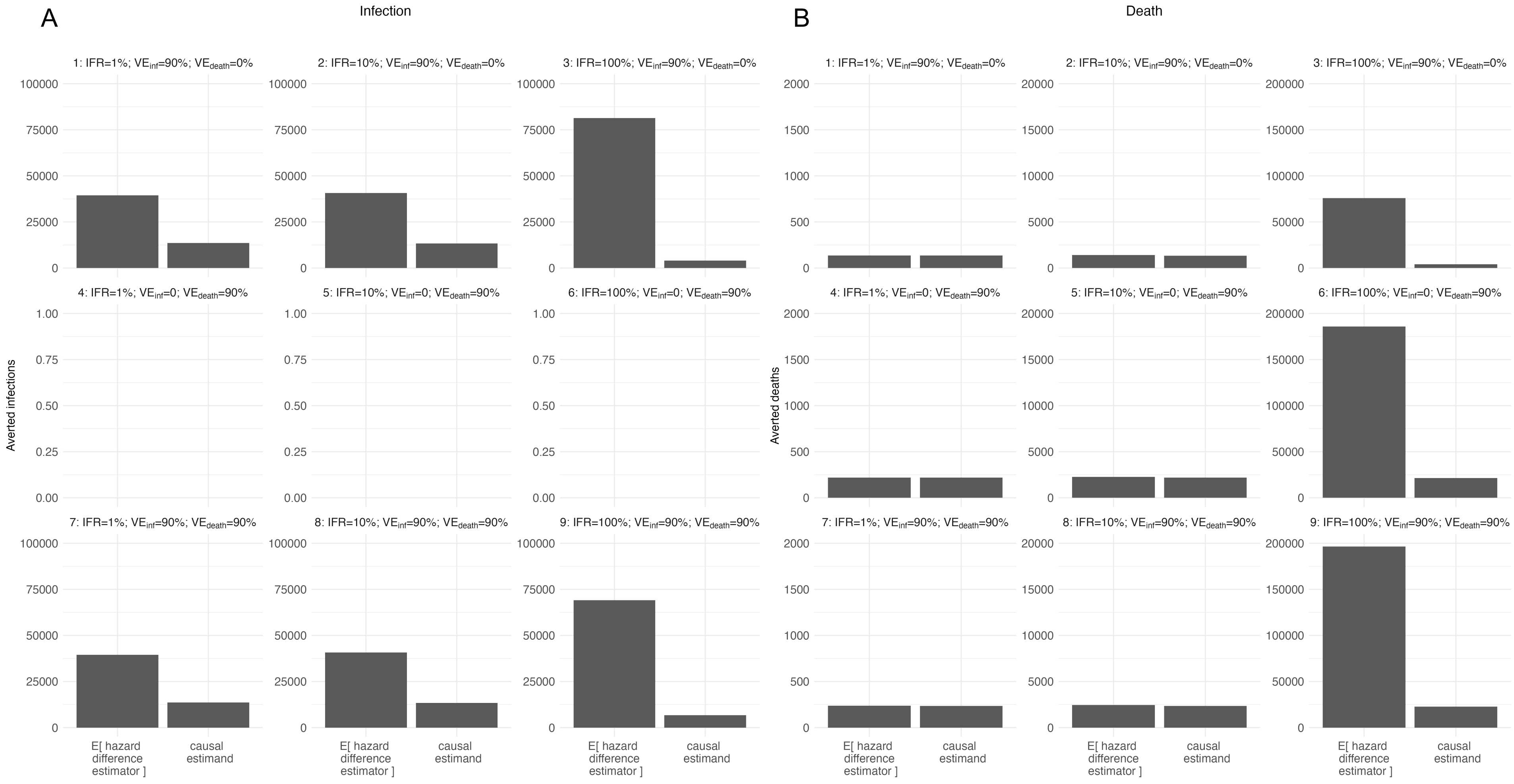

FIGURE S2 | Infections (A) and deaths (B) directly averted by strategy $\boldsymbol{\rho''} = \{99{,}7; \mathbf{0.01}\}$ under scenarios varied by infection-fatality rate (IFR), vaccine efficacy against infection (VE_{inf}) and vaccine efficacy against death given infection (VE_{death}), as outlined in **Table 1**.

eAppendix 7. An alternative estimator for quantifying directly averted outcomes using RCT data aggregated by vaccination status

Here, we show that, for averted outcomes, the causal estimand can be identified with an alternative unbiased estimator using data aggregated by vaccination status through an expression similar (but not identical) to that of the hazard difference estimator. While this estimator may appear unnecessary for RCTs, where data are disaggregated by vaccination time and equation (S10) can be directly applied, it has important implications for empirical analyses that rely on aggregated data from national vaccine systems, as discussed further in **eAppendix 13**.

Theorem S3 (Unbiasedness of an alternative estimator for averted outcomes). Let $\boldsymbol{\rho} = \{q + 1, d; \rho_0, \ldots, \rho_q\}$, where $\sum_{x=0}^{q+1} \rho_x = 1$, and $\boldsymbol{\phi} = \{q+1, d; \mathbf{0}\}$ for $q \in \mathbb{N}$ and $d \in \mathbb{Z}^+$. Let

$$\widehat{\delta_{q+1}}^{D'}(\boldsymbol{\phi}, \boldsymbol{\rho}) = \sum_{l=1}^{q+1} \left(\frac{\sum_{k=0}^{l-1} \rho_k}{\sum_{k=l}^{q+1} \rho_k} \widehat{N}_{l-1}^{u}(\boldsymbol{\rho}) \hat{h}_l^{u}(\boldsymbol{\rho}) - \widehat{N}_{l-1}^{v}(\boldsymbol{\rho}) \hat{h}_l^{v}(\boldsymbol{\rho}) \right). \qquad \text{(S15)}$$

where $\widehat{N}_{l-1}^{u}(\boldsymbol{\rho}) = N \sum_{k=l}^{q+1} \rho_k \left(1 - \hat{Y}_{l-1}(k; \boldsymbol{\rho})\right)$, $\widehat{N}_{l-1}^{v}(\boldsymbol{\rho}) = N \sum_{k=0}^{l-1} \rho_k \left(1 - \hat{Y}_{l-1}(k; \boldsymbol{\rho})\right)$, $\hat{h}_l^{u}(\boldsymbol{\rho}) = \frac{\sum_{k=l}^{q+1} \rho_k \Delta \hat{Y}_l(k;\boldsymbol{\rho})}{\sum_{k=l}^{q+1} \rho_k \left(1 - \hat{Y}_{l-1}(k;\boldsymbol{\rho})\right)}$, and $\hat{h}_l^{v}(\boldsymbol{\rho}) = \frac{\sum_{k=0}^{l-1} \rho_k \Delta \hat{Y}_l(k;\boldsymbol{\rho})}{\sum_{k=0}^{l-1} \rho_k \left(1 - \hat{Y}_{l-1}(k;\boldsymbol{\rho})\right)}$.

Under Assumptions 1 and 2 from eAppendix 5, $E\left[\widehat{\delta_{q+1}}^{D'}(\boldsymbol{\phi}, \boldsymbol{\rho})\right] = \delta_{q+1}^{D}(\boldsymbol{\phi}, \boldsymbol{\rho})$.

Proof:

$$E[\widehat{\delta_{q+1}}^{D'}(\boldsymbol{\phi}, \boldsymbol{\rho})] = E\left[\sum_{l=1}^{q+1} \left(\frac{\sum_{k=0}^{l-1} \rho_k}{\sum_{k=l}^{q+1} \rho_k} \widehat{N}_{l-1}^{u}(\boldsymbol{\rho}) \hat{h}_l^{u}(\boldsymbol{\rho}) - \widehat{N}_{l-1}^{v}(\boldsymbol{\rho}) \hat{h}_l^{v}(\boldsymbol{\rho}) \right)\right]$$

Substituting $\widehat{N}_{l-1}^{u}(\boldsymbol{\rho}), \hat{h}_l^{u}(\boldsymbol{\rho}), \widehat{N}_{l-1}^{v}(\boldsymbol{\rho})$, and $\hat{h}_l^{v}(\boldsymbol{\rho})$ from above $\longrightarrow$

$$= \sum_{l=1}^{q+1} E\left[\left(N \cdot \frac{\sum_{k=0}^{l-1} \rho_k}{\sum_{k=l}^{q+1} \rho_k} \cdot \sum_{k=l}^{q+1} \rho_k \left(1 - \hat{Y}_{l-1}(k; \boldsymbol{\rho})\right) \cdot \frac{\sum_{k=l}^{q+1} \rho_k \Delta \hat{Y}_l(k; \boldsymbol{\rho})}{\sum_{k=l}^{q+1} \rho_k \left(1 - \hat{Y}_{l-1}(k; \boldsymbol{\rho})\right)} - N \cdot \sum_{k=0}^{l-1} \rho_k \left(1 - \hat{Y}_{l-1}(k; \boldsymbol{\rho})\right) \cdot \frac{\sum_{k=0}^{l-1} \rho_k \Delta \hat{Y}_l(k; \boldsymbol{\rho})}{\sum_{k=0}^{l-1} \rho_k \left(1 - \hat{Y}_{l-1}(k; \boldsymbol{\rho})\right)} \right)\right]$$

$$= N\sum_{l=1}^{q+1}\left(E\left[\frac{\sum_{k=0}^{l-1}\rho_k}{\sum_{k=l}^{q+1}\rho_k}\cdot\sum_{k=l}^{q+1}\rho_k\Delta\hat{Y}_l(k;\boldsymbol{\rho})\right]-E\left[\sum_{k=0}^{l-1}\rho_k\Delta\hat{Y}_l(k;\boldsymbol{\rho})\right]\right).$$

By a proof parallel with that of Theorem S2, we have:

$$E[\widehat{\delta_{q+1}}^{D'}(\boldsymbol{\phi},\boldsymbol{\rho})] = N\sum_{l=1}^{q+1}\left(\frac{\sum_{k=0}^{l-1}\rho_k}{\sum_{k=l}^{q+1}\rho_k}\cdot\sum_{k=l}^{q+1}\rho_k\Delta\bar{Y}_l(k;\boldsymbol{\rho})-\sum_{k=0}^{l-1}\rho_k\Delta\bar{Y}_l(k;\boldsymbol{\rho})\right)$$

Recall equation [S4] ⟶

$$= \delta_{q+1}^{D}(\boldsymbol{\phi},\boldsymbol{\rho})$$

Note that under the null, $\frac{\sum_{k=0}^{l-1}\rho_k}{\sum_{k=l}^{q+1}\rho_k}\widehat{N}_{l-1}^{u}(\boldsymbol{\rho}) = \widehat{N}_{l-1}^{v}(\boldsymbol{\rho})$ and equation (S15) can be written as $\sum_{l=1}^{q+1}\left[\widehat{N}_{l-1}^{v}(\boldsymbol{\rho})\left(\hat{h}_l^{u}(\boldsymbol{\rho})-\hat{h}_l^{v}(\boldsymbol{\rho})\right)\right]$, which reduces to the hazard difference estimator in **eAppendix 6**. Therefore, the same type of aggregated data required by the hazard difference estimator can be used by an unbiased estimator to quantify directly averted outcomes. However, when using aggregated data to identify the causal estimand in observational studies, we recognize that further procedures are needed to estimate ρ_x for $x \in \{0,\ldots,q+1\}$ because there are no random assignments of **X**. We will further explore this in **eAppendix 13**.

eAppendix 8. Parameters for simulations in the main text

TABLE S4 | List of parameters for simulations

Parameter	Value	Definition
$\boldsymbol{\beta}$	0.25	The number of effective contacts made by a typical infectious individual per day
$\boldsymbol{\mu}$	varied	Infection-fatality rate
$\boldsymbol{\theta}$	varied	1 – vaccine efficacy against infection (i.e., VE_{inf} / 100%)
$\boldsymbol{\theta_1(t)}$	1 if t < 60 days<br>θ if $t \geq$ 60 days	Same as above
$\boldsymbol{\kappa}$	varied	1 – vaccine efficacy against death given infection (i.e., VE_{death} /100%)
$\boldsymbol{\kappa_1(t)}$	1 if t < 60 days<br>κ if $t \geq$ 60 days	Same as above
$\boldsymbol{\gamma}$	0.07	Recovery rate per day

TABLE S5 | List of initial conditions in the group under strategy $\boldsymbol{\rho}' = \{2,60; 0.2,0.3\}$

Variable	Initial condition(s)	Definition
$\boldsymbol{N(0)}$	300,000	Number alive at baseline
$\boldsymbol{S_2(0)}$	$(300{,}000 - 300) * (1 - 0.2 - 0.3)$	Number of susceptible individuals randomized to receive no vaccination
$\boldsymbol{S_1(0)}$	$(300{,}000 - 300) * 0.3$	Number of susceptible individuals randomized to receive on Day 60
$\boldsymbol{S_0(0)}$	$(300{,}000 - 300) * 0.2$	Number of susceptible individuals randomized to receive vaccination at baseline
$\boldsymbol{I_2(0)}$	$300 * (1 - 0.2 - 0.3)$	Number of infectious individuals randomized to receive no vaccination
$\boldsymbol{I_1(0)}$	$300 * 0.3$	Number of infectious individuals randomized to receive vaccination on Day 60
$\boldsymbol{I_0(0)}$	$300 * 0.2$	Number of infectious individuals randomized to receive vaccination at baseline
$\boldsymbol{R_2(0)}$	0	Number of recovered individuals randomized to receive no vaccination
$\boldsymbol{R_1(0)}$	0	Number of recovered individuals randomized to receive vaccination on Day 60

$\boldsymbol{R_0(0)}$	0	Number of recovered individuals randomized to receive vaccination at baseline
$\boldsymbol{D_2(0)}$	0	Number of individuals randomized to receive no vaccination who died due to infection
$\boldsymbol{D_1(0)}$	0	Number of individuals randomized to receive vaccination on Day 60 who died due to infection
$\boldsymbol{D_0(0)}$	0	Number of individuals randomized to receive vaccination at baseline who died due to infection

eAppendix 9. Bias of the hazard difference estimator relative to the causal estimand on the absolute and relative scales

TABLE S6 | Absolute and percentage bias of the hazard difference estimator relative to the causal estimand (reference) for averted outcomes under strategy $\boldsymbol{\rho}'$ under scenarios varied by infection-fatality rate (IFR), vaccine efficacy against infection (VE_{inf}) and vaccine efficacy against death given infection (VE_{death}), as outlined in **Table 1**.

	**Scenario 1**	**Scenario 2**	**Scenario 3**	**Scenario 4**	**Scenario 5**	**Scenario 6**	**Scenario 7**	**Scenario 8**	**Scenario 9**
	IFR: 1%, VE_{inf} = 90%, VE_{death} = 0%	IFR: 10%, VE_{inf} = 90%, VE_{death}= 0%	IFR: 100%, VE_{inf} = 90%, VE_{death} = 0%	IFR: 1%, VE_{inf} = 0%, VE_{death} = 90%	IFR: 10%, VE_{inf} = 0%, VE_{death}= 90%	IFR: 100%, VE_{inf} = 0%, VE_{death} = 90%	IFR: 1%, VE_{inf} = 90%, VE_{death} = 90%	IFR: 10%, VE_{inf} = 90%, VE_{death} = 90%	IFR: 100%, VE_{inf} = 90%, VE_{death} = 90%
**Averted infections** [a]	27139.84 (47.39%)	28279.75 (49.63%)	40903.49 (108.29%)	0.00	0.00	0.00	27127.71 (47.37%)	28151.4 (49.43%)	39707.74 (89.09%)
**Averted deaths** [a]	1.08 (0.19%)	114.18 (2.04%)	24399.47 (61.16%)	1.09 (0.14%)	116.4 (1.52%)	38964.52 (51.95%)	1.24 (0.13%)	131.14 (1.38%)	28154.09 (28.75%)

[a] Percentage differences were calculated using values rounded to nine decimal places, to avoid uninterpretable, extremely small non-zero values (absolute value $< 10^{-9}$). All values in the table are rounded to two decimal places.

eAppendix 10. Disease dynamics under each scenario

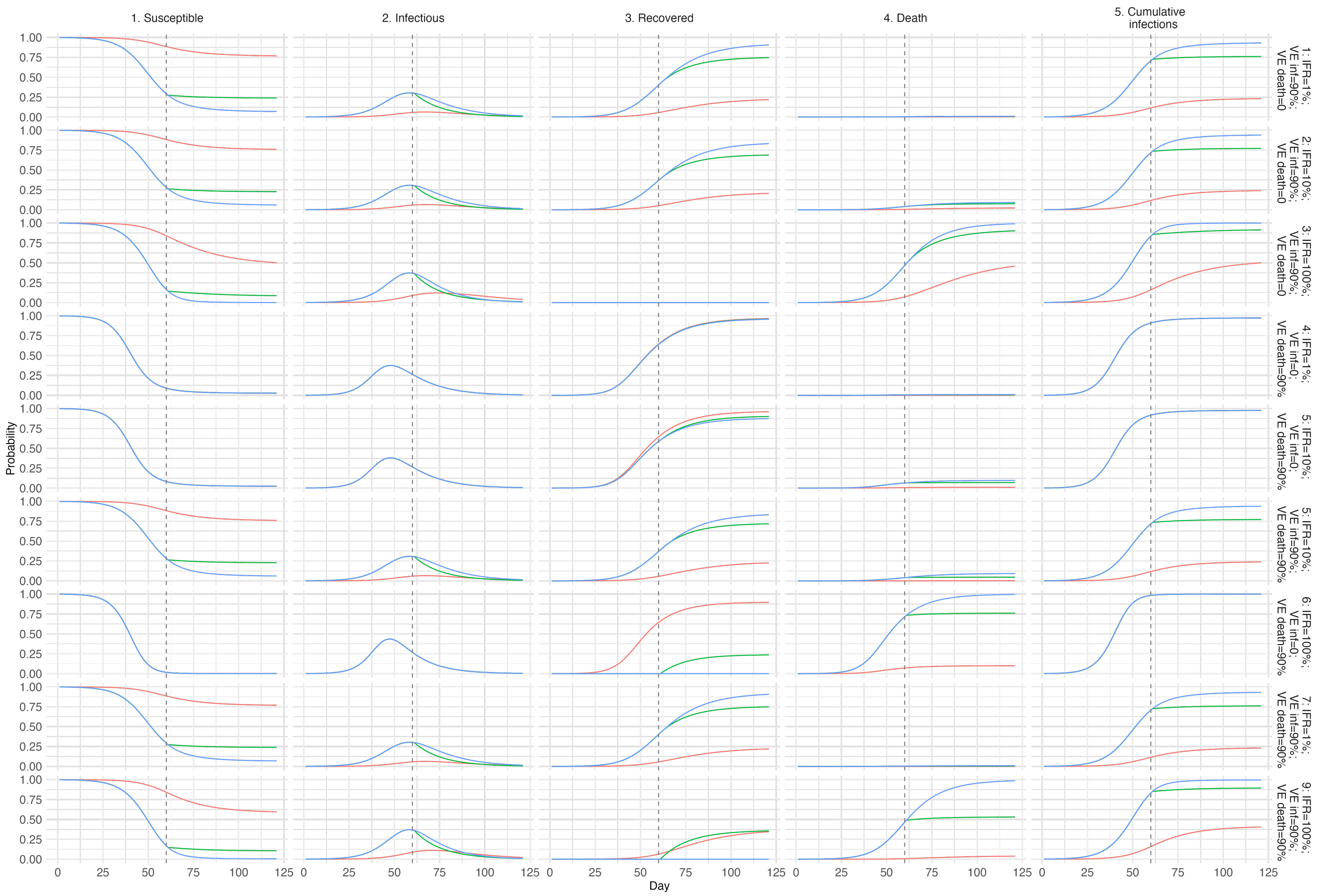

FIGURE S3 | Disease dynamics under strategy $\boldsymbol{\rho}' = \{2,60; 0.2,0.3\}$ in scenarios varied by infection-fatality rate (IFR), vaccine efficacy against infection (VE_{inf}) and vaccine efficacy against death given infection (VE_{death}). Red line represents individuals assigned to vaccination at baseline, green for those assigned to vaccination at Day 60, and blue for those remaining unvaccinated throughout. Grey dashed line indicates Day 60.

eAppendix 11. Scenarios for simulations by varying the number of effective contacts

In **Figure 2B**, we varied IFR and showed that $\widehat{\delta_2}^{D*}(\boldsymbol{\phi}, \boldsymbol{\rho}')$ substantially overestimates averted deaths when IFR = 100%, due to the stark difference in relative survival between vaccinated and not-yet-vaccinated individuals. However, when IFR ≤ 10%, the overestimation is trivial due to the similar survival across groups. Here, instead of varying IFR, we vary the number of effective contacts (β) to explore the bias. Scenarios are specified in **Table S7**. All other parameters match the main analysis (**Table S4**), except for infection-fatality rate μ, fixed at 0.1. Initial values also match the main analysis (**Table S5**).

TABLE S7 | Scenarios varied by number of effective contacts (β), vaccine efficacy against infection (VE_{inf}) and vaccine efficacy against death given infection (VE_{death})

Scenario	β	Vaccine efficacy
Scenario I	0.15	VE_{inf} = 90%; VE_{death} = 0%
Scenario II	0.2	VE_{inf} = 90%; VE_{death} = 0%
Scenario III	0.25	VE_{inf} = 90%; VE_{death} = 0%
Scenario IV	0.15	VE_{inf} = 0%; VE_{death} = 90%
Scenario V	0.2	VE_{inf} = 0%; VE_{death} = 90%
Scenario VI	0.25	VE_{inf} = 0%; VE_{death} = 90%
Scenario VII	0.15	VE_{inf} = 90%; VE_{death} = 90%
Scenario VIII	0.2	VE_{inf} = 90%; VE_{death} = 90%
Scenario IX	0.25	VE_{inf} = 90%; VE_{death} = 90%

Figure S4 compares the expected value of hazard difference estimator with the causal estimand for averted infections and deaths in Scenarios as specified in **Table S7**, while **Table S8** shows the absolute and percentage bias. As shown in **Figure S4** and **Table S8**, the hazard difference estimator overestimates averted infections in all Scenarios (except for averted infections when VE_{inf} = 0%; Scenarios IV to VI), and the bias increases with β. This is because susceptibles (from infections) are preferentially depleted among unvaccinated individuals at a faster rate when β is higher and VE_{inf} = 90%.

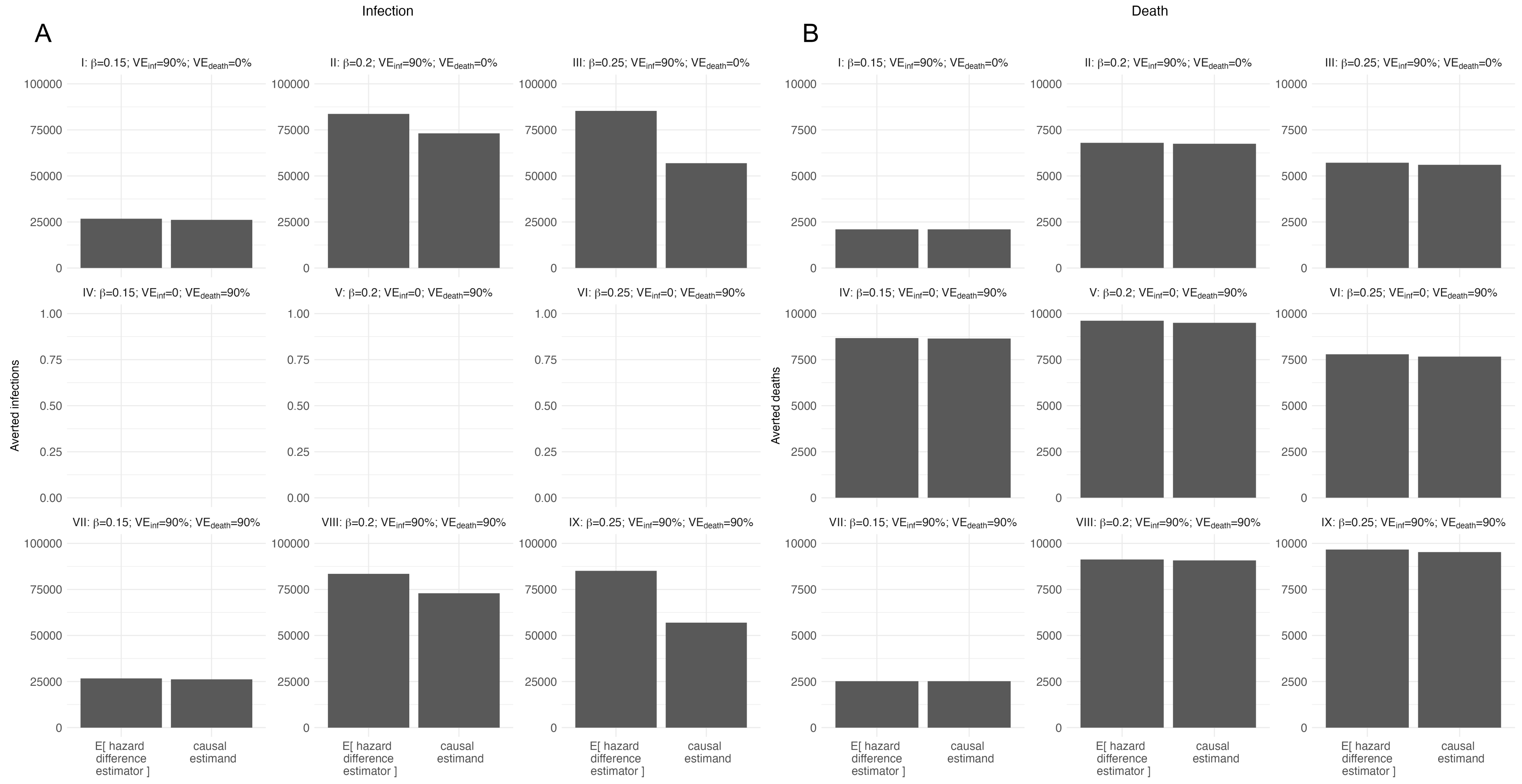

FIGURE S4 | Infections (A) and deaths (B) directly averted by strategy $\boldsymbol{\rho}' = \{2{,}60; 0.2{,}0.3\}$ among vaccinated individuals under different scenarios varied by number of effective contacts (β), vaccine efficacy against infection (VE_{inf}), and vaccine efficacy against death given infection (VE_{death}), as specified in **Table S7**

TABLE S8 | Absolute and percentage bias of the hazard difference estimator relative to the causal estimand (reference) for averted outcomes under strategy $\boldsymbol{\rho}' = \{2{,}60; 0.2{,}0.3\}$ under scenarios varied by the number of effective contacts (β), vaccine efficacy against infection (VE_{inf}) and vaccine efficacy against death given infection (VE_{death}) as specified in **Table S7**

	**Scenario I**	**Scenario II**	**Scenario III**	**Scenario IV**	**Scenario V**	**Scenario VI**	**Scenario VII**	**Scenario VIII**	**Scenario IX**
	$\beta = 0.15$, $VE_{inf} = 90\%$, $VE_{death} = 0\%$	$\beta = 0.2$, $VE_{inf} = 90\%$, $VE_{death} = 0\%$	$\beta = 0.25$, $VE_{inf} = 90\%$, $VE_{death} = 0\%$	$\beta = 0.15$, $VE_{inf} = 0\%$, $VE_{death} = 90\%$	$\beta = 0.2$, $VE_{inf} = 0\%$, $VE_{death} = 90\%$	$\beta = 0.25$, $VE_{inf} = 0\%$, $VE_{death} = 90\%$	$\beta = 0.15$, $VE_{inf} = 90\%$, $VE_{death} = 90\%$	$\beta = 0.2$, $VE_{inf} = 90\%$, $VE_{death} = 90\%$	$\beta = 0.25$, $VE_{inf} = 90\%$, $VE_{death} = 90\%$
**Averted infections** [a]	551.26 (2.1%)	10575.04 (14.47%)	28279.75 (49.63%)	0.00	0.00	0.00	550.01 (2.1%)	10526.5 (14.44%)	28151.4 (49.43%)
**Averted deaths** [a]	2.68 (0.13%)	45.64 (0.68%)	114.18 (2.04%)	25.47 (0.29%)	112.22 (1.18%)	116.4 (1.52%)	3.03 (0.12%)	50.86 (0.56%)	131.14 (1.38%)

[a] Percentage differences were calculated using values rounded to nine decimal places, to avoid uninterpretable, extremely small non-zero values (absolute value < 10^{-9}). All values in the table are rounded to two decimal places.

eAppendix 12. Simulations with more realistic parameter values

In the main text, we considered extreme combinations of IFR, VE_{inf}, and VE_{death} for illustrative purposes. Here, we consider more realistic parameter values estimated by previous studies on seasonal flu, measles, and COVID-19 (wild-type strain), as shown in **Table S9**. As in the main text, we simulate strategy $\boldsymbol{\rho}' = \{2,60; 0.2,0.3\}$, using the same model structure as in equation (8). Our objective is not to realistically model the dynamics of these epidemics—which would involve population heterogeneity, more complex infection characteristics (i.e., breakthrough infections, re-infections), waning immunity, vaccination at more time steps, and parameters that vary by time and context. Instead, we simply aim to test the robustness of our results across a broader and more realistic range of parameter values compared to those parameters used in the main text (as presented in **Table S4**).

TABLE S9 | More realistic parameter values for seasonal flu, measles, and COVID-19 (wildtype). Definitions for the parameters are provided in **Table S4**. Model equation is shown in equation (8).

Parameter	Seasonal flu	Measles	COVID-19
$\boldsymbol{\beta}$[a]	0.41 [15]	1.26 [16]	0.15 [17]
$\boldsymbol{\mu}$	3% [18]	1.3% [19]	0.11% [20]
$\boldsymbol{\theta}$	0.66 [21]	0.05 [22]	0.05 [23]
$\boldsymbol{\kappa}$	0.69 [24]	0 [25]	0.04 [26]
$\boldsymbol{\gamma}$	0.21 [15]	0.07 [27]	0.07 [28]

[a] β is obtained from applying the formula $\beta = R_0 \cdot \gamma$, where R_0 =basic reproduction number.

As **Figure S5** and **Table S10** shows, the hazard difference estimator substantially overestimates averted infections for measles due to high basic reproduction number (R_0=18) and high VE_{inf}. It also slightly overestimates averted infections for seasonal flu and COVID-19 (wild-type), given a low R_0 (i.e., R_0 ≤2.2) and $VE_{inf} > 0$. The overestimation of averted deaths is trivial due to the low IFR (≤ 3% for all pathogens).

For avertible outcomes (defined in **eAppendix 14**), the hazard difference estimator slightly underestimates avertible deaths for COVID-19. As shown later, this occurs because the hazard

difference estimator neglects the term $\rho_1 \cdot [\Delta\bar{Y}_2(1;\boldsymbol{\rho}) - \Delta\bar{Y}_2(0;\boldsymbol{\rho})]$ in the causal estimand (equation [S20]). This term is positive for COVID-19 (i.e., the period incidence of death for individuals with $x = 1$ is higher than that for individuals with $x = 0$), such that hazard difference estimator underestimates the causal estimand. For measles, there is no bias for avertible deaths under the hazard difference estimator because $\Delta\bar{Y}_2(0;\boldsymbol{\rho}) = \Delta\bar{Y}_2(1;\boldsymbol{\rho}) = 0$ (See equation [S20] and compare it with equation [S24]).

TABLE S10 | Absolute and percentage bias of the hazard difference estimator relative to the causal estimand (reference) for averted and avertible outcomes under strategy $\boldsymbol{\rho}' = (2{,}60; 0.2, 0.3)$ under more realistic parameter values for seasonal flu, measles, and COVID-19 (wild type strain)

	**Seasonal flu**	**Measles**	**COVID-19 (wild type strain)**
**Averted infections** [a]	939.98<br>(9.59%)	12133.48<br>(46.69%)	462.12<br>(1.94%)
**Averted deaths** [a]	1.02<br>(0.15%)	0.26<br>(0.03%)	0.00<br>(0.00%)
**Avertible infections** [a]	2524.04<br>(7.13%)	3153.28<br>(3.03%)	99.9<br>(0.31%)
**Avertible deaths** [a]	39.49<br>(1.63%)	0<br>(0%)	-0.16<br>(-0.53%)

[a] Percentage differences were calculated using values rounded to nine decimal places, to avoid uninterpretable, extremely small non-zero values (absolute value $< 10^{-9}$). All values in the table are rounded to two decimal places.

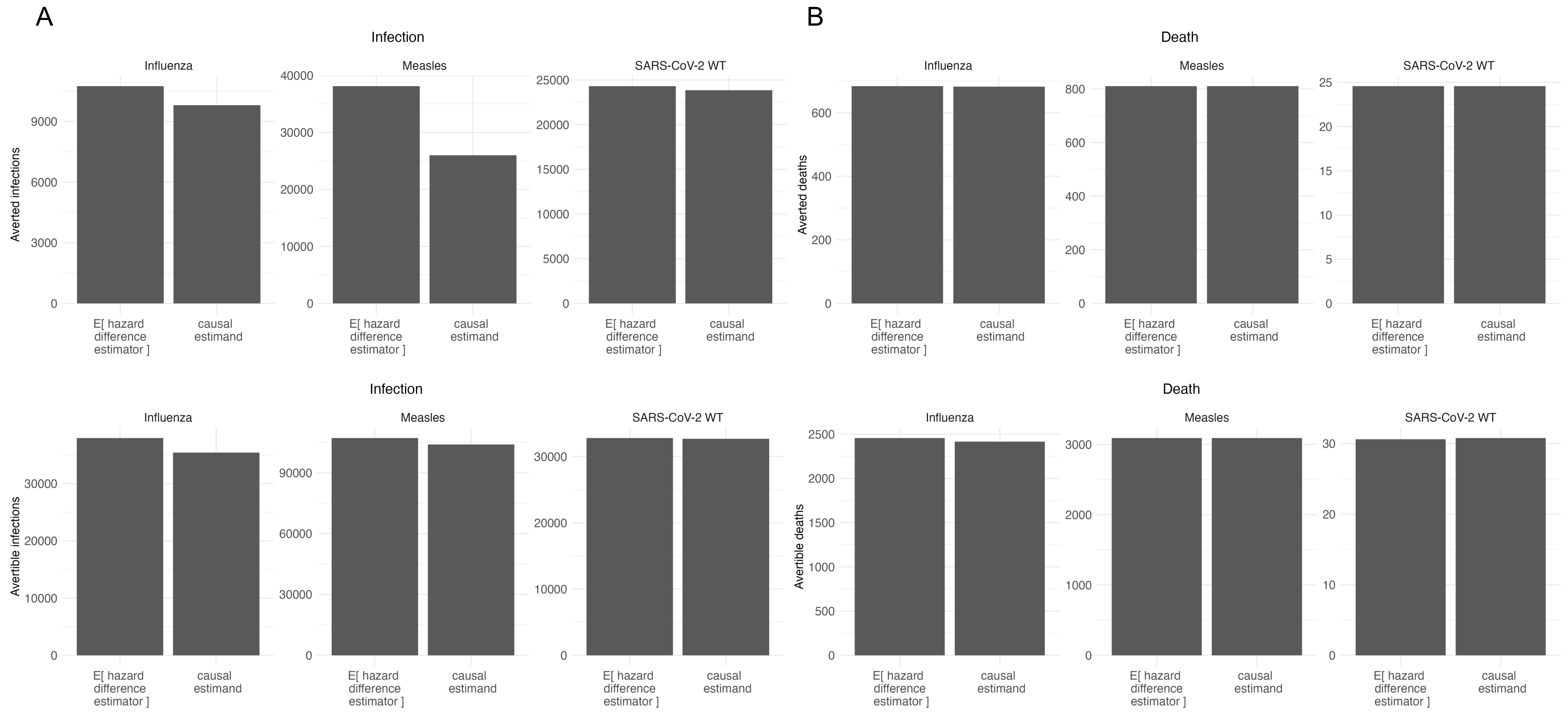

FIGURE S5 | Infections (A) and deaths (B) directly averted by strategy $\boldsymbol{\rho}' = \{2,60; 0.2,0.3\}$ among vaccinated individuals under more realistic parameter values for seasonal flu, measles, and COVID-19 (wild type strain)

eAppendix 13. Using observational data aggregated by vaccination status for estimation

eAppendix 7 defines an alternative unbiased estimator (equation [S15]) using RCT data aggregated by vaccination status. However, such an RCT is often not feasible in reality, and here we show how to identify the causal estimand using observational data aggregated by vaccination status (i.e., similar data structure as the national vaccine system[29]).

1 Obtaining $\widehat{\rho}_x$ using observational data

In the proposed RCT, we specify the strategy $\boldsymbol{\rho}$ and the proportions $\{\rho_0, \ldots, \rho_{q+1}\}$ by design. In an observational study, the investigators make no assignments, rather we only observe the time at which individuals are vaccinated (denoted as $\mathbf{X}^*$). In this case, the proportions ρ_x for $x \in \{0, \ldots, q+1\}$, must be estimated from the data. However, we cannot assume $\rho_x = E\left[\frac{\sum_{j=1}^{N} I\left[X_j^* = x^*\right]}{N}\right]$ for $x^* \in \{0, \ldots, q+1\}$ due to immortal time bias (i.e., since individuals who die before interval 1 cannot receive vaccination, individuals with $x^* = 1$ are by definition "immortal" before interval 1).[30] Here, we discuss the procedures to estimate ρ_x for $x \in \{0, \ldots, q+1\}$, addressing the immortal time bias. Note we assume the absence of confounding and additional selection bias at baseline and throughout follow-up. These assumptions are implausible in observational studies, but addressing all their violations is beyond the scope of this paper. Instead, this section focuses on identifying the causal estimand using observational data aggregated by vaccination status, addressing the immortal time bias that would otherwise exist even in the absence of confounding or additional selection bias.

Without loss of generality, consider discrete-time outcome Y for *death*. The SIRD-model in equation (S14) can be simplified into the following equation for discrete-time outcome Y:

$$N_{l+1}^{v}(\boldsymbol{\rho}) = N_{l}^{v}(\boldsymbol{\rho}) \cdot \left(1 - h_{l+1}^{v}(\boldsymbol{\rho})\right) + N \cdot \rho_{l+1} \cdot \prod_{k=0}^{l+1} \left(1 - h_{k}^{u}(\boldsymbol{\rho})\right) \quad \text{(S16)}$$

where $N_l^v(\boldsymbol{\rho}) = N\sum_{k=0}^{l}\rho_k\left(1-\bar{Y}_l(k;\boldsymbol{\rho})\right)$ is the number survived among vaccinated individuals by interval l, and $h_{l+1}^v(\boldsymbol{\rho}) = \frac{\sum_{k=0}^{l}\rho_k\Delta\bar{Y}_{l+1}(k;\boldsymbol{\rho})}{\sum_{k=0}^{l}\rho_k\left(1-\bar{Y}_l(k;\boldsymbol{\rho})\right)}$ (or $h_{l+1}^u(\boldsymbol{\rho}) = \frac{\sum_{k=l+1}^{q+1}\rho_k\Delta\bar{Y}_{l+1}(k;\boldsymbol{\rho})}{\sum_{k=l+1}^{q+1}\rho_k\left(1-\bar{Y}_l(k;\boldsymbol{\rho})\right)}$) is the hazard from interval l to $l+1$ among vaccinated (or unvaccinated) individuals.

Therefore, ρ_{l+1} can be estimated by:

$$\hat{\rho}_{l+1} = \frac{\hat{N}_{l+1}^v(\boldsymbol{\rho}) - \hat{N}_l^v(\boldsymbol{\rho})\cdot\left(1-\hat{h}_{l+1}^v(\boldsymbol{\rho})\right)}{N\cdot\prod_{k=0}^{l+1}\left(1-\hat{h}_k^u(\boldsymbol{\rho})\right)}. \quad \text{(S17)}$$

To illustrate this, **Table S11** shows a simulated dataset that is aggregated by vaccination status in a hypothetical observational study in the absence of confounding or additional selection bias. The epidemic is simulated under the (unobserved) strategy $\boldsymbol{\rho}' = \{2, d; 0.2, 0.3\}$ in Scenario 1 as described in **Table 1**.

TABLE S11 | Dataset aggregated by vaccination status in a hypothetical observational study without confounding under unobserved strategy $\boldsymbol{\rho}' = \{2,60; 0.2,0.3\}$

Interval ($\boldsymbol{k}$)	Observed number survived among vaccinated individuals ($\hat{\boldsymbol{N}}_{\boldsymbol{k}}^{\boldsymbol{v}}(\boldsymbol{\rho}')$) [a]	Hazards of death among vaccinated individuals ($\hat{\boldsymbol{h}}_{\boldsymbol{k+1}}^{\boldsymbol{v}}(\boldsymbol{\rho}')$) [b]	Observed number survived among not-yet-vaccinated individuals ($\hat{\boldsymbol{N}}_{\boldsymbol{k}}^{\boldsymbol{u}}(\boldsymbol{\rho}')$) [a]	Hazards of death among not-yet-vaccinated individuals ($\hat{\boldsymbol{h}}_{\boldsymbol{k+1}}^{\boldsymbol{u}}(\boldsymbol{\rho}')$) [b]
0	60000	0.00060716	240000	0.00426391
1	149579.818	0.0026133	149360.413	0.00491036

[a] Number of individuals survived before the start of interval k among those who are (un)vaccinated at the start of the that interval.
[b] Hazard for events occurring after the start of interval k and before the start of interval $k+1$ (i.e., hazard leads survival by one interval).

Assuming that we know the baseline vaccination proportion $\rho_0' = 0.2$, we are interested in recovering ρ_1' and ρ_2'. By substituting the data from **Table S11** into equation (S17), we have:

$$\hat{\rho}_1' = \frac{\hat{N}_1^v(\boldsymbol{\rho}') - \hat{N}_0^v(\boldsymbol{\rho}')\cdot\left(1-\hat{h}_1^v(\boldsymbol{\rho}')\right)}{\hat{N}\cdot\left(1-\hat{h}_0^u(\boldsymbol{\rho}')\right)\cdot\left(1-\hat{h}_1^u(\boldsymbol{\rho}')\right)} = \frac{149579.818 - 60000\cdot(1-0.00060716)}{300000\cdot 1\cdot(1-0.00426391)} = 0.3 = \rho'_1$$

Then, we can easily obtain $\hat{\rho}_2' = 1 - 0.2 - 0.3 = 0.5$.

3 Identifying causal estimand using observational data

Lastly, we verify that, the causal estimand can be identified using observational data aggregated by vaccination status (subject to immortal time bias until corrected by equation [S17]) in the absence of confounding or additional selection bias. By having $\boldsymbol{\rho}' = \widehat{\boldsymbol{\rho}}'$ and substituting the data from **Table S11** into equation (S15), we have:

$$\widetilde{\delta_{q+1}}^{D'}(\boldsymbol{\phi}, \boldsymbol{\rho}') = \sum_{l=1}^{2} \left(\frac{\sum_{k=0}^{l-1} \hat{\rho}'_k}{\sum_{k=l}^{q+1} \hat{\rho}'_k} \hat{N}_{l-1}^{u}(\boldsymbol{\rho}') \hat{h}_l^{u}(\boldsymbol{\rho}') - \hat{N}_{l-1}^{v}(\boldsymbol{\rho}') \hat{h}_l^{v}(\boldsymbol{\rho}') \right)$$

$$= \left[\frac{\hat{\rho}'_0}{\hat{\rho}'_1 + \hat{\rho}'_2} \hat{N}_0^{u}(\boldsymbol{\rho}') \hat{h}_1^{u}(\boldsymbol{\rho}') - \hat{N}_0^{v}(\boldsymbol{\rho}') \hat{h}_1^{v}(\boldsymbol{\rho}') \right] + \left[\frac{\hat{\rho}'_0 + \hat{\rho}'_1}{\hat{\rho}'_2} \hat{N}_1^{u}(\boldsymbol{\rho}') \hat{h}_2^{u}(\boldsymbol{\rho}') - \hat{N}_1^{v}(\boldsymbol{\rho}') \hat{h}_2^{v}(\boldsymbol{\rho}') \right]$$

$$= (\frac{0.2}{0.8} * 240000 * 0.00426391 - 60000.0 * 0.00060716) + (\frac{0.5}{0.5} * 149360.413 * 0.00491036 - 149579.818 * 0.0026133)$$

$$= 561.92146$$

Consider the simulated data from a hypothetical RCT where we have cumulative incidence data disaggregated by vaccination time (**Table S12**). By substituting the RCT data into equation (S10) (i.e., the unbiased estimator), we estimate the averted deaths to be $300000 * [0.2 * (0.00915333 - 0.00220958) + 0.3 * (0.00915333 - 0.00753893)] = 561.921$, which is the same as the result obtained from **Table S11**. Therefore, in the absence of confounding or additional selection bias, we can use observational data aggregated by vaccination status to estimate cumulative incidence difference estimand for averted outcomes.

TABLE S12 | Dataset disaggregated by vaccination time in a hypothetical one-stage RCT under strategy $\boldsymbol{\rho}' = \{2,60; 0.2,0.3\}$

Vaccination time (x)	$\boldsymbol{\rho}'_{\boldsymbol{x}}$	Cumulative incidence for death $\hat{Y}_2(x; \boldsymbol{\rho}')$
0	0.2	0.00220958
1	0.3	0.00753893
2	0.5	0.00915333

eAppendix 14. Outcomes directly avertible by vaccination

In the main text, we define the causal estimand and estimators for quantifying outcomes directly averted by vaccination. Here, we propose the causal estimand and estimators for outcomes directly *avertible* by vaccination. The proposed causal estimand considers outcomes that could have been averted under full vaccination at baseline (denoted as $\boldsymbol{\psi} = (q+1, d; 1, \mathbf{0})$ for $q \in \mathbb{N}$), but were not averted given the particular vaccination strategy $\boldsymbol{\rho}$.

1.1 Causal estimand for outcomes directly avertible by vaccination at a single time point

Our previous framework[14] defined the causal estimand for quantifying avertible outcomes for vaccination at a single time point—that is, outcomes that could have been directly averted under full vaccination at baseline $\boldsymbol{\psi} = \{1, d; 1\}$ but were not averted given the particular strategy $\boldsymbol{\rho} = \{1, d; \rho_0\}$, where $\rho_1 = 1 - \rho_0$. In notation, the estimand is:

$$\delta_1^D(\boldsymbol{\psi}, \boldsymbol{\rho}) = N \cdot \rho_1 \cdot \left(\bar{Y}_1(1; \boldsymbol{\rho}) - \bar{Y}_1(0; \boldsymbol{\rho})\right) \quad \text{(S18)}$$

1.2 Causal estimand for outcomes directly avertible by vaccination at two time points

Now, extending the estimand in equation (S18) for vaccination at two time points, we define the estimand to quantify outcomes that could have been directly averted under full vaccination at baseline $\boldsymbol{\psi} = \{2, d; 1{,}0\}$ but were not averted given the particular strategy $\boldsymbol{\rho} = \{2, d; \rho_0, \rho_1\}$. In notation, the estimand is:

$$\delta_2^D(\boldsymbol{\psi}, \boldsymbol{\rho}) = N \cdot \left[\rho_1\left(\bar{Y}_2(1; \boldsymbol{\rho}) - \bar{Y}_2(0; \boldsymbol{\rho})\right) + \rho_2\left(\bar{Y}_2(2; \boldsymbol{\rho}) - \bar{Y}_2(0; \boldsymbol{\rho})\right)\right] \quad \text{(S19)}$$

$$= N \cdot \left[\left(\rho_1 \Delta\bar{Y}_1(1; \boldsymbol{\rho}) + \rho_2 \Delta\bar{Y}_1(2; \boldsymbol{\rho})\right) - (\rho_1 + \rho_2) \cdot \Delta\bar{Y}_1(0; \boldsymbol{\rho}) + \rho_1 \cdot \left(\Delta\bar{Y}_2(1; \boldsymbol{\rho}) - \Delta\bar{Y}_2(0; \boldsymbol{\rho})\right) + \rho_2 \cdot \left(\Delta\bar{Y}_2(2; \boldsymbol{\rho}) - \Delta\bar{Y}_2(0; \boldsymbol{\rho})\right)\right]. \quad \text{(S20)}$$

In words, equation (S19) takes the difference in cumulative incidence between vaccination at baseline (i.e., $\bar{Y}_2(0; \boldsymbol{\rho})$) and vaccination at later time $x \in \{1,2\}$ (i.e., $\bar{Y}_2(1; \boldsymbol{\rho})$ or $\bar{Y}_2(2; \boldsymbol{\rho})$),

multiplied with number of individuals assigned to x=1 or 2 (i.e., $N \cdot \rho_1$ or $N \cdot \rho_2$), respectively, and sum over $x \in \{1,2\}$.

1.3 Outcomes directly avertible by vaccination at an arbitrary number of vaccination times

Extending the estimand in equation (S19) for vaccination at an arbitrary number of vaccination times, we define the estimand to quantify outcomes that could have been directly averted under full vaccination at baseline $\boldsymbol{\psi} = \{q+1, d; 1, \mathbf{0}\}$ but were not averted given the particular strategy $\boldsymbol{\rho} = \{q+1, d; \rho_0, \ldots, \rho_q\}$ for $q \in \mathbb{N}$. In notation, the estimand is:

$$\delta_{q+1}^{D}(\boldsymbol{\psi}, \boldsymbol{\rho}) = N \cdot \sum_{k=1}^{q+1} \rho_k \cdot \left(\bar{Y}_{q+1}(k; \boldsymbol{\rho}) - \bar{Y}_{q+1}(0; \boldsymbol{\rho})\right). \quad \text{(S21)}$$

2 Unbiased estimator

Similar to the unbiased estimator for directly averted outcomes in equation (S10), directly avertible outcomes (i.e., $\delta_{q+1}^{D}(\boldsymbol{\psi}, \boldsymbol{\rho})$) can be identified by:

$$\widehat{\delta_{q+1}}^{D}(\boldsymbol{\psi}, \boldsymbol{\rho}) = N \cdot \sum_{k=1}^{q+1} \rho_k \cdot \left[\hat{Y}_{q+1}(k; \boldsymbol{\rho}) - \hat{Y}_{q+1}(0; \boldsymbol{\rho})\right] \quad \text{(S22)}$$

where $\hat{Y}_{q+1}(x; \boldsymbol{\rho}) = \frac{\sum_{j=1}^{N} Y_{q+1,j}(\mathbf{X}) I[X_j = x]}{\sum_{j=1}^{N} I[X_j = x]}$ for $x \in \{0, \ldots, q+1\}$. We know that $\widehat{\delta_{q+1}}^{D}(\boldsymbol{\psi}, \boldsymbol{\rho})$ is unbiased based on a proof parallel with the proof of Theorem S2.

3 Hazard difference estimator for outcomes directly avertible by vaccination

Some empirical studies[31,32] have used the hazard difference estimator to estimate vaccine-avertible outcomes, which takes the form:

$$\widehat{\delta_{q+1}}^{D*}(\boldsymbol{\psi}, \boldsymbol{\rho}) = \sum_{l=1}^{q+1} \left[\hat{N}_{l-1}^{u}(\boldsymbol{\rho}) \cdot \left(\hat{h}_l^{u}(\boldsymbol{\rho}) - \hat{h}_l^{v}(\boldsymbol{\rho})\right)\right]$$

$$= \hat{N}_0^{u}(\boldsymbol{\rho}) \cdot \left(\hat{h}_1^{u}(\boldsymbol{\rho}) - \hat{h}_1^{v}(\boldsymbol{\rho})\right) + \sum_{l=2}^{q+1} \left[\hat{N}_{l-1}^{u}(\boldsymbol{\rho}) \cdot \left(\hat{h}_l^{u}(\boldsymbol{\rho}) - \hat{h}_l^{v}(\boldsymbol{\rho})\right)\right] \quad \text{(S23)}$$

where $\widehat{N}_{l-1}^{u}(\boldsymbol{\rho}) = N\sum_{k=l}^{q+1}\rho_k\left(1-\widehat{Y}_{l-1}(k;\boldsymbol{\rho})\right)$, $\widehat{h}_l^{u}(\boldsymbol{\rho}) = \frac{\sum_{k=l}^{q+1}\rho_k\Delta\widehat{Y}_l(k;\boldsymbol{\rho})}{\sum_{k=l}^{q+1}\rho_k\left(1-\widehat{Y}_{l-1}(k;\boldsymbol{\rho})\right)}$, and $\widehat{h}_l^{v}(\boldsymbol{\rho}) = \frac{\sum_{k=0}^{l-1}\rho_k\Delta\widehat{Y}_l(k;\boldsymbol{\rho})}{\sum_{k=0}^{l-1}\rho_k\left(1-\widehat{Y}_{l-1}(k;\boldsymbol{\rho})\right)}$. In words, the hazard difference estimator quantifies directly avertible outcomes by multiplying the hazard difference $\widehat{h}_l^{u}(\boldsymbol{\rho}) - \widehat{h}_l^{v}(\boldsymbol{\rho})$ by the number survived among not-yet-vaccinated individuals $\widehat{N}_{l-1}^{u}(\boldsymbol{\rho})$.

4 Bias of the hazard difference estimator relative to the causal estimand for vaccination at two time points

4.1 Analytic comparison

Consider vaccination at two time points. The hazard difference estimator for quantifying outcomes that could have been directly averted under full vaccination at baseline $\boldsymbol{\psi} = \{2, d; 1{,}0\}$ but were not averted given the particular strategy $\boldsymbol{\rho} = \{2, d; \rho_0, \rho_1\}$ is:

$$\widehat{\delta_2}^{D*}(\boldsymbol{\psi},\boldsymbol{\rho}) = \widehat{N}_0^{u}(\boldsymbol{\rho})\left(\widehat{h}_1^{u}(\boldsymbol{\rho}) - \widehat{h}_1^{v}(\boldsymbol{\rho})\right) + \widehat{N}_1^{u}(\boldsymbol{\rho})\left(\widehat{h}_2^{u}(\boldsymbol{\rho}) - \widehat{h}_2^{v}(\boldsymbol{\rho})\right)$$

$$= N(\rho_1+\rho_2)\left(\frac{\rho_1\Delta\widehat{Y}_1(1;\boldsymbol{\rho}) + \rho_2\Delta\widehat{Y}_1(2;\boldsymbol{\rho})}{\rho_1+\rho_2} - \Delta\widehat{Y}_1(0;\boldsymbol{\rho})\right) + N\rho_2\left[1-\widehat{Y}_1(2;\boldsymbol{\rho})\right]$$

$$\cdot\left(\frac{\Delta\widehat{Y}_2(2;\boldsymbol{\rho})}{1-\widehat{Y}_1(2;\boldsymbol{\rho})} - \frac{\rho_0\Delta\widehat{Y}_2(0;\boldsymbol{\rho}) + \rho_1\Delta\widehat{Y}_2(1;\boldsymbol{\rho})}{\rho_0\left(1-\widehat{Y}_1(0;\boldsymbol{\rho})\right) + \rho_1\left(1-\widehat{Y}_1(1;\boldsymbol{\rho})\right)}\right)$$

$$= N\cdot\Bigg[(\rho_1+\rho_2)\left(\frac{\rho_1\Delta\widehat{Y}_1(1;\boldsymbol{\rho}) + \rho_2\Delta\widehat{Y}_1(2;\boldsymbol{\rho})}{\rho_1+\rho_2} - \Delta\widehat{Y}_1(0;\boldsymbol{\rho})\right) + \rho_2$$

$$\cdot\left(\Delta\widehat{Y}_2(2;\boldsymbol{\rho}) - \left[1-\widehat{Y}_1(2;\boldsymbol{\rho})\right]\cdot\frac{\rho_0\Delta\widehat{Y}_2(0;\boldsymbol{\rho}) + \rho_1\Delta\widehat{Y}_2(1;\boldsymbol{\rho})}{\rho_0\left(1-\widehat{Y}_1(0;\boldsymbol{\rho})\right) + \rho_1\left(1-\widehat{Y}_1(1;\boldsymbol{\rho})\right)}\right)\Bigg]$$

$$= N\cdot\Bigg[\left(\rho_1\Delta\widehat{Y}_1(1;\boldsymbol{\rho}) + \rho_2\Delta\widehat{Y}_1(2;\boldsymbol{\rho}) - (\rho_1+\rho_2)\cdot\Delta\widehat{Y}_1(0;\boldsymbol{\rho})\right) + \rho_2$$

$$\cdot\left(\Delta\widehat{Y}_2(2;\boldsymbol{\rho}) - \frac{(\rho_0+\rho_1)\cdot\left(1-\widehat{Y}_1(2;\boldsymbol{\rho})\right)}{\rho_0\left(1-\widehat{Y}_1(0;\boldsymbol{\rho})\right) + \rho_1\left(1-\widehat{Y}_1(1;\boldsymbol{\rho})\right)}\cdot\frac{\rho_0\Delta\widehat{Y}_2(0;\boldsymbol{\rho}) + \rho_1\Delta\widehat{Y}_2(1;\boldsymbol{\rho})}{\rho_0+\rho_1}\right)\Bigg].$$

Consider $E\left[\widehat{\delta_2}^{D*}(\boldsymbol{\psi},\boldsymbol{\rho})\right]$.

$$E\left[\widehat{\delta_2}^{D*}(\boldsymbol{\psi},\boldsymbol{\rho})\right] = E\Bigg[N \cdot \Bigg[\left(\rho_1\Delta\hat{Y}_1(1;\boldsymbol{\rho}) + \rho_2\Delta\hat{Y}_1(2;\boldsymbol{\rho}) - (\rho_1+\rho_2)\cdot\Delta\hat{Y}_1(0;\boldsymbol{\rho})\right) + \rho_2 \cdot \left(\Delta\hat{Y}_2(2;\boldsymbol{\rho}) - \frac{(\rho_0+\rho_1)\cdot\left(1-\hat{Y}_1(2;\boldsymbol{\rho})\right)}{\rho_0\left(1-\hat{Y}_1(0;\boldsymbol{\rho})\right)+\rho_1\left(1-\hat{Y}_1(1;\boldsymbol{\rho})\right)}\cdot\frac{\rho_0\Delta\hat{Y}_2(0;\boldsymbol{\rho})+\rho_1\Delta\hat{Y}_2(1;\boldsymbol{\rho})}{\rho_0+\rho_1}\right)\Bigg]\Bigg]$$

$$= N\cdot\Bigg\{\left(\rho_1\Delta\bar{Y}_1(1;\boldsymbol{\rho}) + \rho_2\Delta\bar{Y}_1(2;\boldsymbol{\rho})\right) - (\rho_1+\rho_2)\cdot\Delta\bar{Y}_1(0;\boldsymbol{\rho}) + \rho_2\cdot\Delta\bar{Y}_2(2;\boldsymbol{\rho}) - E\left[\rho_2\cdot\left(\frac{(\rho_0+\rho_1)\cdot\left(1-\hat{Y}_1(2;\boldsymbol{\rho})\right)}{\rho_0\left(1-\hat{Y}_1(0;\boldsymbol{\rho})\right)+\rho_1\left(1-\hat{Y}_1(1;\boldsymbol{\rho})\right)}\cdot\frac{\rho_0\Delta\hat{Y}_2(0;\boldsymbol{\rho})+\rho_1\Delta\hat{Y}_2(1;\boldsymbol{\rho})}{\rho_0+\rho_1}\right)\right]\Bigg\} \quad \text{(S24)}$$

Comparing equation (S20) with equation (S24), we know that $E\left[\widehat{\delta_2}^{D*}(\boldsymbol{\psi},\boldsymbol{\rho})\right] \neq \delta_2^D(\boldsymbol{\psi},\boldsymbol{\rho})$ if vaccine has a non-null effect, as equation (S24) misses the term $\rho_1\cdot[\Delta\bar{Y}_2(1;\boldsymbol{\rho}) - \Delta\bar{Y}_2(0;\boldsymbol{\rho})]$ and $E\left[\rho_2\cdot\frac{(\rho_0+\rho_1)\cdot(1-\hat{Y}_1(2;\boldsymbol{\rho}))}{\rho_0(1-\hat{Y}_1(0;\boldsymbol{\rho}))+\rho_1(1-\hat{Y}_1(1;\boldsymbol{\rho}))}\cdot\frac{\rho_0\Delta\hat{Y}_2(0;\boldsymbol{\rho})+\rho_1\Delta\hat{Y}_2(1;\boldsymbol{\rho})}{\rho_0+\rho_1}\right] \neq \rho_2\cdot\Delta\bar{Y}_2(0;\boldsymbol{\rho})$.

4.2 Simulation

We simulate the epidemic under strategy $\boldsymbol{\rho}' = (2,60;0.2,0.3)$ based on the same model (equation [8]), scenarios (**Table 1**), parameters (**Table S4**), and initial conditions (**Table S5**) as with the simulations in the main text. We examine the bias of the hazard difference estimator $\widehat{\delta_2}^{D*}(\boldsymbol{\psi},\boldsymbol{\rho}')$ relative to the causal estimand $\delta_2^D(\boldsymbol{\psi},\boldsymbol{\rho}')$, where $\boldsymbol{\psi} = \{2,60;1,0\}$, and identify the conditions under which the bias would be substantial.

The hazard difference estimator overestimates directly avertible infections when VE_{inf} = 90% (**Figure S6A; Table S13**). However, the hazard difference estimator overestimates directly avertible deaths in Scenarios 3 to 6 and underestimates directly avertible deaths in other scenarios (**Figure S6B; Table S13**).

Take Scenario 3 where IFR=100% as an example. The hazard difference estimator overestimates avertible deaths. Due to high VE_{inf}, survival is lower among $x = 2$ compared to the average of $x = 1$ or $x = 0$ (**Figure S3**). Specifically, $\frac{(\rho_0'+\rho_1')\cdot\left(1-\hat{Y}_1(2;\boldsymbol{\rho}')\right)}{\rho_0'(1-\hat{Y}_1(0;\boldsymbol{\rho}'))+\rho_1'(1-\hat{Y}_1(1;\boldsymbol{\rho}'))} < 1$ in equation (S24), such that $E\left[\rho_2' \cdot \frac{(\rho_0'+\rho_1')\cdot\left(1-\hat{Y}_1(2;\boldsymbol{\rho}')\right)}{\rho_0'(1-\hat{Y}_1(0;\boldsymbol{\rho}'))+\rho_1'(1-\hat{Y}_1(1;\boldsymbol{\rho}'))} \cdot \frac{\rho_0'\Delta\hat{Y}_2(0;\boldsymbol{\rho})+\rho_1'\Delta\hat{Y}_2(1;\boldsymbol{\rho}')}{\rho_0'+\rho_1'}\right]$ in equation (S24) is smaller than $\rho_2' \cdot \Delta\bar{Y}_2(0;\boldsymbol{\rho}')$ in equation (S20).

However, in Scenario 2 where IFR=10%, the hazard difference estimator underestimates avertible deaths because the relative survival is similar (i.e., $\frac{(\rho_0'+\rho_1')\cdot\left(1-\hat{Y}_1(2;\boldsymbol{\rho}')\right)}{\rho_0'(1-\hat{Y}_1(0;\boldsymbol{\rho}'))+\rho_1'(1-\hat{Y}_1(1;\boldsymbol{\rho}'))} \approx 1$), such that $E\left[\rho_2' \cdot \frac{(\rho_0'+\rho_1')\cdot\left(1-\hat{Y}_1(2;\boldsymbol{\rho}')\right)}{\rho_0'(1-\hat{Y}_1(0;\boldsymbol{\rho}'))+\rho_1'(1-\hat{Y}_1(1;\boldsymbol{\rho}'))} \cdot \frac{\rho_0'\Delta\hat{Y}_2(0;\boldsymbol{\rho}')+\rho_1'\Delta\hat{Y}_2(1;\boldsymbol{\rho}')}{\rho_0'+\rho_1'}\right]$ in equation (S24) is greater than $\rho_2' \cdot \Delta\bar{Y}_2(0;\boldsymbol{\rho}')$ in equation (S20).

TABLE S13 | Absolute and percentage bias of the hazard difference estimator relative to the causal estimand (reference) for avertible outcomes under strategy $\boldsymbol{\rho}' = (2{,}60; 0.2, 0.3)$ in scenarios varied by infection-fatality rate (IFR), vaccine efficacy against infection (VE_{inf}) and vaccine efficacy against death given infection (VE_{death})

	**Scenario 1**	**Scenario 2**	**Scenario 3**	**Scenario 4**	**Scenario 5**	**Scenario 6**	**Scenario 7**	**Scenario 8**	**Scenario 9**
	IFR: 1%, VE_{inf} = 90%, VE_{death} = 0%	IFR: 10%, VE_{inf} = 90%, VE_{death} = 0%	IFR: 100%, VE_{inf} = 90%, VE_{death} = 0%	IFR: 1%, VE_{inf} = 0%, VE_{death} = 90%	IFR: 10%, VE_{inf} = 0%, VE_{death} = 90%	IFR: 100%, VE_{inf} = 0%, VE_{death} = 90%	IFR: 1%, VE_{inf} = 90%, VE_{death} = 90%	IFR: 10%, VE_{inf} = 90%, VE_{death} = 90%	IFR: 100%, VE_{inf} = 90%, VE_{death} = 90%
**Avertible infections [a]**	18348.79 (12.02%)	19706.57 (12.92%)	65014.35 (57.86%)	0.00	0.00	0.00	18333.15 (12.01%)	19528.3 (12.79%)	46502.42 (35.06%)
**Avertible deaths [a]**	-300.49 (-19.75%)	-2911.27 (-19.12%)	9526.54 (7.96%)	0.11 (0.01%)	11.35 (0.06%)	1957.46 (1.01%)	-30.05 (-1.73%)	-291.24 (-1.67%)	-132.51 (-0.07%)

[a] Percentage differences were calculated using values rounded to nine decimal places, to avoid uninterpretable, extremely small non-zero values (absolute value < 10^{-9}). All values in the table are rounded to two decimal places.

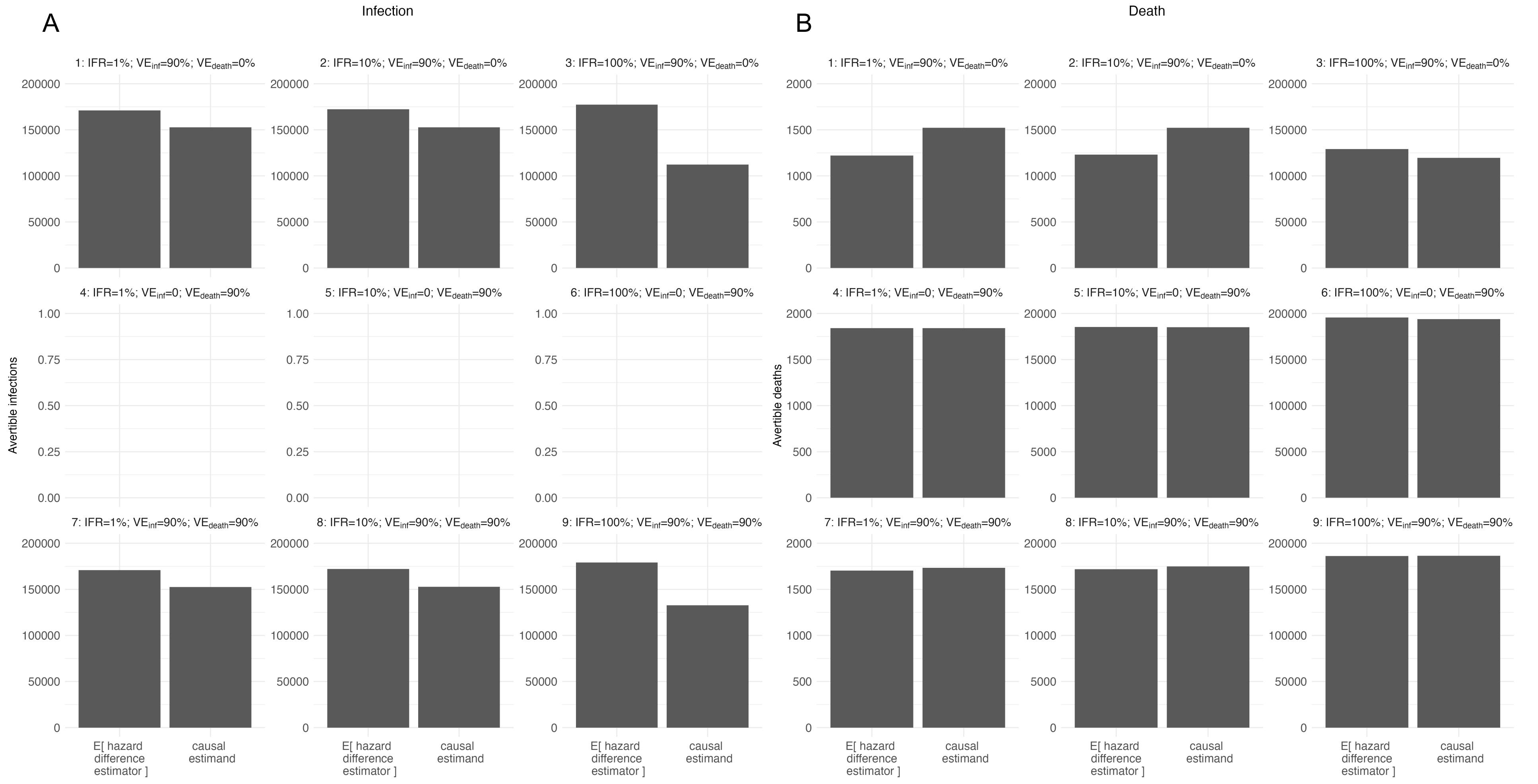

FIGURE S6 | Infections (A) and deaths (B) directly avertible among unvaccinated individuals with strategy $\boldsymbol{\rho}' = (2{,}60; 0.2, 0.3)$ under different scenarios varied by infection-fatality rate (IFR), vaccine efficacy against infection (VE_{inf}) and vaccine efficacy against death given infection (VE_{death})

5 Bias of the hazard difference estimator relative to the causal estimand for vaccination at an arbitrary number of vaccination times

5.1 Analytic comparison

Now consider vaccination at an arbitrary number of vaccination times (i.e., $q \in \mathbb{N}$). First, expand $\delta_{q+1}^{D}(\boldsymbol{\psi}, \boldsymbol{\rho})$ in equation (S21) as:

$$\delta_{q+1}^{D}(\boldsymbol{\psi}, \boldsymbol{\rho}) = N \cdot \sum_{k=1}^{q+1} \rho_k \cdot \left(\bar{Y}_{q+1}(k; \boldsymbol{\rho}) - \bar{Y}_{q+1}(0; \boldsymbol{\rho})\right)$$

$$= N \cdot \sum_{k=1}^{q+1} \rho_k \cdot \left(\sum_{l=1}^{q+1} \Delta\bar{Y}_l(k; \boldsymbol{\rho}) - \sum_{l=1}^{q+1} \Delta\bar{Y}_l(0; \boldsymbol{\rho})\right)$$

$$= N \cdot \sum_{k=1}^{q+1} \rho_k \cdot \left(\Delta\bar{Y}_1(k; \boldsymbol{\rho}) + \sum_{l=2}^{q+1} \Delta\bar{Y}_l(k; \boldsymbol{\rho}) - \Delta\bar{Y}_1(0; \boldsymbol{\rho}) - \sum_{l=2}^{q+1} \Delta\bar{Y}_l(0; \boldsymbol{\rho})\right)$$

$$= N \cdot \left[\sum_{k=1}^{q+1} \rho_k \cdot \left(\Delta\bar{Y}_1(k; \boldsymbol{\rho}) - \Delta\bar{Y}_1(0; \boldsymbol{\rho})\right) + \sum_{k=1}^{q+1} \rho_k \cdot \left(\sum_{l=2}^{q+1} \Delta\bar{Y}_l(k; \boldsymbol{\rho}) - \sum_{l=2}^{q+1} \Delta\bar{Y}_l(0; \boldsymbol{\rho})\right)\right] \quad \text{(S25)}$$

Then, expand $\widehat{\delta_{q+1}}^{D*}(\boldsymbol{\psi}, \boldsymbol{\rho})$ in equation (S23) as:

$$\widehat{\delta_{q+1}}^{D*}(\boldsymbol{\psi}, \boldsymbol{\rho}) = \sum_{l=1}^{q+1} \left[\hat{N}_{l-1}^{u}(\boldsymbol{\rho}) \cdot \left(\hat{h}_l^{u}(\boldsymbol{\rho}) - \hat{h}_l^{v}(\boldsymbol{\rho})\right)\right]$$

$$= \sum_{l=1}^{q+1} \left\{N\left[\sum_{k=l}^{q+1} \rho_k \left(1 - \hat{Y}_{l-1}(k; \boldsymbol{\rho})\right)\right] \cdot \left[\frac{\sum_{k=l}^{q+1} \rho_k \Delta\hat{Y}_l(k; \boldsymbol{\rho})}{\sum_{k=l}^{q+1} \rho_k \left(1 - \hat{Y}_{l-1}(k; \boldsymbol{\rho})\right)} - \frac{\sum_{k=0}^{l-1} \rho_k \Delta\hat{Y}_l(k; \boldsymbol{\rho})}{\sum_{k=0}^{l-1} \rho_k \left(1 - \hat{Y}_{l-1}(k; \boldsymbol{\rho})\right)}\right]\right\}$$

$$= N \sum_{l=1}^{q+1} \left\{\sum_{k=l}^{q+1} \rho_k \Delta\hat{Y}_l(k; \boldsymbol{\rho}) - \left[\sum_{k=l}^{q+1} \rho_k \left(1 - \hat{Y}_{l-1}(k; \boldsymbol{\rho})\right)\right] \cdot \frac{\sum_{k=0}^{l-1} \rho_k \Delta\hat{Y}_l(k; \boldsymbol{\rho})}{\sum_{k=0}^{l-1} \rho_k \left(1 - \hat{Y}_{l-1}(k; \boldsymbol{\rho})\right)}\right\}$$

Pulling $l = 1$ from the summation →

$$= N\left\{\sum_{k=1}^{q+1} \rho_k \Delta\hat{Y}_1(k; \boldsymbol{\rho}) - \left(\sum_{k=1}^{q+1} \rho_k\right) \cdot \Delta\hat{Y}_1(0; \boldsymbol{\rho})\right.$$

$$\left. + \sum_{l=2}^{q+1} \left\{\sum_{k=l}^{q+1} \rho_k \Delta\hat{Y}_l(k; \boldsymbol{\rho}) - \left[\sum_{k=l}^{q+1} \rho_k \left(1 - \hat{Y}_{l-1}(k; \boldsymbol{\rho})\right)\right] \cdot \frac{\sum_{k=0}^{l-1} \rho_k \Delta\hat{Y}_l(k; \boldsymbol{\rho})}{\sum_{k=0}^{l-1} \rho_k \left(1 - \hat{Y}_{l-1}(k; \boldsymbol{\rho})\right)}\right\}\right\}$$

$$= N\left\{\sum_{k=1}^{q+1}\rho_k\left(\Delta\hat{Y}_1(k;\boldsymbol{\rho})-\Delta\hat{Y}_1(0;\boldsymbol{\rho})\right)+\sum_{l=2}^{q+1}\left[\sum_{k=l}^{q+1}\rho_k\Delta\hat{Y}_l(k;\boldsymbol{\rho})-\left(\sum_{k=l}^{q+1}\rho_k\right)\cdot\frac{\frac{\sum_{k=l}^{q+1}\rho_k\left(1-\hat{Y}_{l-1}(k;\boldsymbol{\rho})\right)}{\sum_{k=l}^{q+1}\rho_k}}{\frac{\sum_{k=0}^{l-1}\rho_k\left(1-\hat{Y}_{l-1}(k;\boldsymbol{\rho})\right)}{\sum_{k=0}^{l-1}\rho_k}}\cdot\frac{\sum_{k=0}^{l-1}\rho_k\Delta\hat{Y}_l(k;\boldsymbol{\rho})}{\sum_{k=0}^{l-1}\rho_k}\right]\right\}.$$

Now consider $E\left[\widehat{\delta_{q+1}}^{D*}(\boldsymbol{\psi},\boldsymbol{\rho})\right]$.

$$E\left[\widehat{\delta_{q+1}}^{D*}(\boldsymbol{\psi},\boldsymbol{\rho})\right]=E\left[N\left\{\sum_{k=1}^{q+1}\rho_k\left(\Delta\hat{Y}_1(k;\boldsymbol{\rho})-\Delta\hat{Y}_1(0;\boldsymbol{\rho})\right)\right.\right.$$

$$\left.\left.+\sum_{l=2}^{q+1}\left[\sum_{k=l}^{q+1}\rho_k\Delta\hat{Y}_l(k;\boldsymbol{\rho})-\left(\sum_{k=l}^{q+1}\rho_k\right)\cdot\frac{\frac{\sum_{k=l}^{q+1}\rho_k\left(1-\hat{Y}_{l-1}(k;\boldsymbol{\rho})\right)}{\sum_{k=l}^{q+1}\rho_k}}{\frac{\sum_{k=0}^{l-1}\rho_k\left(1-\hat{Y}_{l-1}(k;\boldsymbol{\rho})\right)}{\sum_{k=0}^{l-1}\rho_k}}\cdot\frac{\sum_{k=0}^{l-1}\rho_k\Delta\hat{Y}_l(k;\boldsymbol{\rho})}{\sum_{k=0}^{l-1}\rho_k}\right]\right\}\right]$$

Using arguments parallel with those in the proof of Theorem S2 $\longrightarrow$

$$= N\cdot\left\{\left[\sum_{k=1}^{q+1}\rho_k\cdot\left(\Delta\bar{Y}_1(k;\boldsymbol{\rho})-\Delta\bar{Y}_1(0;\boldsymbol{\rho})\right)\right]\right.$$

$$\left.+\sum_{l=2}^{q+1}\left[\sum_{k=l}^{q+1}\rho_k\Delta\bar{Y}_l(k;\boldsymbol{\rho})\right]-E\left[\sum_{l=2}^{q+1}\left[\left(\sum_{k=l}^{q+1}\rho_k\right)\cdot\frac{\frac{\sum_{k=l}^{q+1}\rho_k\left(1-\hat{Y}_{l-1}(k;\boldsymbol{\rho})\right)}{\sum_{k=l}^{q+1}\rho_k}}{\frac{\sum_{k=0}^{l-1}\rho_k\left(1-\hat{Y}_{l-1}(k;\boldsymbol{\rho})\right)}{\sum_{k=0}^{l-1}\rho_k}}\cdot\frac{\sum_{k=0}^{l-1}\rho_k\Delta\hat{Y}_l(k;\boldsymbol{\rho})}{\sum_{k=0}^{l-1}\rho_k}\right]\right]\right\}\quad\text{(S26)}$$

Equation (S25) and equation (S26) have the same first term. However, if vaccination has a non-null effect, $\sum_{k=1}^{q+1}\rho_k\cdot\left(\sum_{l=2}^{q+1}\Delta\bar{Y}_l(k;\boldsymbol{\rho})-\sum_{l=2}^{q+1}\Delta\bar{Y}_l(0;\boldsymbol{\rho})\right)=\sum_{k=1}^{q+1}\rho_k\cdot\sum_{l=2}^{q+1}\Delta\bar{Y}_l(k;\boldsymbol{\rho})-\sum_{k=1}^{q+1}\rho_k\cdot\sum_{l=2}^{q+1}\Delta\bar{Y}_l(0;\boldsymbol{\rho})$ in equation (S25) does not equal to $\sum_{l=2}^{q+1}\left[\sum_{k=l}^{q+1}\rho_k\Delta\bar{Y}_l(k;\boldsymbol{\rho})\right]-E\left[\sum_{l=2}^{q+1}\left[\left(\sum_{k=l}^{q+1}\rho_k\right)\cdot\frac{\frac{\sum_{k=l}^{q+1}\rho_k\left(1-\hat{Y}_{l-1}(k;\boldsymbol{\rho})\right)}{\sum_{k=l}^{q+1}\rho_k}}{\frac{\sum_{k=0}^{l-1}\rho_k\left(1-\hat{Y}_{l-1}(k;\boldsymbol{\rho})\right)}{\sum_{k=0}^{l-1}\rho_k}}\cdot\frac{\sum_{k=0}^{l-1}\rho_k\Delta\hat{Y}_l(k;\boldsymbol{\rho})}{\sum_{k=0}^{l-1}\rho_k}\right]\right]$ in equation (S26), implying $E\left[\widehat{\delta_{q+1}}^{D*}(\boldsymbol{\psi},\boldsymbol{\rho})\right]\neq\delta_{q+1}^{D}(\boldsymbol{\psi},\boldsymbol{\rho})$.

5.2 Simulation

We simulate the epidemic under strategy $\boldsymbol{\rho}'' = \{99,7; \mathbf{0.01}\}$ based on the model in equation (S14), scenarios in **Table 1**, initial values in **Table S2** and parameters in **Table S4**. Similar to findings observed under strategy $\boldsymbol{\rho}'$, the hazard difference estimator overestimates directly avertible infections when VE_{inf}=90% (**Figure S7A, Table S14**). It substantially overestimates directly avertible deaths when IFR=100% and VE_{death} = 0% (Scenario 3), slightly overestimates them in Scenarios 4, 5, 6 and 9, and underestimates them in Scenarios 1, 2, 7, and 8 (**Figure S7B, Table S14**).

TABLE S14 | Absolute and percentage bias of the hazard difference estimator relative to the causal estimand for avertible outcomes under strategy $\boldsymbol{\rho}'' = \{99{,}7; \mathbf{0.01}\}$ in scenarios varied by infection-fatality rate (IFR), vaccine efficacy against infection (VE_{inf}) and vaccine efficacy against death given infection (VE_{death})

	**Scenario 1**	**Scenario 2**	**Scenario 3**	**Scenario 4**	**Scenario 5**	**Scenario 6**	**Scenario 7**	**Scenario 8**	**Scenario 9**
	IFR: 1%, VE_{inf} = 90%, VE_{death} = 0%	IFR: 10%, VE_{inf} = 90%, VE_{death} = 0%	IFR: 100%, VE_{inf} = 90%, VE_{death} = 0%	IFR: 1%, VE_{inf} = 0%, VE_{death} = 90%	IFR: 10%, VE_{inf} = 0%, VE_{death} = 90%	IFR: 100%, VE_{inf} = 0%, VE_{death} = 90%	IFR: 1%, VE_{inf} = 90%, VE_{death} = 90%	IFR: 10%, VE_{inf} = 90%, VE_{death} = 90%	IFR: 100%, VE_{inf} = 90%, VE_{death} = 90%
**Avertible infections** [a]	48041.54 (25.08%)	51050.02 (26.9%)	183221.65 (295.78%)	0.00	0.00	0.00	48034.29 (25.07%)	50967.59 (26.85%)	144266.72 (142.84%)
**Avertible deaths** [a]	-316.36 (-16.51%)	-2814.54 (-14.83%)	133578.07 (215.64%)	0.7 (0.03%)	72.59 (0.3%)	9981.36 (4.02%)	-31.53 (-1.22%)	-270.1 (-1.04%)	10765.53 (4.17%)

[a] Percentage differences were calculated using values rounded to nine decimal places, to avoid uninterpretable, extremely small non-zero values (absolute value $< 10^{-9}$). All values in the table are rounded to two decimal places

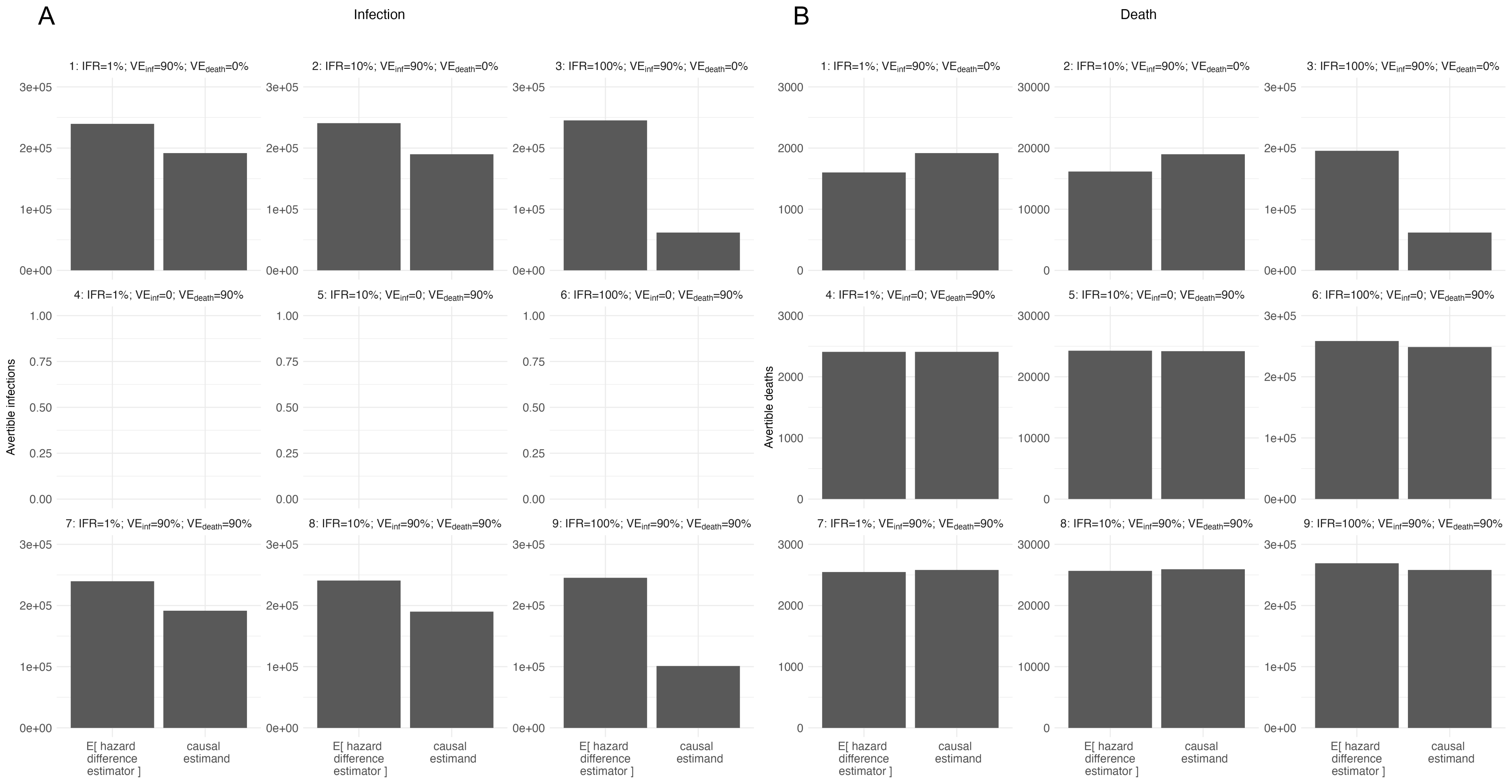

FIGURE S7 | Infections (A) and deaths (B) directly avertible among unvaccinated individuals with strategy $\boldsymbol{\rho}'' = \{99{,}7; \mathbf{0.01}\}$ under different scenarios varied by infection-fatality rate (IFR), vaccine efficacy against infection (VE_{inf}) and vaccine efficacy against death given infection (VE_{death})

References

1. Haas EJ, McLaughlin JM, Khan F, et al. Infections, hospitalisations, and deaths averted via a nationwide vaccination campaign using the Pfizer–BioNTech BNT162b2 mRNA COVID-19 vaccine in Israel: a retrospective surveillance study. *The Lancet Infectious Diseases*. 2022;22(3):357-366. doi:10.1016/S1473-3099(21)00566-1

2. Brault A, Hart A, Uribe P, et al. Direct impact of COVID-19 vaccination in Chile: averted cases, hospitalizations, ICU admissions, and deaths. *BMC Infect Dis*. 2024;24(1):467. doi:10.1186/s12879-024-09304-1

3. Santos CVBD, Noronha TGD, Werneck GL, Struchiner CJ, Villela DAM. Estimated COVID-19 severe cases and deaths averted in the first year of the vaccination campaign in Brazil: a retrospective observational study. *The Lancet Regional Health - Americas*. 2023;17:100418. doi:10.1016/j.lana.2022.100418

4. Kayano T, Sasanami M, Kobayashi T, et al. Number of averted COVID-19 cases and deaths attributable to reduced risk in vaccinated individuals in Japan. *The Lancet Regional Health - Western Pacific*. 2022;28:100571. doi:10.1016/j.lanwpc.2022.100571

5. Kayano T, Nishiura H. Assessing the COVID-19 vaccination program during the Omicron variant (B.1.1.529) epidemic in early 2022, Tokyo. *BMC Infect Dis*. 2023;23(1):748. doi:10.1186/s12879-023-08748-1

6. Vilches TN, Moghadas SM, Sah P, et al. Estimating COVID-19 Infections, Hospitalizations, and Deaths Following the US Vaccination Campaigns During the Pandemic. *JAMA Netw Open*. 2022;5(1):e2142725. doi:10.1001/jamanetworkopen.2021.42725

7. Schneider EC, Shah A, Sah P, et al. Impact of U.S. COVID-19 Vaccination Efforts: An Update on Averted Deaths, Hospitalizations, and Health Care Costs Through March 2022. To the Point. April 8, 2022. Accessed December 10, 2023. https://doi.org/10.26099/d3dm-fa91

8. Gavish N, Yaari R, Huppert A, Katriel G. Population-level implications of the Israeli booster campaign to curtail COVID-19 resurgence. *Sci Transl Med*. 2022;14(647):eabn9836. doi:10.1126/scitranslmed.abn9836

9. Watson OJ, Barnsley G, Toor J, Hogan AB, Winskill P, Ghani AC. Global impact of the first year of COVID-19 vaccination: a mathematical modelling study. *The Lancet Infectious Diseases*. 2022;22(9):1293-1302. doi:10.1016/S1473-3099(22)00320-6

10. Shoukat A, Vilches TN, Moghadas SM, et al. Lives saved and hospitalizations averted by COVID-19 vaccination in New York City: a modeling study. *The Lancet Regional Health - Americas*. 2022;5:100085. doi:10.1016/j.lana.2021.100085

11. Hudgens MG, Halloran ME. Toward Causal Inference With Interference. *Journal of the American Statistical Association*. 2008;103(482):832-842. doi:10.1198/016214508000000292

12. Sobel ME. What Do Randomized Studies of Housing Mobility Demonstrate?: Causal Inference in the Face of Interference. *Journal of the American Statistical Association*. 2006;101(476):1398-1407. doi:10.1198/016214506000000636

13. VanderWeele TJ, Tchetgen Tchetgen EJ. Effect partitioning under interference in two-stage randomized vaccine trials. *Statistics & Probability Letters*. 2011;81(7):861-869. doi:10.1016/j.spl.2011.02.019

14. Jia KM, Boyer CB, Wallinga J, Lipsitch M. Causal Estimands for Analyses of Averted and Avertible Outcomes due to Infectious Disease Interventions. *Epidemiology*. 2025;36(3):363-373. doi:10.1097/EDE.0000000000001839

15. Forastiere L, Airoldi EM, Mealli F. Identification and Estimation of Treatment and Interference Effects in Observational Studies on Networks. *Journal of the American Statistical Association*. 2021;116(534):901-918. doi:10.1080/01621459.2020.1768100

16. Yang W, Lipsitch M, Shaman J. Inference of seasonal and pandemic influenza transmission dynamics. *Proc Natl Acad Sci USA*. 2015;112(9):2723-2728. doi:10.1073/pnas.1415012112

17. Guerra FM, Bolotin S, Lim G, et al. The basic reproduction number (R 0) of measles: a systematic review. *The Lancet Infectious Diseases*. 2017;17(12):e420-e428. doi:10.1016/S1473-3099(17)30307-9

18. Locatelli I, Trächsel B, Rousson V. Estimating the basic reproduction number for COVID-19 in Western Europe. Khudyakov YE, ed. *PLoS ONE*. 2021;16(3):e0248731. doi:10.1371/journal.pone.0248731

19. Nguyen VH, Crépey P, Williams BA, et al. Modeling the impact of early vaccination in an influenza pandemic in the United States. *npj Vaccines*. 2025;10(1):62. doi:10.1038/s41541-025-01081-5

20. Portnoy A, Jit M, Ferrari M, Hanson M, Brenzel L, Verguet S. Estimates of case-fatality ratios of measles in low-income and middle-income countries: a systematic review and modelling analysis. *The Lancet Global Health*. 2019;7(4):e472-e481. doi:10.1016/S2214-109X(18)30537-0

21. Ayoub HH, Mumtaz GR, Seedat S, Makhoul M, Chemaitelly H, Abu-Raddad LJ. Estimates of global SARS-CoV-2 infection exposure, infection morbidity, and infection mortality rates in 2020. *Global Epidemiology*. 2021;3:100068. doi:10.1016/j.gloepi.2021.100068

22. Centers for Disease Control and Prevention. *U.S. Flu Vaccine Effectiveness (VE) Data for 2021-2022.*; 2024. Accessed July 3, 2025. https://www.cdc.gov/flu-vaccines-work/php/effectiveness-studies/2021-2022.html

23. Di Pietrantonj C, Rivetti A, Marchione P, Debalini MG, Demicheli V. Vaccines for measles, mumps, rubella, and varicella in children. Cochrane Acute Respiratory Infections Group, ed. *Cochrane Database of Systematic Reviews*. 2021;2021(11). doi:10.1002/14651858.CD004407.pub5

24. Polack FP, Thomas SJ, Kitchin N, et al. Safety and Efficacy of the BNT162b2 mRNA Covid-19 Vaccine. *N Engl J Med*. 2020;383(27):2603-2615. doi:10.1056/NEJMoa2034577

25. Ferdinands JM, Thompson MG, Blanton L, Spencer S, Grant L, Fry AM. Does influenza vaccination attenuate the severity of breakthrough infections? A narrative review and recommendations for further research. *Vaccine*. 2021;39(28):3678-3695. doi:10.1016/j.vaccine.2021.05.011

26. Nic Lochlainn LM, De Gier B, Van Der Maas N, et al. Immunogenicity, effectiveness, and safety of measles vaccination in infants younger than 9 months: a systematic review and meta-analysis. *The Lancet Infectious Diseases*. 2019;19(11):1235-1245. doi:10.1016/S1473-3099(19)30395-0

27. Chemaitelly H, Tang P, Hasan MR, et al. Waning of BNT162b2 Vaccine Protection against SARS-CoV-2 Infection in Qatar. *N Engl J Med*. 2021;385(24). doi:10.1056/NEJMoa2114114

28. Cauchemez S, Ferguson NM. Likelihood-based estimation of continuous-time epidemic models from time-series data: application to measles transmission in London. *J R Soc Interface*. 2008;5(25):885-897. doi:10.1098/rsif.2007.1292

29. Della Rossa F, Salzano D, Di Meglio A, et al. A network model of Italy shows that intermittent regional strategies can alleviate the COVID-19 epidemic. *Nat Commun*. 2020;11(1):5106. doi:10.1038/s41467-020-18827-5

30. Centers for Disease Control and Prevention. Rates of COVID-19 Cases or Deaths by Age Group and Vaccination Status. Published online 2025. Accessed August 14, 2025. https://data.cdc.gov/Public-Health-Surveillance/Rates-of-COVID-19-Cases-or-Deaths-by-Age-Group-and/3rge-nu2a/about_data

31. Hernán MA, Sterne JAC, Higgins JPT, Shrier I, Hernández-Díaz S. A Structural Description of Biases That Generate Immortal Time. *Epidemiology*. 2025;36(1):107-114. doi:10.1097/EDE.0000000000001808

32. Jia KM, Hanage WP, Lipsitch M, et al. Estimated preventable COVID-19-associated deaths due to non-vaccination in the United States. *Eur J Epidemiol*. Published online April 24, 2023. doi:10.1007/s10654-023-01006-3

33. Zhong M, Glazer T, Kshirsagar M, et al. Estimating Vaccine-Preventable COVID-19 Deaths Among Adults Under Counterfactual Vaccination Scenarios in The United States: A Modeling Study Using Observational Data. *J Pharm Pharmacol Res*. 2023;07(03). doi:10.26502/fjppr.079
